# Supplementary material for: PRDM9 drives the location and rapid evolution of recombination hotspots in salmonid fish
Source: PLoS Biol. 2025 Jan 6;23(1):e3002950. doi: 10.1371/journal.pbio.3002950 (PMC11703093; doi:10.1371/journal.pbio.3002950)
Supplement: S1 Methods — (DOCX) [file pbio.3002950.s001.docx]

**Supporting information**

# S1 Methods. Molecular, genomic and population genetics methods.

## Phylogenetic analysis of PRDM9 paralogs across Salmonids

We investigated the presence of a complete PRDM9 protein in twelve species from the three salmonid subfamilies (Coregoninae, Thymallinae, Salmoninae). We deduced a reference protein sequence for the three canonical domains of PRDM9 in the coho salmon: KRAB (encoded by 2 exons), SSXRD (1 exon) and SET (3 exons). This was obtained from a nearly full-length CDS annotated in the RefSeq database (XP_020359152.1), complemented in its 3’ end using a cDNA identified in a brain RNAseq dataset sequenced with PacBio long reads (SRR10185924.264665.1). The nucleotide sequence of the 6 exons was retrieved as the best tblastn (1) hit of the protein sequence against the *O. kisutch* reference genome. This reference sequence was subsequently used to identify PRDM9 homologs in the whole genome assembly of the lake whitefish (*Coregonus clupeaformis*, assembly ASM2061545v1, RefSeq accession number GCF_020615455.1), the European grayling (*Thymallus thymallus*, fThyThy.pri.20220222, GenBank accession number GCA_023634145.1), the huchen (*Hucho hucho*, ASM331708v1, GCA_003317085.1), the coho salmon (*O. kisutch*, Okis_V2, GCF_002021735.2), the rainbow trout (*O. mykiss*, Omyk_1.0, GCF_002163495.1 and OmykA_1.1, GCF_013265735.2), the chinook salmon (*O. tschawytscha*, Otsh_v2.0, GCF_018296145.1), the chum salmon (*O. keta*, Oket_V2, GCF_023373465.1), the red salmon (*O. nerka*, Oner_1.1, GCF_006149115.2), the pink salmon (*O. gorbuscha*, OgorEven_v1.0, GCF_021184085.1), the Atlantic salmon (*S. salar*, Ssal_v3.1, GCF_905237065.1), the brown trout (*S. trutta*, fSalTru1.2, GCA_901001165.2), the lake trout (*Salvelinus namaycush*, GCF_016432855.1), as well as a closely related outgroup, the northern pike (*Esox lucius*, fEsoLuc1.pri, GCF_011004845, Esocidae), and the sea bass (*Dicentrarchus labrax*, dlabrax2021, GCF_905237075.1). The six exons were independently blasted to the 14 assemblies with both blastn and tblastn allowing multiple hits. We filtered out copies containing only one of the six exons. We used annotated *Prdm* family genes from the human (GRCh38.p14, GCF_000001405.40) and mouse (GRCm39, GCF_000001635.27) Ensembl genomes to remove non-*Prdm9* copies. Retained exons were aligned separately using Macse (v2.06) (2), a program of coding sequence alignment simultaneously accounting for the nucleotide and amino-acid levels, thus potentially allowing for the inclusion of frameshifts and stop codons. The alignments were manually examined and edited before concatenating exons of the same copy using Amas concat (3). Salmonids have undergone two recent whole duplication (WGD) events that occurred in the common ancestor of teleosts (Ts3R, c.a. 320 Mya) and salmonids (Ss4R, c.a. 90 Mya) respectively (4-6). Consequently, their chromosomes appeared as duplicate pairs referred to as ohnolog chromosomes, derived from the same ancestral chromosome. We therefore retrieved the chromosomal locations of the retained copies in order to trace the evolutionary history of Prdm9 duplications. A maximum-likelihood phylogeny of the three canonical domains was built using IQ-TREE (7) based on amino-acid alignments, using ultrafast bootstrap with 1000 replicates. In order to identify functional *Prdm9* copies with sequence orthology to the 10 exons found in human and mouse (8), we finally predicted the gene structure of each copy surrounded by its 10 kb flanking regions using Genewise (v2.4.1) (9). We selected representative paralogous sequences across the obtained PRDM9 phylogenetic tree (accession number: XP_036826454.1, XP_055786392.1, XP_014035317.2, XP_055791730.1, XP_036838644.1) to perform a sequence similarity-based annotation of the copies in each species.

## Analysis of PRDM9 ZF diversity in rainbow trout and Atlantic salmon

### Samples used for Prdm9α Zinc Finger Array genotyping

We characterized the allelic diversity of the ZF array domain of *Prdm9*α in two species: *S. salar* and *O. mykiss*. We focused on *Prdm9α*, as *Prdm9β* orthologs in teleost fish were previously shown to lack KRAB and SSXRD domains, to have a slowly evolving ZF array and to carry a presumably inactive SET domain (10). We used wild Atlantic salmon (*S. salar*) samples from Normandy (France) that were biopsied with caudal fin clips during routine monitoring and stored in ethanol at -20°C. Samples were kindly provided by Guillaume Evanno (INRAE). We also analyzed rainbow trout (*O. mykiss*) samples of unknown genetic origin that came from a population acquired by the INRAE in the 1990s and selectively bred for fall spawning (ID: INRA-AUT) at an INRAE experimental fish farm (PEIMA). Trout samples were generously supplied by Jean-Jacques Lareyre (INRAE). Genomic DNA was extracted from fin clips using the Qiagen DNeasy Blood & Tissue kit following the manufacturer's instructions. DNA concentration and purity were measured with a Nanodrop-1000 Spectrophotometer (Thermo Fisher Scientific), and quality was assessed via agarose gel electrophoresis. 10 ng/μl working dilutions were prepared and stored at -20 °C.

### Amplification and sequencing of Prdm9α Zinc Finger Array

We inferred the expression levels of multiple *Prdm9α* paralogs in immature testes from both genera, *Salmo* and *Oncorhynchus*, using publicly available RNA-seq data from SRA. Specifically, we analyzed data from two samples in *S. salar* (SRR1422872 and SRR9593306) two samples in *O. kisutch* (SRR8177981 and SRR2157188), and one sample in *O. mykiss* (SRR5657606). Our analysis revealed high expression of two distinct *Prdm9α* paralogs in both genera, which were previously identified in the phylogenetic analysis. Namely, we then sequenced *Prdm9α* paralog *α1.a.2* (full length, chromosome 5, n=26) and *α2.2* (partial, chromosome 17, n=20) in *S. salar*, and *Prdm9α* paralog *α1.a.1* (full length, chromosome 31, n=23) and *α2.2* (full length, chromosome 7, n=20) in *O. mykiss*. The complete list of samples used in this study is available in **S7 Table**.

Primers were designed using NCBI Primer Blast, ensuring specificity against the reference assemblies (*S.salar* ICSASG_v2 and *O. mykiss* USDA_OmykA_1.1). They targeted the ZF sequence encoded in the last exon of the gene, framed by the flanking arms of the array, avoiding any specificity of the paralogous loci. The primers were synthetized by Eurofins Genomics (Ebersberg, Germany). For primers sequences refer to **S8** **Table**.

PCR reactions were carried out in 25 μl volume containing 1X Phusion HF buffer, 200 µM dNTPs, 0.5 µM forward primer, 0.5 µM reverse primer, 3% DMSO, 2.5-10 ng of template and 0.5 units of Phusion Polymerase (NEB). Cycling conditions were: initial denaturation at 98 °C for 2 min followed by 35 cycles of 98 °C 10 s, 66-70 °C 30 s, 72 °C 1:30 min, a final elongation at 72 °C 3 min, and hold at 10 °C in a C1000 Cycler (Bio-Rad). PCR products were inspected on a 1% agarose gel stained with 0.5 μg/ml ethidium bromide. Samples were re-amplified if the amplification was not efficient or in case smearing was observed. Samples showing a single size amplicon, were purified using the NucleoSpin Gel and PCR clean-up kit (Machery-Nagel) and Sanger sequenced in 5’ and 3’ direction. Samples showing two different length alleles were separated by electrophoresis, each single band was purified and cloned using the TOPO Blunt cloning kit (Invitrogen). The ligated vector was then transformed into OneShotTop10 chemically competent cells (Invitrogen), following the manufacturer’s instructions. At least 4 clones per amplicon were purified using the QIAprep Spin Miniprep Kit (Qiagen) and they were Sanger sequenced either using the same primers used to sequence the PCR products or, plasmid specific primers M13 forward and reverse. When Sanger sequencing revealed heterozygosity in the chromatograms from single-sized PCR product, the remaining amplicon was cloned and sequenced as just described for heterozygous samples. All the sequencing was conducted by Azenta-GeneWiz (Leipzig, Germany).

### Allelic diversity analysis

The sequencing results were processed as follows. Forward and reverse reads were *de-novo* assembled to generate contigs and each contig was aligned to the reference ZF array using SnapGene software (versions 5.1.4.1 - 5.2.3). Each contig was translated either using SnapGene, or ExPaSy Translate tool (https://web.expasy.org/translate/) and the single letter amino acid sequence corresponding to each ZF array was recorded, this allowed us to categorize individual PRDM9α alleles. All ZF arrays were annotated such that they matched a C2H2 ZF motif: X7-CXXC-X12-HXXXH, where X is any amino acid, C indicates the cysteines and H the histidine residues. We reported a new allele whenever a single variation in the array at the level of the amino acid sequence was found. DNA sequences for each allele were aligned to create a consensus sequence.

We then determined the proportion of amino acid diversity at DNA-binding residues of the ZF array (positions -1, 2, 3, and 6 of the α-helix) as in (10, 11). The ZF arrays were adapted to match the following motif for the alignment: X2-CXXC-X12-HXXXH-X5. Briefly, we calculated the amino acid diversity as a function of amino acid position in the ZFs array. A single ZF array was analyzed for each paralog by pooling all ZF types identified, except for the partial ZFs (of type X2-CXXC-X12-H-X9). The diversity plots were generated by plotting the heterozygosity values reported at each site of the ZF array. We calculated the proportion of the total amino acid diversity (r) at DNA-binding sites as the sum of heterozygosity at DNA-binding residues over the sum of heterozygosity at all 28 residues of the array.

## Identification of DSB hotspots using ChIP-Seq in the rainbow trout

### Samples used for ChIP-seq experiments

We investigated the genome-wide distribution of DMC1-bound ssDNA in *O. mykiss* testes by chromatin-immunoprecipitation (ChIP) followed by ssDNA enrichment (DMC1-Single Strand DNA Sequencing, DMC1-SSDS). We chose three rainbow trout individuals from the pool of samples previously used for characterizing PRDM9 ZF diversity. We assessed the gonadal stage of the samples, selecting those at an optimal meiotic stage for further analysis. Male gonads were excised, with the majority immediately snap-frozen in liquid nitrogen and stored at -80°C for subsequent processing. A smaller portion was fixed in Bouin’s solution, dehydrated through increasingly concentrated ethanol solutions and finally imbedded in paraplast. 7 µm-thick tissue sections were stained with hematoxylin/eosin staining solutions (Diapath) and mounted using the afcolene mounting medium. The stage of the gonadal maturation was determined from macroscopic and histological observations of the gonads and gonadal sections, respectively, according to (12). In testes at stage I, only undifferentiated A spermatogonia are observed. In stage II testes, testicular lobules become organized and many B spermatogonia are present in addition to A spermatogonia. Testes at stages III and IV increase in size and contain an increasing number of primary spermatocytes including leptotene, zygotene, and pachytene spermatocytes. Few spermatids appear concomitantly. Testes at stages V to VI continue to grow and become whitish due the rapid increasing accumulation of spermatids and spermatozoa, respectively. At stage VII, the collector tube becomes dilated and fills will sperm. At stage VIII, testes reach their maximum size and only spermatozoa are observed in the lumen of the testicular lobules (spermiation). To validate the presence of meiotic cells in stage III testes, immunostaining against SYCP3, SMC3 and DMC1 was carried out on tissue sections (S26 Fig). We used testes at stages III and IV to perform the chromatin immunoprecipitation experiments. We thus selected three individuals with different *Prdm9* genotypes (TAC-1: *Prdm9^1/5^*, stage III; TAC-3: *Prdm9^2/6^*, stage III; and RT-52: *Prdm9^1/2^*, stage IV), enabling the comparison of DSB hotspots between individuals sharing or not sharing a *Prdm9* allele.

##

### Antibodies

Antibodies against DMC1 were from Yukiko Imai (NIG). Briefly, the antibodies were raised by immunizing two rabbits and one guinea pig with a His-tagged recombinant protein of *Danio rerio* Dmc1, corresponding to amino-acid residues 7-220 (UniProt ID: B3DIR0). Dmc1 polyclonal serum was then affinity-purified by using CNBr Activated Sepharose™ 4B (Cytiva) conjugated with the Dmc1 recombinant protein. The obtained rabbit1, rabbit2 and guinea pig anti-Dmc1, were tested by western blot on protein extracts from trout testes (result not shown) to select the most specific one. For DMC1 ChIP, we used either rabbit1 or guinea pig anti-Dmc1, for H3K4me3 ChIP we used rabbit anti-H3K4me3 (Abcam, ab8580) and for H3K36me3 ChIP we used rabbit anti-H3K36me3 (Diagenode, Premium, C15410192).

### Immunostaining

The gonads were dissected from a *O. mykiss* individual, fixed in 4% paraformaldehyde, and cut into 7 μm thick cryosections. Slides were deparaffinized, rehydrated, processed for antigen retrieval by heating for 20 min at 98 °C in 10mM Sodium Citrate, 0.05% Tween 20, pH 6.0 (13), then rinsed in PBS. Immunostaining was performed as previously described (14), using a BSA-based blocking buffer (2% BSA in 1X PBS). Antibodies used were rabbit anti-SYCP3 (Abcam, ab15093) at 1:100 dilution, rabbit anti-SMC3 (Abcam, ab9263) at 1:100 dilution, and rabbit anti-Dmc1 (Yukiko Imai, rabbit1) at 1:200 dilution. All incubations with primary antibodies were performed overnight at room temperature. Secondary antibody was goat anti-rabbit AF555 (Abcam, ab150086), used at 1:200 dilution and incubated for 2 hours at room temperature. Nuclei were labelled with DAPI (4′-6-Diamidino-2-phenylindole, 1 µg/ml) for 5 minutes and cells were analyzed by fluorescence microscopy. Widefield images were acquired using a Zeiss Axioimager apotome microscope with a 63X Plan Apochromat 1.4 NA oil objective and a Zeiss CCD AxioCam MRm 1.4 MP monochrome camera (1388 x 1040 pixels, 6.45μm pixel size).

### Chromatin immunoprecipitation

We conducted crosslink ChIP experiments for H3K4me3 and H3K36me3 using protocols described (15, 16), with some adjustments. Briefly, a portion of frozen tissue (20-25 mg) was immediately immersed in PBS, 1% formaldehyde for 10 min at room temperature. After quenching the unbound PFA by addition of glycine to a final concentration of 250mM, the tissue was transferred in PBS for homogenization with a 2 ml glass dounce and the cell suspension was filtered with a 40μm cell strainer (Falcon). Cells were washed twice in buffer A (10mM Tris-HCl pH 8.0, 10mM KCl, 0.25% Triton X-100, 1mM EDTA, 0.5mM EGTA, 1x cOmplete protease inhibitor cocktail EDTA-free (Roche)) for 5 min on ice. After spinning, cells were washed once in buffer B (10mM Tris-HCl pH 8.0, 200mM NaCl, 1mM EDTA, 0.5mM EGTA, 1x cOmplete) for 10 min on ice. After incubation and spinning, cells were lysed in 1% SDS, 10mM EDTA, 50mM Tris-HCl pH 8.0, 1x cOmplete for 30 min at 4°C on a rotating wheel. Sonication was performed on a Bioruptor UCD-300 sonicator (Diagenode) with the following conditions: 30 sec ON, 30 sec OFF, High power, 4x10 Cycles. Chromatin was diluted ten folds in IP dilution buffer: 5mM Tris-HCl pH 8.0, 140mM NaCl, 0.5% Triton X-100, 0.05% sodium deoxycholate, 0.5mM EGTA and one ml of chromatin was pre-cleared with 20 μl of Dynabeads Protein A (Invitrogen) for 4 h at 4°C on a rotating wheel. In parallel, 20 μl of Dynabeads Protein A were incubated with 3 µg of the appropriate antibody for 5 h at 4°C on a rotating wheel. After incubation with antibodies, the beads were washed twice in PBS, 0.05% Tween 20, 0.1mM DTT for 5 min at 4°C on a rotating wheel, and then once in IP dilution buffer for 10 min at 4°C on a rotating wheel. After the pre-clear, one ml of chromatin was incubated with the beads-antibody complex overnight at 4°C on a rotating wheel. IPs were washed once on each of the following buffers: Wash 1 (10mM Tris-HCl pH 8, 150mM KCl, 0.50% NP40, 1 mM EDTA), Wash 2 (10mM Tris-HCl pH 8, 100mM NaCl, 0.10% sodium deoxycholate, 0.50% Triton X-100), Wash 3a (10mM Tris-HCl pH 8, 400mM NaCl, 0,10% sodium deoxycholate, 0.50% Triton X-100), Wash 3b (10mM Tris-HCl pH 8, 500mM NaCl, 0,10% sodium deoxycholate, 0.50% Triton X-100), Wash 4 (10mM Tris-HCl pH 8, 250mM LiCl, 0,50% sodium deoxycholate, 0.50% NP40, 1 mM EDTA), TE (10mM Tris-HCl pH 8, 1mM EDTA). DNA was eluted (and cross-linking reversed) by incubation at 65°C overnight in 50mM Tris-HCl pH 8, 1% SDS, 1mM EDTA. After treatments with RNAse A and proteinase K, DNA was purified with MinElute columns (Qiagen, 28004).

DMC1 ChIP experiments were executed following the methods previously outlined in (17) and (18). The chromatin was prepared from 150-200 mg of testes. Frozen tissue was fixated in PBS, 1% formaldehyde for 10 min at room temperature. Glycine was added to a final concentration of 125mM to quench unreacted PFA and the tissue was passed through a 7 ml dounce for homogenization. Single cell suspension was filtered passing it through a 70μm cell strainer (Falcon). Cells were washed once in PBS and after centrifugation, cells were resuspended in buffer L1 (10mM Tris-HCl pH 8.0, 10mM EDTA, 0.5mM EGTA, 0.25% Triton X-100, 1x cOmplete protease inhibitor cocktail EDTA-free (Roche)). After spinning, cells were resuspended in buffer L2 (10mM Tris-HCl pH 8.0, 200mM NaCl, 1mM EDTA, 0.5mM EGTA, 1x cOmplete). After centrifugation cells were resuspended in buffer L3 (1% SDS, 10mM EDTA, 50mM Tris-HCl pH 8.0, 1x cOmplete) and immediately sonicated on a Bioruptor UCD-300 sonicator with the following conditions: 15 sec ON, 45 sec OFF, High power, 15 Cycles. Sheared chromatin was diluted by adding one volume of ChIP dilution buffer: 16.7mM Tris-HCl pH 8.0, 167mM NaCl, 1.1% Triton X-100, 0.01% SDS, 1.2mM EDTA. The chromatin was dialyzed for 5 hours at 4°C using constant rotation prior incubation with antibodies. The chromatin was incubated overnight with 24μg of antibody at 4°C on a rotating wheel. 150 μl of Dynabeads Protein A were incubated with 3 ml of ChIP for 2 h at 4°C on a rotating wheel. IPs were washed once on each of the following buffers: Wash 1 (20mM Tris-HCl pH 8, 150mM NaCl, 1% Triton X-100, 0.1% SDS, 2mM EDTA), Wash 2 (20mM Tris-HCl pH 8, 500mM NaCl, 1% Triton X-100, 0.1% SDS, 2mM EDTA), Wash 3 (10mM Tris-HCl pH 8, 250mM LiCl, 1% Deoxycholic acid, 1% IGEPAL, 1mM EDTA). Two final washes in TE (10mM Tris-HCl pH 8, 1mM EDTA) and DNA elution by incubation at 65°C for 30 min in 100mM NaHCO3, 1% SDS. Beads were discarded and cross-linking was reversed by incubating at 65°C overnight in 200mM NaCl,100mM NaHCO3, 1% SDS. After treatment with proteinase K, DNA was purified with MinElute columns (Qiagen, 28004).

### Library preparation and sequencing

For histone modifications ChIP, standard library construction was performed according to the NEBNext Ultra II protocol for Illumina (NEB, E7645S-E7103S), with minor adjustments. All clean-up steps in the protocol were achieved though MinElute columns (Qiagen, 28004). For size selection, we performed agarose gel electrophoresis and DNA fragments within the range of 200 to 400 bp were excised from the gel and the DNA purified using MinElute columns (Qiagen, 28604). For DMC1 ChIP, library construction was performed following the Illumina TruSeq protocol (Illumina, IP-202-9001DOC), with the introduction of an extra step of kinetic enrichment as previously described (17, 18). The NovaSeq6000 platform (Illumina) with S4 flow cells was employed for sequencing the libraries, and all the sequencing procedures were carried out at Novogene Europe (Cambridge, United Kingdom).

### Computational Data Analysis

For all ChIP-seq experiments, paired-end reads were mapped to the USDA_OmykA_1.1 assembly. We analyzed histone modifications through the nf-core/chipseq v1.2.1 pipeline (19), accessible at https://github.com/nf-core/chipseq. The pipeline was executed using *Nextflow v20.10.0*. The sequencing reads were aligned to the reference genome with *BWA v0.7.17-r1188*. The pipeline filtered by default the mapped reads to retain only the non-duplicated and high-quality uniquely mapping reads. For both histone modifications, we normalized the signal based on the total read coverage and by subtracting the input using *bamCompare* function (*normalizeUsingRPKM*) of *deepTools* (20). Peak calling was performed using *MACS2 v2.2.7.1* (parameters: *--pvalue=1e-5 --narrow_peak --read_length=150 --macs_gsize 2.07E9*) on both replicates, and an input for each sample was provided. The peaks found within 1000 bp of one another were combined using *merge* function of *Bedtools* (21), and the mean of their scores was reported (parameters: -d 1000 -c 5 -o mean). We used the *computeMatrix* function of *deepTools* with *scale-regions* mode. We assessed the enrichment of the histone modifications at DMC1 peaks by using *deepTools computeMatrix*, *plotProfile* and *plotHeatmap* to compute and display average profiles and heatmaps (**S8** **Fig**) (parameters*: reference-point --referencePoint center -b 5000 -a 5000 --skipZeros --missingDataAsZero --scale 1*). Using the same parameters, we assessed the enrichment of H3K36me3 signal at H3K4me3 peaks in brain using the bed files produced in the context of the Aqua-FAANG project (https://www.aqua-faang.eu/). The peaks were processed by Bedtools *merge* and Bedtools *interstect* to allow meaningful comparison with our H3K4me3 data. Details regarding the overlapping strategies applied can be found in the "*Determination of Overlapping Peaks*" section. Subsequently, the bed files were subjected to analysis using *deeptools computeMatrix* and *deeptools plotHeatmap* to compute and visualize heatmaps.

The analysis of DMC1 ChIP-seq was performed as described (18), with some implementations (22). We used the *hotSSDS* pipeline (version 1.0), which is an adaptation of the original *SSDS* pipeline (https://github.com/kevbrick/SSDSnextflowPipeline) and the *SSDS call peaks* pipeline (https://github.com/kevbrick/callSSDSpeaks). The *hotSSDS* pipeline was customized and modified by Pauline Auffret (IFREMER) and Julie Clément (IHPE), and their version can be found at https://github.com/jajclement/hotSSDS. The pipeline was executed using *Nextflow 21.10.0*. Reads were mapped using the modified BWA algorithm (*BWA Right Align*), initially developed by (18) to align and recover ssDNA fragments, then improved (17). Aligned reads were filtered in order to retain only the non-duplicated and high-quality uniquely mapped reads. The signal was normalized by the library size, and then by the total type 1 ssDNA fragments. The peak calling was performed using *MACS2 v2.2.7.1* with relaxed conditions (parameters: *--pvalue=1e-2 --bw=1000 --nomodel --slocal=5000 --extsize=800*) on each of the two replicates, while an input control was provided. Peak calling was additionally performed on the pooled dataset, and on pseudo-replicates that were artificially generated by randomly subsampling half the reads twice from each replicate (self pseudo-reps), or from the pooled dataset (pooled pseudo-reps). Then Irreproducible Discovery Rate (*IDR*) analysis was performed as described (23) to allow the identification of highly reproducible peaks between the true replicates (with a threshold of 0.05), between the pooled pseudo-replicates (with a threshold of 0.01), or between self pseudo-replicates (with a threshold of 0.05), to assess the self-reproducibility. Final peak sets were created by picking up the top peaks from the dataset of origin, meaning all peaks having an *IDR* threshold inferior to 0.01 for pooled dataset, as recommended by the authors. The IDR peaks from the pooled dataset were processed using the script *normalizeStrengthByAdjacentRegions.pl* developed by (17). The script considers the distribution of ssDNA fragments to recenter the peaks by the median of the forward/reverse fragments distribution and outputs recentered and normalized peaks.

The final peaks were used to check the distribution of ssDNA type 1 signal at the DSB hotspots using *deepTools computeMatrix* and *deepTools plotHeatmap* (**S6 Fig**) (parameters: *reference-point --referencePoint center -b 2500 -a 2500 --skipZeros --missingDataAsZero --scale 1 --maxThreshold 15* for TAC-1 and *--maxThreshold 10* for TAC-3). Additionally, we computed the relative position of each DSB along its respective chromosome by utilizing the R package *dplyr* (**S7** **Fig**). As replicates were validated to be reproducible, we merged the normalized read distribution from each replicate (*i.e.* 2 bigwig files per sample) for further analyses by the *UCSC Genome Browser*’s utility.

### Correlation between read enrichments of histone modifications

To assess the correlation in signal distribution from histone ChIP between samples, we used the *multiBigwigSummary* function from *deepTools*. This tool computes summary statistics for multiple bigwig files, more specifically on signal intensity measured across specified genomic intervals. We thus calculated the correlation between H3K4me3 signal and H3K36me3 signal at RT-52 DMC1 peaks, the H3K4me3 signal in TAC-1 and TAC-3 samples at the RT-52 DSB hotspots and the correlation between H3K36me3 signal in TAC-1 and TAC-3 samples at the same regions (using read distribution bigwig files obtained for merged replicates). The statistical significance of the results was evaluated using the chi-square test, and scatterplots were generated using GraphPad Prism software (**S9 Fig**).

### Analysis of DMC1 ChIP-seq signal at genomic features

To investigate the DSB activity at various genomic features, we used the distribution of DMC1-SSDS read enrichment as a metric. The genomic features of interest were initially identified on the assembly Omyk_1.0, as described in section “*Recombination at genomic features*” below. We used *NCBI remap* tool to perform the liftover of the genomic regions on the assembly USDA_OmykA_1.1 (parameters: *--mode asm-asm --from GCF_002163495.1 --dest GCF_013265735.2 --annotation <bed file> --annot_out <bed file>*). Subsequently, we applied the functions *distanceToNearest* and *subsetByOverlaps* from the R package *GenomicRanges* (24), as detailed in the aforementioned section. The bedgraph files obtained from the DMC1-SSDS corresponding to read distribution for merged replicates were used to calculate the averaged read distribution within each genomic feature (**S6 Fig**).

### Determination of overlapping peaks

We used the *intersect* function of Bedtools to assess the overlaps between different features by the default options. For overlaps purposes, the central 400 bp of each DSB hotspot peak was used, as previously described (25) (**S6 Fig**). When comparing DMC1 peaks with H3K4me3 and H3K36me3, we used the central 400 bp of the hotspots and the full-length positions of the histone modifications. To determine the intersection with the transcription start/end sites, we defined the TSS and TES regions as, respectively the 5’ and the 3’ end of the transcripts from the RefSeq annotated genes ±1 kb (**S3 Table**). To identify the overlapping intervals between replicates and between samples when the IDR methodology was not applied (*i.e.* for H3K4me3 and H3K36me3 peaks), we used the tool with more stringent criteria (parameters: *-f 0.25 -r -u*) to retrieve reproducible peaks.

### Analysis of DSB hotspot annotation

To annotate the DSBs in relation to the RefSeq genome annotation, we employed the *annotatePeaks.pl* function from the *HOMER* suite, utilizing the *-gtf* option. For visualizing the results, we utilized a script adapted from the *hotSSDS-extra* pipeline (22) for further processing the results generated by the *hotSSDS* pipeline. This pipeline is designed to automate the computation and visualization of general statistical data related to the SSDS signal, and can be found at (https://github.com/jajclement/hotSSDS-extra). Specifically, we used the script named *plot_homer_annotatepeaks.r*, which was integrated in the *nf-core chipseq* *v1.2.1* pipeline, as described (19). This script takes the results of peak annotation to generate visual representations (**Fig 3D**).

## Reconstruction of population-based recombination landscapes in three salmonids (indirect estimation of recombination rates)

### Whole genome data

We collected high coverage whole-genome resequencing data from five natural populations of three salmon species from the SRA database to reconstruct population-based recombination landscapes, with approximately 20 samples per population as recommended (26). We retrieved 20 samples from the Southern British Columbia population of the coho salmon, *O. kisutch* (27), 22 rainbow trout (*O. mykiss)* samples from North East America (28), and 60 genomes from three populations of *S. salar* belonging the two major lineages in North America and Europe (29): 20 from the Gaspesie Peninsula in Canada (referred to as the GP population), 20 from the North Sea (NS population) and 20 from the Barents Sea in Norway (BS population). Sample accession number and location is detailed in **S10** **Table** and **S27** **Fig**.

### Variant Calling

Variant calling for *O. kisutch* was performed by (27). We followed the same methodology for variant calling and genotyping in *O. mykiss* and *S. salar*, using the GATK best-practice pipeline (> v3.8-0, see detailed versions of the programs used in **S11 Table**) (30, 31). First, we aligned individual paired-end reads against their reference genome (assembly and RefSeq accession number: *O. kisutch*: Okis_v1, GCF_002021735.1; *O. mykiss*: Omyk_1.0, GCF_002163495.1; *S. salar*: Ssal_v3.1, GCF_905237065.1) using bwa-mem (v0.7.17) (32), yielding an average depth coverage per sample of 29.54x, 24.87x and 9.97x for *O. kisutch*, *O. mykiss* and *S. salar*, respectively (**S4** **Table**). Among the multiple primary alignments generated per query sequences, we flagged shorter split hits as secondary with the *-M* option for Picard compatibility. Possible PCR duplicates were then marked with the Picard MarkDuplicates program (> v2.18.29, validation stringency parameter was set to lenient). All reads were assigned to a new read-group ID using Picard AddOrReplaceReadGroups. We called variants for each individual of *O. mykiss* and *S. salar* using HaplotypeCaller with allele-specific annotations, generating GVCF files (options *-G* *StandardAnnotation, -G AS_StandardAnnotation, -G StandardHCAnnotation*). After creating a GenomicsDB workspace (GenomicsDBImport), joint genotyping (GenotypeGVCFs, default settings) was completed by pooling all individuals for each species. A total of 9,590,270, 39,601,311 and 27,061,466 SNPs were called for *O. kisutch* (27), *O. mykiss* and *S. salar*, respectively.

Following genotyping, we removed variants within 5 bp of an indel with Bcftools filter (v 1.9; *-g 5*). We filtered low-quality SNPs with Vcftools (> v 0.1.16) (33). We excluded indels (*--remove-indel*), variants with more than 2 alleles (*--max-alleles 2, --min-alleles 2*), genotypes with quality scores < 20 (--minGP 20), and SNPs with >10% of missing values (*--max-missing 0.9*). For *S. salar*, the missingness threshold was set to 50% because of a higher rate of missing genotypes in this dataset. To control for poorly sequenced regions or duplicated loci, we kept only sites with a mean depth coverage falling within the 5–95% quantiles of the species’ distribution (*O. kisutch*: 13x-33x; *O. mykiss*: 7x-23x; *S. salar*: 2x-9x). We applied a Hardy-Weinberg test with a *p*-value threshold of 0.01 (*--hwe 0.01*), expecting to filter excess of heterozygosity due to hidden paralogy in particular within residual tetrasomic regions. We removed singletons by applying a MAC (minor allele count) filter with Vcftools (*--mac 2*). For *S. salar*, the Hardy-Weinberg and MAC filters were applied separately for each of the three populations. The filtering steps retrieved 7,205,269, 16,079,097 and 5,575,430 SNPs for *O. kisutch*, *O. mykiss* and *S. salar*, respectively (**S4** **Table**).

### Variant phasing and orientation

We used the read-based phasing approach in WhatsHap (> v0.18) (34) to identify phase blocks from paired-end reads overlapping neighboring individual heterozygous positions. Prephasing statistics calculated using Whatshap *stats* showed that 73.45% and 76.98% of the variants were physically phased for *O. kisutch* and *O. mykiss*. Due to the lower SNPs density and higher genotype missingness, the *S. salar* dataset was prephased for only 7.32% of its variants. Prephased blocks were then phased chromosome-wide using the statistical phasing approach in SHAPEIT4 (> v4.2.1) (35), default settings: phase-set error rate *--use-PS 0.0001* and MCMC iteration scheme *5b,1p,1b,1p,1b,1p,5m*), assuming a constant recombination rate of 3 cM/Mb (representative of average recombination rates in teleosts (36)) and using the effective population size estimated from the nucleotide diversity of each chromosome, which was calculated in 100 kb windows using Vcftools.

Ancestral allelic states were inferred for the set of variants of each species. Three outgroups were used for each species: *O. kisutch* variants were orientated using the reference genome of *O. mykiss*, the sockeye salmon *Oncorhynchus nerka* (assembly Oner_1.1, RefSeq accession number GCF_006149115.1) and the chinook salmon *Oncorhynchus tshawytscha* (Otsh_v1.0, GCF_002872995.1), *O. mykiss* with *O. kisutch*, *O. nerka* and *O. tschawytscha*, and *S. salar* with *O. mykiss*, the brown trout *Salmo trutta* (fSalTru1.2, GCA_901001165.2), and the arctic char *Salvelinus alpinus* (ASM291031v2, GCF_002910315.1). We retrieved 100bp-flanking sequences of each SNPs in the ingroup species with the *getfasta* function of Bedtools and used blastn to find their ortholog positions on the reference genome of each outgroup species, retaining only the best hits (*-outfmt 6, -max_target_seqs 1, -max_hsps 1*). The corresponding SNPs positions in the query sequences were retrieved using a custom rust script. Ancestral state probabilities were then inferred with the maximum likelihood method implemented in est-sfs (v2.04) (37), using the ingroup allele frequencies and the allelic states of the outgroups. Phylogenetic relationship between ingroup and outgroup species were taken into account (38) and the mean sequence identity scores computed with blastn: (((*O. kisutch, O. tshawytscha*), *O. nerka*), *O. mykiss*); (((*O. mykiss, O. tshawytscha*), *O. nerka*), *O. kisutch*); (((*S. salar, S. trutta*), *S. alpinus*), *O. mykiss*) (see **S13** **Table**). Est-sfs was run using the Kimura-2-parameter substitution model.

### Estimation of linkage disequilibrium-based recombination rates

We estimated population recombination rates *⍴* (*⍴*=4*N*_e_*r,* with *N*_e_ the effective population size and *r* the recombination rate in M/bp) with LDhelmet (v1.19) (39) for the five populations of *O. kisutch*, *O. mykiss*, and S. salar (*i.e.* GP, BS and NS). LDhemet infers the *⍴* parameter between pairs of SNPs with a reversible-jump Markov Chain Monte Carlo algorithm. SNPs data were converted into fasta sequences for each individual haplotype using the *vcf2fasta* function of vcflib (<https://github.com/vcflib/vcflib>), and into the position and SNPs input format with the *--ldhelmet* option of VCFtools. Ancestral allelic states were provided using the probability (P) of the major allele being ancestral computed by est-sfs, and 1-P to the minor allele. LDhelmet was run five times independently for each population. For each chromosome, the haplotype configuration files were created with the find_conf function using the recommended window size of 50 SNPs. The likelihood look-up tables were created once for the five runs with the table_gen function using the recommended grid for the population recombination rate (*ρ*/pb) (*i.e. ρ* from 0 to 10 by increments of 0.1, then from 10 to 100 by increments of 1), and with the Watterson *θ = 4N*_e_*μ* parameter of the corresponding chromosome computed in 100 kb windows with Vcftools and using *μ*=10^-8^. The Padé files were created using 11 Padé coefficients as recommended. The Monte Carlo Markov chain was run for 1 million iterations with a burn-in period of 100,000 and a window size of 50 SNPs, using a block penalty (BP) of 5. A transition matrix, computed following (39) was used:


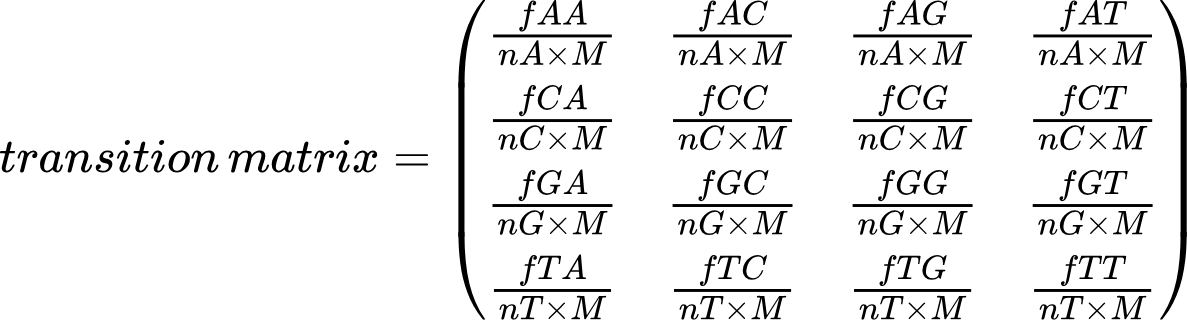


with $fAA$, …, the number of substitution from A to A, …, computed from the polarized SNPs data; $nA$, …, the number of nucleotide A in the genome, $M$a standardization factor corresponding to the maximum value between $(fAC+fAG+fAT)/nA$, $(fCA+fCG+fCT)/nC$, $(fGA+fGC+fGT)/nG$, and $(fTA+fTC+fTG)/nT$.

The convergence of the five independent runs of LDhelmet was estimated with Spearman’s rank correlation test. The five runs were averaged together and smoothed within 2 kb, 100 kb and 1 Mb windows using custom python scripts.

We reconstructed the fine-scale recombination landscape of the European sea bass (*Dicentrarchus labrax*) to compare recombination properties in salmonids with those of a species lacking a complete *Prdm9* gene due to loss of the KRAB domain. Whole-genome haplotype data obtained via phasing-by-transmission and statistical phasing (40) was used to infer recombination in the Atlantic sea bass population with LDhelmet with a similar strategy, using the seabass_V1.0 genome assembly (GenBank accession number GCA_000689215.1) (**S4 and S11** **Table**).

### Identification of LD-based recombination hotspots

We identified recombination hotspots from both the raw recombination map inferred by LDhelmet (referred to as raw hotspots) and the 2 kb smoothed recombination map (referred to as 2 kb hotspots) using a sliding window approach. Hotspots were defined as intervals between two consecutive SNPs or windows of 2 kb with a relative recombination rate 5-fold or higher than the mean recombination rate in the 50 kb flanking regions. When consecutive 2 kb windows exceeded the threshold, only the one with the highest rate was retained.

### Comparison between DSB sites and LD-based hotspots

DSBs sites mapped with DMC1-SSDS for the pooled samples RT-52, TAC-1 and TAC-3 were compared to the LD-based recombination hotspots retrieved from the recombination landscapes of *O. mykiss*. To allow the comparison, we converted the genomic positions of the DSB hotspots mapped on the OmykA_1.1 assembly to Omyk_1.0 coordinates on which we built the LD map using the Remap program from NCBI. We compared the locations of LD-hotspots with DSB hotspots using Bedtools *intersect*.

### Population recombination rate variation at genomic features

We investigated how LD-based recombination rates and hotspots distribute with respect to genomic features. We first retrieved the positions of genes, exons and introns from genome annotations in each species. De novo identification of TEs families in the *O. kisutch*, *O. mykiss* and *S. salar* reference genomes was performed using RepeatModeler (v2.0.3) (41). We first used the BuildDatabase command without options to build a database from the reference genome. We ran RepeatModeler on the database generated with the LTR discovery pipeline (option -LTRStruct). We then soft-masked TEs with RepeatMasker (version 4.1.3, http://www.repeatmasker.org/, options -xsmall, -nolow) using the library of consensus sequences generated by RepeatModeler. An annotation file of the TEs and low complexity DNA sequences was generated. We retrieved the genomic location of introns from gene and exon locations, and intergenic regions from gene and TEs locations using Bedtools *subtract*. Transcription start and end sites (TSSs and TESs) were defined as the first and last positions of the genes, respectively. We predicted CpG islands (CGIs) of each reference genome with EMBOSS *cpgplot* (v6.6.0) (42), using a window size of 500 bp to calculate the percentage of GC content and the observed frequency of GCs (*-window 500*), with the minimum length of a CGI set to 250 bp (*-minlen 250*). It should be noted that the criteria that are classically used to predict CGIs in mammals or birds (CpG observed/expected ratio > 0.6, GC-content > 50%) are not appropriate for teleost fish, whose CGIs are CpG-rich but have a low GC-content (43, 44). We therefore predicted CGIs solely based on their CpG content (minimum average CpGoe < 0.6, -minoe 0.6), without any constraint on their GC-content (-minpc 0). We checked that these criteria efficiently predict TSS-associated CGIs, using whole genome DNA methylation and H3K4me3 data from rainbow trout and coho salmon (see **S1 Analysis**). TSS overlapping or not CGIs were then determined using the *subsetByOverlaps* function of the R package GenomicRanges (24).

We assessed population recombination rate (2 kb scale) variation according to the distance to the nearest TSS by calculating the distances of the genomic windows of the 2 kb smoothed map to the nearest TSS using the *distanceToNearest* function. The averaged population recombination rates in genes, exons, introns, TEs, TSS overlapping or not a CGI, TES, CGIs were estimated using *subsetByOverlaps*. We compared levels of recombination rates at genomic features of the five salmonid populations to the sea bass.

We finally investigated the effect of SNP density, GC content and TEs density on population recombination rate variation and the presence of recombination hotspots. We retrieved SNP count in 100 kb and 2 kb sliding windows on the filtered SNP data containing singletons. We determined the number TEs in each window of the 100 kb smoothed maps using Bedtools *intersect* to calculate TE density. GC-content was also calculated in the windows of the 100 kb and 2 kb smoothed maps as the ratio of the sum of C and G nucleotides to the sum of the four nucleotides A, C, G, T in each window. We calculated Spearman's rank correlation between SNP density, GC content and TE density with recombination rates at the 100 kb scale. The distance of the 2 kb genomic windows to the nearest hotspots was calculated using Bedtools *closest* to assess SNP density and GC-content around recombination hotspots.

### Comparison of LD-based landscapes between populations and species

We assessed the correlation between the 100 kb smoothed recombination maps of each of the three *S. salar* populations using a Spearman's rank test. We identified shared hotspots as overlapping 2 kb hotspots using Betdtools *intersect*. We used random permutations to calculate the expected amount of hotspot overlap between the three pairs of populations. Random spots totalling the number of 2 kb hotspots were drawn one hundred times from the genome of each population using Bedtools *shuffle*. To control for diversity level, extreme ⍴ values and genome gaps, a genome mask has first been applied to discard the regions with a nucleotide diversity lower and higher than the 2.5 and 97.5th quantiles, the 0.1% highest recombination rate values, the 10% larger gap sizes, and genuine hotspots. Each of the random spot sets was then compared to those of the other two populations to calculate the expected average overlap between the populations

To compare the recombination hotspots of *O. kisutch* and *O. mykiss*, whose recombination landscapes were built using their own reference genome, we used a reciprocal blast approach to retrieve the corresponding coordinates in the genome of the other species. We retrieved the fasta sequences of the *O. kisutch* 2 kb hotspots from the reference genome using Bedtools *getfasta,* and blasted with blastn on the *O. mykiss* reference genome, keeping only the best matches with *-max_targets_seq 1* and *-max_hsps 1* to retrieve the corresponding position in the *O. mykiss* genome. We retrieved the fasta sequences of the resulting blast hits in the *O. mykiss* reference genome with Bedtools *getfasta* and reciprocally blasted it on the *O. kisutch* reference genome with blastn, again retaining only the best match. This approach ensured that only reciprocal blasted positions were retained and gave the coordinates of the hotspots of *O. kisutch* on the genome of *O. mykiss*. We determined the common hotspots between *O. kisutch* (with the coordinates in the *O. mykiss* genome) and *O. mykiss* with Bedtools *intersect*. We also performed a similar reciprocal blast of *O. mykiss* 2 kb hotspots to obtain their coordinates in the *O. kisutch* genome, and determined their common hotspots. We then used the number of overlapping hotspots obtained with the *O. kisutch* coordinates, the results being similar with the coordinates in the *O. mykiss* reference genome. We also performed random permutation to obtain the distribution of shared hotspots between the two species expected by chance.

### Controlling for a possible effect of limited sequencing coverage

Limited sequencing coverage may affect the inference of population recombination rates, because it can lead to miss heterozygous positions, thus increasing noise in the LD map. To control for the possible impact of coverage in our dataset, particularly for *S. salar*, which has an average sequencing depth of only 10x (**S4 Table**), we repeated our analyses by dividing genome regions into two groups: one with the lowest and one with the highest mean sequencing coverage.

In all species, the hotspot density and the distribution of recombination rates (genome-wide, or at the vicinity of gene features), was very similar in regions with highest or lowest coverage (**S29 Fig**). Some weak differences were observed, but in all cases, with negligible size effect (Cohen's D < 2; (45)).

Furthermore, the degree of overlap between LD-based recombination hotspots from populations remained broadly similar when comparing hotspots in the lowest and highest coverage regions (**S30 Fig**). When comparing the BS and NS populations of Atlantic salmon, 25.8% of hotspots from the lowest coverage regions and 28% of those from the highest coverage regions were shared (**S30 Fig**), whereas in the original analysis we obtained 26.3% of shared hotspots (**Fig 5C**).

These analyses indicate that low coverage regions do not significantly affect estimated intragenomic variation in recombination rates in our study.

## Identification of DNA motifs at hotspots and motif erosion

***DNA motifs enriched in rainbow trout DSB hotspots***

In the rainbow trout, we used the MEME Suite (46) to detect motifs associated with DSB hotspots, focusing on the RT-52 dataset due to its high number of DSB hotspots (DMC1 peaks). Two distinct subsets of allele-specific hotspots were defined by the intersection of the DMC1 peaks with histone modifications via Bedtools *intersect*. We then retrieved the fasta sequences using Bedtools *getfasta*.

- Allele 1 set: RT-52 DMC1 peaks (center ± 200 bp) overlapping H3K4me3 and H3K36me3 peaks from TAC-1 (N = 300)

- Allele 2 set: RT-52 DMC1 peaks (center ± 200 bp) overlapping H3K4me3 and H3K36me3 peaks from TAC-3 (N = 254).

DSB hotspots from allele 1 set showed no overlap with hotspots from TAC-3, and similarly, we did not report any overlap between allele 3 set and TAC-1 hotspots.

We used MEME-ChIP (v5.5.4) (47) to screen for motifs the two allele-specific set of peaks, with the following settings:

- Discovery Mode: Classic

- Background: 2-order model from the input sequences

- Motif Width: 8-20 nt for MEME and STREME (inclusive)

- MEME Site Distribution: zero or one occurrence per sequence

- MEME Motif Count: 5 motifs

- STREME p-value Threshold: p-value ≤ 0.05

- STREME Site Positional Distribution Plots: Sequences are aligned on their centers

- CentriMo Match Score: match score ≥ 5

- CentriMo E-value Threshold: E-value ≤ 10

- CentriMo Local: central enriched regions.

Two motifs, one per allele-specific set, having high significance as well as central enrichment were further processed (**Fig 3C**). To investigate specific enrichment of the motifs at DSB and LD-based hotspots (center ± 1 kb), we run FIMO (48) with a p-value cutoff of 1.0E-5 and the background model specified in the motif input. For proper comparison, fasta sequences for both DSB and LD hotspots have been collected into the assembly USDA_OmykA_1.1 using Bedtools *getFasta*, with a prior liftOver step for LD-based hotspot regions (see section “*Analysis of DMC1 ChIP-seq signal at genomic features”*).

The two sets of regions used to perform the motif discovery analysis were included as positive controls, one additional set of control sequences (*i.e.* non-hotspot regions) was generated to match the GC-content (allele 1 set: 44.9%, allele 2 set: 44.3%, control: 43.8%) and the distance to telomeres of the positive control sets. Of note, the control set (N = 5000) showed some overlap with the DSB hotspots (TAC-1: 0.24%, TAC-3: 0.02%, RT-52: 0.52%) and the LD-based hotspots (2.74%), but not with the allele-specific sets. All sets of sequences screened for the two motifs discovered by MEME-ChIP were 2 kb long. We compared the frequency of sequences having at least one match to the motif for each set, to the frequency observed in the control sequences. The enrichment p-value relatively to the control was assessed by Fisher Exact test (**S22** **Fig**). We used CentriMo (49) to further evaluate the central enrichment of the two motifs at the control sequences (**Fig 3C**), at the DSBs hotspots and at LD-based hotspots (**S23** **Fig**), with default settings. The significance of the enrichment was automatically assessed by the tool and refers to the likelihood that the best match to the motif in a sequence occurs within the reported region.

### Motif erosion in European lineage of Atlantic salmon

We then tested whether the candidate motifs showed signs of erosion in the American compared to the European lineage and conversely by comparing the number of motifs present in available long-read genome assemblies from 5 North American Atlantic salmon genomes (GCA_021399835.1, GCA_931345555.1, GCA_931345325.1, GCA_931347555.1, GCA_923944775.2) and 7 European genomes (GCA_905237065.2, GCA_931346935.2, GCA_931345835.1, GCA_931347365.1, GCA_931345645.1, GCA_931345955.1, GCA_931345925.1). To take into account potential differences in the various assemblies, we aligned these 12 genomes with SibeliaZ (50), and retrieved the motif occurrences from the collinear blocks. We ran FIMO to count motif occurrence in the aligned fraction of each genomes, using a p-value cut-off of 1.0E-7. To assess the statistical significance of motif enrichment in a lineage, a null distribution was obtained by running FIMO on 100 random permutations of the candidate motif matrix.

**References**

1. Altschul SF, Gish W, Miller W, Myers EW, Lipman DJ. Basic local alignment search tool. J Mol Biol. 1990;215(3):403-10.

2. Ranwez V, Douzery EJP, Cambon C, Chantret N, Delsuc F. MACSE v2: Toolkit for the Alignment of Coding Sequences Accounting for Frameshifts and Stop Codons. Mol Biol Evol. 2018;35(10):2582-4.

3. Borowiec ML. AMAS: a fast tool for alignment manipulation and computing of summary statistics. PeerJ. 2016;4:e1660.

4. Christoffels A, Koh EG, Chia JM, Brenner S, Aparicio S, Venkatesh B. Fugu genome analysis provides evidence for a whole-genome duplication early during the evolution of ray-finned fishes. Mol Biol Evol. 2004;21(6):1146-51.

5. Macqueen DJ, Johnston IA. A well-constrained estimate for the timing of the salmonid whole genome duplication reveals major decoupling from species diversification. Proc Biol Sci. 2014;281(1778):20132881.

6. Vandepoele K, De Vos W, Taylor JS, Meyer A, Van de Peer Y. Major events in the genome evolution of vertebrates: paranome age and size differ considerably between ray-finned fishes and land vertebrates. Proc Natl Acad Sci U S A. 2004;101(6):1638-43.

7. Nguyen LT, Schmidt HA, von Haeseler A, Minh BQ. IQ-TREE: a fast and effective stochastic algorithm for estimating maximum-likelihood phylogenies. Mol Biol Evol. 2015;32(1):268-74.

8. Hayashi K, Yoshida K, Matsui Y. A histone H3 methyltransferase controls epigenetic events required for meiotic prophase. Nature. 2005;438(7066):374-8.

9. Birney E, Clamp M, Durbin R. GeneWise and Genomewise. Genome Res. 2004;14(5):988-95.

10. Baker Z, Schumer M, Haba Y, Bashkirova L, Holland C, Rosenthal GG, et al. Repeated losses of PRDM9-directed recombination despite the conservation of PRDM9 across vertebrates. eLife. 2017;6.

11. Cavassim MIA, Baker Z, Hoge C, Schierup MH, Schumer M, Przeworski M. PRDM9 losses in vertebrates are coupled to those of paralogs ZCWPW1 and ZCWPW2. Proc Natl Acad Sci U S A. 2022;119(9).

12. Billard R, Solari A, Escaffre AM. [Method for the quantitative analysis of spermatogenesis in teleost fish]. Ann Biol Anim Biochim Biophys. 1974;14(1):87-104.

13. Kanai K, Nunoya T, Shibuya K, Nakamura T, Tajima M. Variations in effectiveness of antigen retrieval pretreatments for diagnostic immunohistochemistry. Res Vet Sci. 1998;64(1):57-61.

14. Grey C, Baudat F, de Massy B. Genome-Wide Control of the Distribution of Meiotic Recombination. PLoS Biol. 2009;7(2):e35.

15. Diagouraga B, Clement JAJ, Duret L, Kadlec J, de Massy B, Baudat F. PRDM9 Methyltransferase Activity Is Essential for Meiotic DNA Double-Strand Break Formation at Its Binding Sites. Mol Cell. 2018;69(5):853-65 e6.

16. Tardat M, Brustel J, Kirsh O, Lefevbre C, Callanan M, Sardet C, et al. The histone H4 Lys 20 methyltransferase PR-Set7 regulates replication origins in mammalian cells. Nat Cell Biol. 2010;12(11):1086-93.

17. Brick K, Pratto F, Sun CY, Camerini-Otero RD, Petukhova G. Analysis of Meiotic Double-Strand Break Initiation in Mammals. Methods Enzymol. 2018;601:391-418.

18. Khil PP, Smagulova F, Brick KM, Camerini-Otero RD, Petukhova GV. Sensitive mapping of recombination hotspots using sequencing-based detection of ssDNA. Genome Res. 2012;22(5):957-65.

19. Ewels PA, Peltzer A, Fillinger S, Patel H, Alneberg J, Wilm A, et al. The nf-core framework for community-curated bioinformatics pipelines. Nat Biotechnol. 2020;38(3):276-8.

20. Ramírez F, Ryan DP, Grüning B, Bhardwaj V, Kilpert F, Richter AS, et al. deepTools2: a next generation web server for deep-sequencing data analysis. Nucleic Acids Res. 2016;44(W1):W160-5.

21. Quinlan AR, Hall IM. BEDTools: a flexible suite of utilities for comparing genomic features. Bioinformatics. 2010;26(6):841-2.

22. Auffret P, de Massy B, Clement JAJ. Mapping Meiotic DNA Breaks: Two Fully-Automated Pipelines to Analyze Single-Strand DNA Sequencing Data, hotSSDS and hotSSDS-extra. Methods Mol Biol. 2024;2770:227-61.

23. Li Q, Brown JB, Huang H, Bickel PJ. Measuring reproducibility of high-throughput experiments. The Annals of Applied Statistics. 2011;5(3):1752-79, 28.

24. Lawrence M, Huber W, Pagès H, Aboyoun P, Carlson M, Gentleman R, et al. Software for computing and annotating genomic ranges. PLoS Comput Biol. 2013;9(8):e1003118.

25. Brick K, Smagulova F, Khil P, Camerini-Otero RD, Petukhova GV. Genetic recombination is directed away from functional genomic elements in mice. Nature. 2012;485(7400):642-5.

26. Raynaud M, Gagnaire P-A, Galtier N. Performance and limitations of linkage-disequilibrium-based methods for inferring the genomic landscape of recombination and detecting hotspots: a simulation study. Peer Community Journal. 2023;3.

27. Rondeau EB, Christensen KA, Minkley DR, Leong JS, Chan MTT, Despins CA, et al. Population-size history inferences from the coho salmon (Oncorhynchus kisutch) genome. G3 (Bethesda). 2023;13(4).

28. Gao G, Nome T, Pearse DE, Moen T, Naish KA, Thorgaard GH, et al. A New Single Nucleotide Polymorphism Database for Rainbow Trout Generated Through Whole Genome Resequencing. Front Genet. 2018;9:147.

29. Bertolotti AC, Layer RM, Gundappa MK, Gallagher MD, Pehlivanoglu E, Nome T, et al. The structural variation landscape in 492 Atlantic salmon genomes. Nat Commun. 2020;11(1):5176.

30. McKenna A, Hanna M, Banks E, Sivachenko A, Cibulskis K, Kernytsky A, et al. The Genome Analysis Toolkit: a MapReduce framework for analyzing next-generation DNA sequencing data. Genome Res. 2010;20(9):1297-303.

31. Van der Auwera GA, Carneiro MO, Hartl C, Poplin R, Del Angel G, Levy-Moonshine A, et al. From FastQ data to high confidence variant calls: the Genome Analysis Toolkit best practices pipeline. Current protocols in bioinformatics. 2013;43(1110):11.0.1-.0.33.

32. Li H, Durbin R. Fast and accurate short read alignment with Burrows-Wheeler transform. Bioinformatics. 2009;25(14):1754-60.

33. Danecek P, Auton A, Abecasis G, Albers CA, Banks E, DePristo MA, et al. The variant call format and VCFtools. Bioinformatics. 2011;27(15):2156-8.

34. Martin M, Ebert P, Marschall T. Read-Based Phasing and Analysis of Phased Variants with WhatsHap. Methods Mol Biol. 2023;2590:127-38.

35. Delaneau O, Zagury JF, Robinson MR, Marchini JL, Dermitzakis ET. Accurate, scalable and integrative haplotype estimation. Nat Commun. 2019;10(1):5436.

36. Stapley J, Feulner PGD, Johnston SE, Santure AW, Smadja CM. Variation in recombination frequency and distribution across eukaryotes: patterns and processes. Philos Trans R Soc Lond B Biol Sci. 2017;372(1736).

37. Keightley PD, Jackson BC. Inferring the Probability of the Derived vs. the Ancestral Allelic State at a Polymorphic Site. Genetics. 2018;209(3):897-906.

38. Crespi BJ, Teo R. Comparative phylogenetic analysis of the evolution of semelparity and life history in salmonid fishes. Evolution. 2002;56(5):1008-20.

39. Chan AH, Jenkins PA, Song YS. Genome-Wide Fine-Scale Recombination Rate Variation in Drosophila melanogaster. PLoS Genet. 2012;8(12):e1003090.

40. Duranton M, Allal F, Valière S, Bouchez O, Bonhomme F, Gagnaire PA. The contribution of ancient admixture to reproductive isolation between European sea bass lineages. Evol Lett. 2020;4(3):226-42.

41. Flynn JM, Hubley R, Goubert C, Rosen J, Clark AG, Feschotte C, et al. RepeatModeler2 for automated genomic discovery of transposable element families. Proc Natl Acad Sci U S A. 2020;117(17):9451-7.

42. Larsen F, Gundersen G, Lopez R, Prydz H. CpG islands as gene markers in the human genome. Genomics. 1992;13(4):1095-107.

43. Cross S, Kovarik P, Schmidtke J, Bird A. Non-methylated islands in fish genomes are GC-poor. Nucleic Acids Res. 1991;19(7):1469-74.

44. Long HK, Sims D, Heger A, Blackledge NP, Kutter C, Wright ML, et al. Epigenetic conservation at gene regulatory elements revealed by non-methylated DNA profiling in seven vertebrates. eLife. 2013;2:e00348.

45. Cohen J. Statistical Power Analysis for the Behavioral Sciences (2nd Edition). Hillsdale, NJ: Lawrence Earlbaum Associates.; 1988.

46. Bailey TL, Johnson J, Grant CE, Noble WS. The MEME Suite. Nucleic Acids Res. 2015;43(W1):W39-49.

47. Machanick P, Bailey TL. MEME-ChIP: motif analysis of large DNA datasets. Bioinformatics. 2011;27(12):1696-7.

48. Grant CE, Bailey TL, Noble WS. FIMO: scanning for occurrences of a given motif. Bioinformatics. 2011;27(7):1017-8.

49. Bailey TL. STREME: accurate and versatile sequence motif discovery. Bioinformatics. 2021;37(18):2834-40.

50. Minkin I, Medvedev P. Scalable multiple whole-genome alignment and locally collinear block construction with SibeliaZ. Nat Commun. 2020;11(1):6327.
