# Supplementary material for: PRDM9 drives the location and rapid evolution of recombination hotspots in salmonid fish
Source: PLoS Biol. 2025 Jan 6;23(1):e3002950. doi: 10.1371/journal.pbio.3002950 (PMC11703093; doi:10.1371/journal.pbio.3002950)
Supplement: S1 Analysis — Fig A. Relationship between base composition and DNA methylation level in promoter regions of coho salmon (Oncorhynchus kisutch). Fig B. Relationship between DNA methylation level in the promoter regions of coho salmon and the presence of a nearby fpCGI. Fig C. Relationship between base composition and H3K4me3 in promoter regions of the rainbow trout (Oncorhynchus mykiss). Fig D. Relationship between H3K4me3 in the promoter regions of the rainbow trout and the presence of a nearby fpCGI. (DOCX) [file pbio.3002950.s002.docx]

**S1 Analysis: Prediction of CGI-associated TSSs in salmonids**

## Definition of CpG islands: constitutively hypomethylated genomic regions

Vertebrate genomes are heavily methylated, with generally more than 80% of CpG dinucleotides containing 5-methylcytosines (1-3). However, they also contain some short regions that escape DNA methylation and that are associated with H3K4me3 chromatin modification (trimethylation at lysine 4 of histone H3) (2, 4, 5). A large fraction of these non-methylated islands (NMIs) are associated to gene promoters (2). Typically, in human, mouse, chicken and zebrafish, 39% to 52% of NMIs overlap transcription start sites (TSS) of protein-coding genes, and reciprocally, 55% to 72% of protein-coding genes contain an NMI on their TSS (2). NMIs that are located in intergenic regions often show substantial variation in DNA methylation levels across tissues or during development (2). Conversely, NMIs that are associated to TSSs are maintained in the non-methylated state in most tissues, even in tissues where the corresponding gene shows no substantial transcription (2). Furthermore, the hypomethylation status of TSS-associated NMIs is often conserved across vertebrate species, which indicates that they are epigenetically stable not only across tissues but also through evolutionary time (2).

Methylated cytosines are hypermutable, which causes an overall depletion of CpG dinucleotides in vertebrate genomes, except in regions that escape DNA methylation (4, 6). This explains why NMIs generally display a much higher frequency of CpG dinucleotides compared to the rest of the genome (2). The term ‘CpG island’ (CGI) was coined to refer to these unmethylated CpG-rich loci (4). It should be noted that loci that are unmethylated in some tissues but methylated in the germline are not protected from CpG losses over evolutionary time. Thus, CGIs are expected to correspond to the subset of NMIs that are unmethylated in the germline, and this, stably over time. This explains why TSS-associated NMIs, which generally show a conserved and constitutive pattern of hypomethylation (2), are particularly CpG-rich (4).

## Bioinformatic prediction of CpG islands

In mammals and birds, CGIs are also characterized by a relatively high G+C content (4). There is now clear evidence that this feature results from the process of GC-biased gene conversion (gBGC) (7, 8). Indeed, in amniotes, substitution patterns in CGIs show an enrichment toward GC specifically in the lineages where they display an elevated meiotic recombination rate, not only in species that lack *Prdm9*, like dogs or birds (9-11), but also in species with an intact *Prdm9* (12, 13). Conversely, in species where recombination is suppressed in CGIs, such as humans or mice, their GC-content is decaying (8).

Grounded on these observations, originally made in mammals and birds, algorithms have been developed to predict CGIs based solely on their CpG and G+C content. We will hereafter use the term ‘*pCGI*’ to refer to these putative CGIs, predicted bioinformatically, without any direct measurement of their DNA methylation level. Classically, sequences of more than 200 bp, with a G+C content above 50%, and a ratio of observed over expected CpG content (*CpG_oe_*) above 0.6 are classified as *pCGI*s (14). Notably, the UCSC genome browser uses these criteria to annotate *pCGI*s in vertebrate genomes. These *pCGI*s are commonly used to investigate the genomic features associated to CGIs, in genomes for which DNA methylation or H3K4me3 data are not readily available.

It should be noted however that not all *pCGI*s correspond to *bona fide* CGIs. The overlap between experimentally characterized NMIs and *pCGI*s was analyzed in seven vertebrate species (2). In human, mouse and chicken, most *pCGI*s encompassed NMIs. Thus, in these species, *pCGI*s are indeed good predictors of CGIs. However, in other species (platypus, green anole lizard, xenopus and zebrafish) a majority of *pCGI*s do not correspond to NMIs (2). Furthermore, in zebrafish, NMIs have a high *CpG_oe_*, but a low G+C content (2). Interestingly, an early study, based on a limited number of genes, already had suggested that CGIs from carp and trout were not G+C-rich (15). These observations imply that the criteria that are classically used to annotate *pCGI*s in mammals or birds (*CpG_oe_* >0.6 and G+C>50%) are not appropriate to predict CGIs in teleost fish.

## Prediction of CGI-associated TSSs in salmonids

In our study, we wanted to identify the subset of TSSs that are associated to a CGI in salmonid genomes, to investigate whether they show an elevated recombination rate. Since DNA methylation and H3K4me3 data were available only for a fraction of the species that we wanted to analyze, we sought to predict CGI-associated TSSs based on their base composition. However, given the observations mentioned above, we first explored the criteria to be used to annotate *pCGI*s in salmonids.

For this, we used DNA methylation data available in the coho salmon (*Oncorhynchus kisutch*) (16) to investigate the relationship between the base composition of promoter regions and their DNA methylation level. Overall, the coho salmon genome is heavily methylated. For instance, in the liver sample that we analyzed, 65.2% of CpG sites show a high methylation level (>0.6), 8.1% have an intermediate methylation level ([0.2,0.6]) and 26.7% are hypomethylated (<0.2). We measured methylation levels in promoter regions (defined as the 500 bp upstream of the TSS) of protein-coding genes (N=27,832). Similar to other vertebrates, a large fraction of promoter regions (59.3%) are hypomethylated (**Fig A, panel A**). As expected, hypomethylated promoters have a higher *CpG_oe_* than highly methylated promoters (p-value Student’s t test < 1e-10) (**Fig A, panel C**). However, contrarily to amniotes, hypomethylated promoters are not G+C rich: they rather tend to have a lower G+C content than highly methylated promoters (p-value Student’s t test < 1e-10) (**Fig A, panel D**). Overall, only 1.1% of promoters (and 1.4% of hypomethylated promoters) match the criteria classically used to identify *pCGI*s (*CpG_oe_*>0.6 and G+C>50%, **Fig A, panel D**).

Thus, as previously reported for zebrafish (2), a high G+C content is not an appropriate criterion to predict CGIs in salmonids. We therefore tried to predict CGIs solely based on their *CpG_oe_*. We used the *cpgplot* software to identify DNA segments matching the following criteria:

- length > 250 bp
- *CpG_oe_* > 0.6

These DNA segments will hereafter be referred to as ‘fish putative CGIs’ (*fpCGIs*).


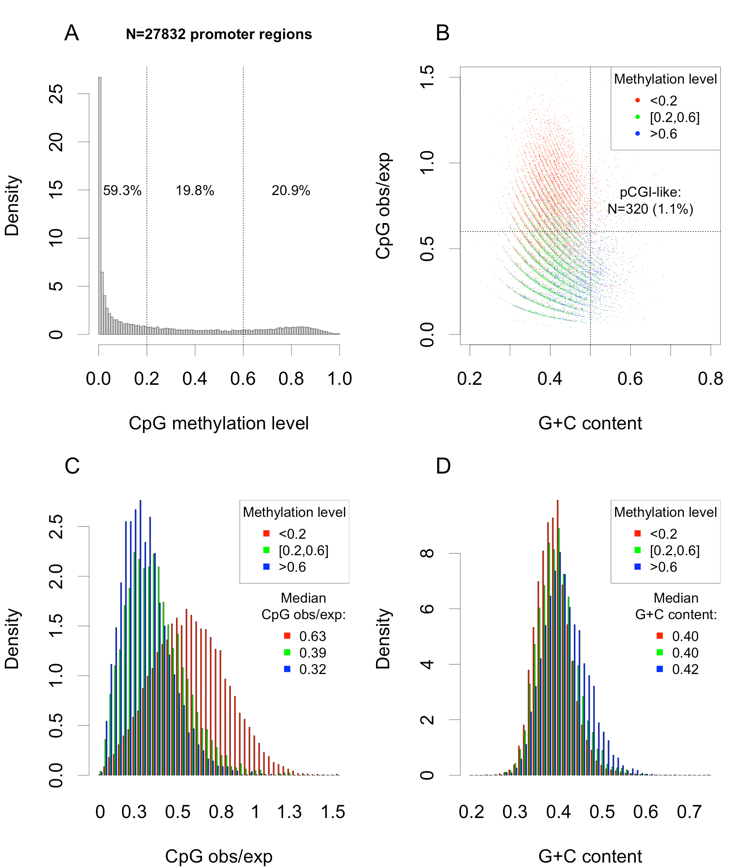


**Fig A: Relationship between base composition and DNA methylation level in promoter regions of coho salmon (*Oncorhynchus kisutch*).** We selected protein-coding genes (N=27,832) annotated in the coho salmon reference genome assembly and extracted their promoter region, defined as the 500 bp upstream of the TSS. For each promoter region, we quantified the average methylation level at CpG sites, using DNA methylation data from a liver sample (16). **A)** Distribution of CpG methylation level within promoter regions. **B)** CpG observed/expected ratio vs. G+C content of promoter regions. Promoters were classified according to their CpG methylation level (red: hypomethylated; blue: highly methylated; green: intermediate methylation level). The number and percentage of promoter regions matching the classical criteria for CGI annotation (CpG obs/exp>0.6 and G+C content >0.5) are indicated. **C)** Distribution of CpG observed/expected ratio of promoter regions, for different classes of CpG methylation level. **D)** Distribution of G+C content of promoter regions, for different classes of CpG methylation level.

We identified 667,422 *fpCGIs* in the coho salmon genome, and among the 27,832 annotated TSSs, 13,723 (49.3%) are located close to a *fpCGI* (<250 bp). The total number of *fpCGIs* largely exceeds the number of NMIs reported in vertebrate genomes (~11,000 to 41,000 NMIs) (2), which suggests that a large fraction of *fpCGIs* do not correspond to *bona fide* CGIs. However, we observed that the presence of a *fpCGI* is informative regarding the epigenetic status of promoter regions: indeed, among TSSs located close to a *fpCGI*, 81.9% are hypomethylated, whereas only 13.8% of TSSs located far from a *fpCGI* (>1000 bp) are hypomethylated (**Fig B**). This 5.9-fold enrichment indicates that the presence of a *fpCGI* close to the TSS is a very good predictor of the methylation state of the promoter region.


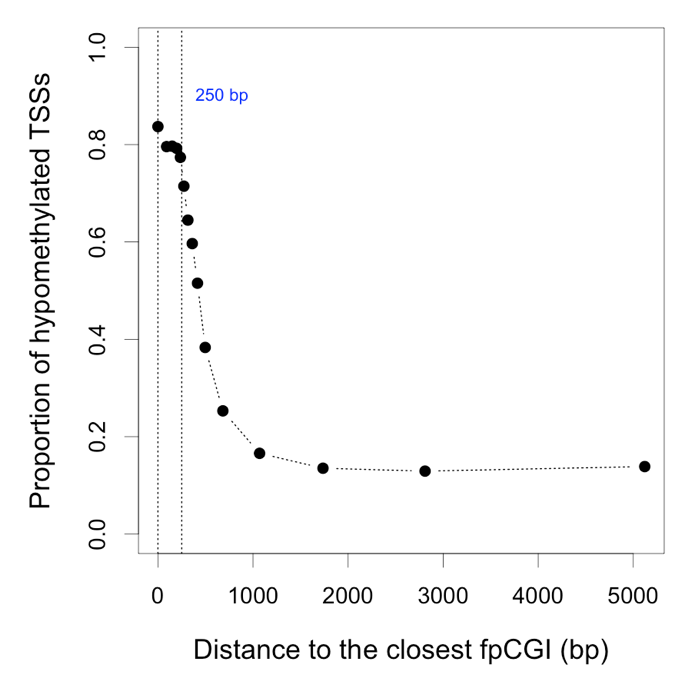


**Fig B: Relationship between DNA methylation level in the promoter regions of coho salmon and the presence of a nearby *fpCGI*.** We classified TSSs according to their distance to the nearest *fpCGI*, and computed the proportion of hypomethylated TSSs in each bin.

To determine whether the presence of a *fpCGI* also predicts histone epigenetic modifications of promoter regions, we analyzed H3K4me3 ChIPseq data from a rainbow trout (*Oncorhynchus mykiss*) brain sample (<https://data.faang.org/dataset/PRJEB57956>). We analyzed the distribution of H3K4me3 ChIPseq signal in promoter regions of protein-coding genes (N=40,786). Overall, 52.1% of promoter regions show a strong H3K4me3 ChIPseq signal (**Fig C, panel A**). These promoter regions with strong H3K4me3 display a high *CpG_oe_* and a relatively low G+C content (**Fig C, panels B-D**).


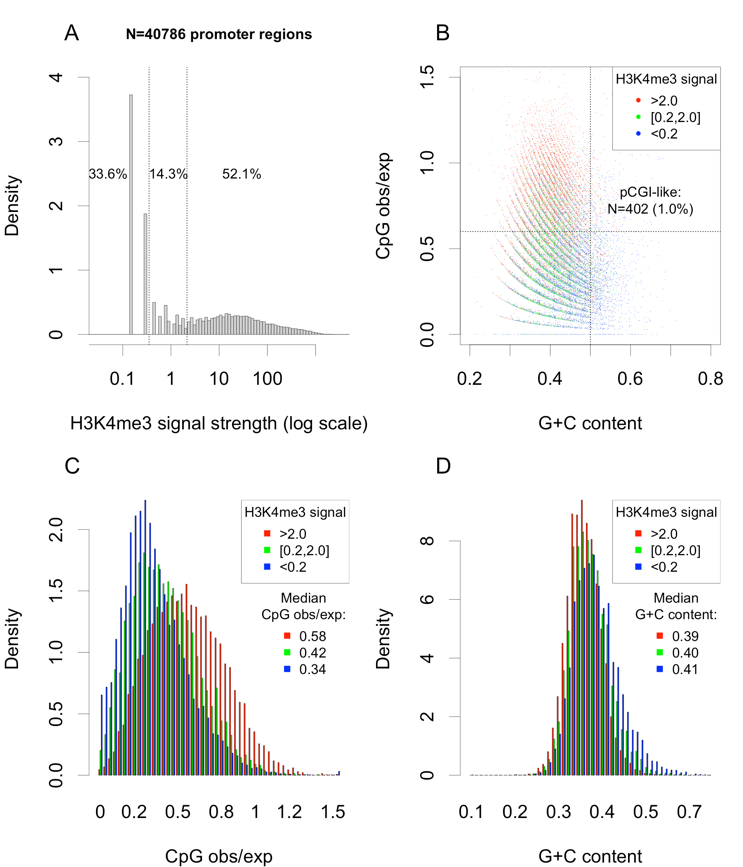


**Fig C: Relationship between base composition and H3K4me3 in promoter regions of the rainbow trout (*Oncorhynchus mykiss*).** We selected protein-coding genes (N=40,786) annotated in the rainbow trout genome and extracted their promoter region, defined as the 500 bp upstream of the TSS. For each promoter region, we quantified the H3K4me3 level (ChIPseq data from a brain sample). **A)** Distribution of H3K4me3 signal within promoter regions. **B)** CpG observed/expected ratio vs. G+C content of promoter regions. Promoters were classified according to their CpG methylation level (red: hypomethylated; blue: highly methylated; green: intermediate methylation level). The number and percentage of promoter regions matching the classical criteria for CGI annotation (CpG obs/exp>0.6 and G+C content >0.5) are indicated. **C)** Distribution of CpG observed/expected ratio of promoter regions, for different classes of H3K4me3 signal. **D)** Distribution of G+C content of promoter regions, for different classes of H3K4me3 signal.

We identified 560,469 *fpCGIs* in the rainbow trout genome, and among the 40,786 annotated TSSs, 18,356 (45.0%) are located close to a *fpCGI* (<250 bp). Again, we observed that the presence of a *fpCGI* is informative regarding the epigenetic status of the promoter region: among *fpCGI*-associated TSSs, 69.4% display a strong H3K4me3 signal, compared to only 7.8% for TSSs located far from a *fpCGI* (>1000 bp) (**Fig D**). This 8.9-fold enrichment indicates that the presence of a nearby *fpCGI* is a very good predictor of the chromatin state of the promoter region.

CpG-island annotations were available from UCSC for the same rainbow trout genome assembly (N=18,220 *pCGI*s), allowing us to evaluate the capacity of this commonly used resource to predict salmonid CGIs. Only 0.7% of rainbow trout TSSs are located at less than 250 bp from a UCSC *pCGI*. Among these *pCGI*-associated TSSs, 53.9% display a strong H3K4me3 signal (compared to 69.4% for *fpCGI*-associated TSSs). These results indicate i) that a majority of TSS-associated CGIs are missed by UCSC *pCGI* annotations, and ii) that the *fpCGIs* are better predictors of the epigenetic state of promoters than UCSC *pCGIs*.

**
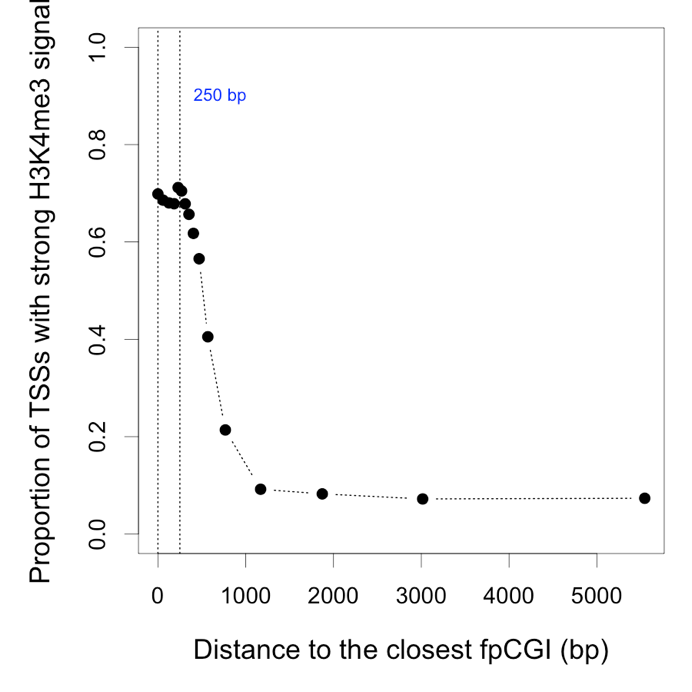
**

**Fig D: Relationship between H3K4me3 in the promoter regions of the rainbow trout and the presence of a nearby *fpCGI*.** We classified TSSs according to their distance to the nearest *fpCGI*, and computed in each bin, the proportion of TSS with a strong H3K4me3 signal in their promoter region.

## Conclusion

Both in rainbow trout and in coho salmon, we observed that 52% to 59% of promoter regions present the typical hallmarks of CGIs: low DNA methylation level, high signal of H3K4me3, high *CpG_oe_*. However, as in zebrafish, but contrary to mammals and birds, these GCIs are not G+C-rich. Hence, the criteria that are used classically to predict CGIs (*CpG_oe_*>0.6 and G+C>50%) fail to detect a large majority of the CGIs present in salmonid genomes. We therefore propose that in these species, CGIs should be predicted solely based on their *CpG_oe_*, not on their G+C-content. The very high number of *fpCGI*s identified in the whole genome (>500,000 *fpCGI*s) suggests that many of them are false positives. However, we showed that the presence or absence of *fpCGI*s in the vicinity of TSSs is a very good predictor of their epigenetic status (DNA methylation level, H3K4me3) of promoter regions. Hence, the criteria that we used to annotate *fpCGI*s (DNA segment > 250 bp, with *CpG_oe_*>0.6) appear appropriate to predict CGI-associated promoters in salmonids.

## Material and methods

### Reference genome assembly, annotation of TSSs and promoter regions

We retrieved genome sequences and annotations from NCBI:

- Coho salmon (*Oncorhynchus kisutch*): Okis_V1 (GCF_002021735.1)
- Rainbow trout (*Oncorhynchus mykiss*): USDA_OmykA_1.1 (GCF_013265735.2)

For each protein-coding gene, we selected the transcript encoding the longest CDS, and defined the TSS as the 5’end of this transcript. We excluded genes located on the mitochondrial genome, or on unmapped contigs.

We defined the ‘promoter region’ as the 500 bp-long segment in 5’ of the TSS. We computed the G+C content of sequences, and excluded promoter regions containing more than 100 undetermined bases (N’s).

The CpGoe of promoter regions was calculated according to the formula:

CpGoe = Number of CpG * L / (Number of C * Number of G)

where L = length of sequence.

### Prediction of CGIs

We used the *cpgplot* software (from the EMBOSS package) to identify genomic DNA segments matching the following criteria:

- length > 250 bp
- CpGoe > 0.6

We used the following command line:

cpgplot -sequence genome.fa -minlen 250 -minpc 0. -minoe 0.6 -window 500 -noplot

These DNA segments will hereafter be referred to as ‘fish putative CGIs’ (*fpCGIs*).

In addition, for the rainbow trout, we retrieved annotated *pCGIs* from the UCSC genome browser: <http://genome.ucsc.edu/cgi-bin/hgTables?hgsid=1701022328_0XcuiZkramud5mBRGsZxZ4CT99HY&clade=hub_2243217&org=hub_2243217_USDA_OmykA_1.1+Sep.+2020&db=hub_2243217_GCF_013265735.2&hgta_group=allTracks&hgta_track=hub_2243217_cpgIslands&hgta_table=0&hgta_regionType=genome&position=NC_048566.1%3A34%2C602%2C292-34%2C612%2C292&hgta_outputType=sequence&hgta_outFileName=>

These UCSC *pCGIs* have been predicted based on the ‘classical’ criteria (CpGoe > 0.6 and G+C content > 0.5).

### DNA methylation and H3K4me3 data

We retrieved DNA methylation data from coho salmon, obtained by whole genome bisulfite sequencing (16) (NCBI project accession PRJNA678281). The BED file of a liver sample (NCBI biosample accession SAMN25653842) was kindly provided by Maeva Leitwein.

We retrieved H3K4me3 ChIPseq data from a rainbow trout brain sample (<https://data.faang.org/dataset/PRJEB57956>).

**References**

1. de Mendoza A, Lister R, Bogdanovic O. Evolution of DNA Methylome Diversity in Eukaryotes. J Mol Biol. 2020;432(6):1687-705.

2. Long HK, Sims D, Heger A, Blackledge NP, Kutter C, Wright ML, et al. Epigenetic conservation at gene regulatory elements revealed by non-methylated DNA profiling in seven vertebrates. eLife. 2013;2:e00348.

3. Tweedie S, Charlton J, Clark V, Bird A. Methylation of genomes and genes at the invertebrate-vertebrate boundary. Mol Cell Biol. 1997;17(3):1469-75.

4. Bird AP. CpG-rich islands and the function of DNA methylation. Nature. 1986;321(6067):209-13.

5. Deaton AM, Bird A. CpG islands and the regulation of transcription. Genes Dev. 2011;25(10):1010-22.

6. Bird AP. DNA methylation and the frequency of CpG in animal DNA. Nucleic Acids Res. 1980;8(7):1499-504.

7. Cohen NM, Kenigsberg E, Tanay A. Primate CpG islands are maintained by heterogeneous evolutionary regimes involving minimal selection. Cell. 2011;145(5):773-86.

8. Joseph J, Prentout D, Laverre A, Tricou T, Duret L. High prevalence of PRDM9-independent recombination hotspots in placental mammals. Proc Natl Acad Sci U S A. 2024;121(23):e2401973121.

9. Auton A, Rui Li Y, Kidd J, Oliveira K, Nadel J, Holloway JK, et al. Genetic Recombination Is Targeted towards Gene Promoter Regions in Dogs. PLoS Genet. 2013;9(12):e1003984.

10. Kawakami T, Mugal CF, Suh A, Nater A, Burri R, Smeds L, et al. Whole-genome patterns of linkage disequilibrium across flycatcher populations clarify the causes and consequences of fine-scale recombination rate variation in birds. Mol Ecol. 2017;26(16):4158-72.

11. Singhal S, Leffler EM, Sannareddy K, Turner I, Venn O, Hooper DM, et al. Stable recombination hotspots in birds. Science. 2015;350(6263):928-32.

12. Hoge C, de Manuel M, Mahgoub M, Okami N, Fuller Z, Banerjee S, et al. Patterns of recombination in snakes reveal a tug-of-war between PRDM9 and promoter-like features. Science. 2024;383(6685):eadj7026.

13. Schield DR, Pasquesi GIM, Perry BW, Adams RH, Nikolakis ZL, Westfall AK, et al. Snake Recombination Landscapes Are Concentrated in Functional Regions despite PRDM9. Mol Biol Evol. 2020;37(5):1272-94.

14. Gardiner-Garden M, Frommer M. CpG islands in vertebrate genomes. J Mol Biol. 1987;196(2):261-82.

15. Cross S, Kovarik P, Schmidtke J, Bird A. Non-methylated islands in fish genomes are GC-poor. Nucleic Acids Res. 1991;19(7):1469-74.

16. Leitwein M, Wellband K, Cayuela H, Le Luyer J, Mohns K, Withler R, et al. Strong Parallel Differential Gene Expression Induced by Hatchery Rearing Weakly Associated with Methylation Signals in Adult Coho Salmon (O. kisutch). Genome Biol Evol. 2022;14(4).
