# Supplementary material for: PRDM9 drives the location and rapid evolution of recombination hotspots in salmonid fish
Source: PLoS Biol. 2025 Jan 6;23(1):e3002950. doi: 10.1371/journal.pbio.3002950 (PMC11703093; doi:10.1371/journal.pbio.3002950)
Supplement: S1 Table — The index allows to identify the corresponding copy in the phylogeny of the ɑ paralog copies in Fig 1A and the β copies in S1 Fig. We retrieved the location of the regions covering the 3 domains KRAB, SSXRD, and SET obtained from the blast analysis. The start and end positions correspond to the start position of the first exon blasted and the end position of the last exon blasted. (DOCX) [file pbio.3002950.s003.docx]

**S1 Table: Chromosome location of the retained PRDM9 paralog copies.** The index allows to identify the corresponding copy in the phylogeny of the ɑ paralog copies in **Fig** **1A** and the β copies in **S1 Fig**. We retrieved the location of the regions covering the three domains KRAB, SSXRD and SET obtained from the blast analysis. The start and end positions correspond to the start position of the first exon blasted and the end position of the last exon blasted.

| **Species** | **Index** | **Reference genome RefSeq accession number** | **Chromosome number and / or RefSeq accession number** | **Start position** | **End position** |  |
| --- | --- | --- | --- | --- | --- | --- |
| **Prdm9ɑ paralogs** | | | | | | |
| *O. kisutch* | 1 | GCF_002021735.2 | NC_034188.2  (chr. 15) | 12887053 | 12894097 |  |
|  | 2 |  | NC_034192.2  (chr. 19) | 56290129 | 56303034 |  |
|  | 3 |  | NC_034178.2  (chr. 5) | 5473664 | 5478378 |  |
|  | 4 |  | NC_034188.2  (chr. 15) | 13227731 | 13233628 |  |
|  | 5 |  | NC_034192.2  (chr. 19) | 56235978 | 56236786 |  |
| *O. mykiss* | 6 | GCF_002163495.1 | NC_035101.1  (chr. 25) | 8415083 | 8423079 |  |
|  | 7 |  | NC_035090.1  (chr. 14) | 72512843 | 72525023 |  |
|  | 8 | GCF_013265735.2 | NC_048571.1  (chr. 7) | 16078839 | 16097818 |  |
|  | 9 | GCF_002163495.1 | NC_035101.1  (chr. 25) | 8732180 | 8735814 |  |
| *S. salar* | 10 | GCF_905237065.1 | NC_059450.1  (chr. 9) | 87343648 | 87355556 |  |
|  | 11 |  | NC_059446.1  (chr. 5) | 12774207 | 12794545 |  |
|  | 12 |  | NC_059458.1  (chr. 17) | 19507805 | 19530082 |  |
|  | 13 |  | NC_059457.1  (chr. 16) | 72831017 | 72843243 |  |
|  | 14 |  | NC_059446.1  (chr. 5) | 12827488 | 12840869 |  |
|  | 15 |  | NC_059450.1  (chr. 9) | 87687617 | 87688156 |  |
| *O. nerka* | 16 | GCF_006149115.2 | NW_021798387.1 | 5360 | 13922 |  |
|  | 17 |  | NW_021812881.1 | 27422 | 38545 |  |
|  | 18 |  | NW_021787950.1 | 49862 | 52085 |  |
|  | 19 |  | NW_021810464.1 | 37685 | 38512 |  |
|  | 20 |  | NW_021792348.1 | 114852 | 115615 |  |
| *O. keta* | 21 | GCF_023373465.1 | NC_068450.1 | 14332662 | 14340913 |  |
|  | 22 |  | NC_068424.1 | 74859328 | 74871640 |  |
|  | 23 |  | NC_068457.1 | 17803818 | 17834627 |  |
|  | 24 |  | NW_026283388.1 | 13799 | 14605 |  |
|  | 25 |  | NC_068450.1 | 14795938 | 14799988 |  |
| *O. tshawytscha* | 26 | GCF_018296145.1 | NC_056436.1 | 78654218 | 78662610 |  |
|  | 27 |  | NC_056449.1 | 33170582 | 33187287 |  |
|  | 28 |  | NW_024609832.1 | 379135 | 382678 |  |
|  | 29 |  | NC_056449.1 | 33127116 | 33138001 |  |
|  | 30 |  | NC_056436.1 | 78152338 | 78156301 |  |
| *O. gorbuscha* | 31 | GCF_021184085.1 | NC_060195.1 | 13395381 | 13404061 |  |
|  | 32 |  | NC_060185.1 | 88927636 | 88981659 |  |
|  | 33 |  | NC_060193.1 | 53131373 | 53143551 |  |
|  | 34 |  | NC_060195.1 | 13858179 | 13860498 |  |
|  | 35 |  | NC_060184.1 | 84369277 | 84370834 |  |
| *S. trutta* | 36 | GCA_901001165.2 | LR584428.1 | 31835604 | 31845878 |  |
|  | 37 |  | LR584416.1 | 9894917 | 9917525 |  |
|  | 38 |  | LR584413.1 | 12304219 | 12315348 |  |
|  | 39 |  | LR584406.1 | 51081287 | 51092978 |  |
|  | 40 |  | LR584416.1 | 9946574 | 9958847 |  |
|  | 41 |  | CAAJIE020000684.1 | 38776 | 41222 |  |
| *S. namaycush* | 42 | GCF_016432855.1 | NC_052323.1 | 34467596 | 34477759 |  |
|  | 43 |  | NC_052348.1 | 18528766 | 18529803 |  |
|  | 44 |  | NC_052313.1 | 49372386 | 49380629 |  |
|  | 45 |  | NC_052346.1 | 12719793 | 13806471 |  |
|  | 46 |  | NC_052323.1 | 20296993 | 20315388 |  |
| *C. clupeaformis* | 47 | GCF_020615455.1 | NC_059212.1 | 8991317 | 9007109 |  |
|  | 48 |  | NC_059226.1 | 40101271 | 40117907 |  |
|  | 49 |  | NW_025534320.1 | 170337 | 176421 |  |
|  | 50 |  | NW_025535102.1 | 51554 | 67751 |  |
|  | 51 |  | NW_025537026.1 | 17152 | 33482 |  |
|  | 52 |  | NC_059194.1 | 2849538 | 2871608 |  |
|  | 53 |  | NC_059194.1 | 2824237 | 2832607 |  |
|  | 54 |  | NW_025537642.1 | 2376 | 21348 |  |
|  | 55 |  | NW_025533470.1 | 712759 | 713888 |  |
|  | 56 |  | NW_025533470.1 | 810220 | 811146 |  |
|  | 57 |  | NW_025533877.1 | 108337 | 114502 |  |
|  | 58 |  | NW_025534294.1 | 39024 | 42742 |  |
| *T. thymallus* | 59 | GCA_023634145.1 | CM042384.1 | 27241336 | 27251246 |  |
|  | 60 |  | CM042385.1 | 6555373 | 6788464 |  |
|  | 61 |  | CM042384.1 | 27035181 | 27035496 |  |
| *H. hucho* | 62 | GCA_003317085.1 | QNTS01002334.1 | 29710 | 39792 |  |
|  | 63 |  | QNTS01002761.1 | 75215 | 83155 |  |
|  | 64 |  | QNTS01003609.1 | 55551 | 56973 |  |
|  | 65 |  | QNTS01000290.1 | 78088 | 82204 |  |
|  | 66 |  | QNTS01015822.1 | 8019 | 8541 |  |
| *E. lucius* | 67 | GCF_011004845.1 | NC_047593.1 | 21477662 | 21488947 |  |
|  | 68 |  | NC_047587.1 | 2136731 | 2399395 |  |
| **Prdm9β paralogs** | | | | | | |
| *O. kisutch* | 1 | GCF_002021735.2 | NC_034198.2  (chr. 25) | 25039623 | 25040671 |  |
|  | 2 |  | NC_034184.2  (chr. 11) | 7538438 | 7540180 |  |
| *O. mykiss* | 3 | GCF_002163495.1 | NC_035096.1  (chr. 20) | 23688475 | 23689502 |  |
|  | 4 |  | NC_035099.1  (chr. 23) | 42290320 | 42293196 |  |
| *S. salar* | 5 | GCF_905237065.1 | NC_059469.1  (chr. 28) | 18949696 | 18950780 |  |
|  | 6 |  | NC_059442.1  (chr. 1) | 94679893 | 94684082 |  |
| *O. nerka* | 7 | GCF_006149115.2 | NC_042550.1 | 22686397 | 22687439 |  |
|  | 8 |  | NW_021789518.1 | 42389 | 44985 |  |
| *O. keta* | 9 | GCF_023373465.1 | NC_068444.1 | 25615799 | 25616838 |  |
|  | 10 |  | NC_068422.1 | 7947206 | 7950309 |  |
| *O. tshawytscha* | 11 | GCF_018296145.1 | NC_056453.1 | 25860865 | 25861913 |  |
|  | 12 |  | NC_056429.1 | 88507466 | 88510273 |  |
| *O. gorbuscha* | 13 | GCF_021184085.1 | NC_060179.1 | 66056844 | 66057889 |  |
|  | 14 |  | NC_060178.1 | 98157196 | 98161663 |  |
| *S. trutta* | 15 | GCA_901001165.2 | LR584419.1 | 25732352 | 25733424 |  |
|  | 16 |  | LR584435.1 | 14382999 | 14387213 |  |
| *S. namaycush* | 17 | GCF_016432855.1 | NC_052341.1 | 15666464 | 15667511 |  |
|  | 18 |  | NC_052310.1 | 78263958 | 78266635 |  |
| *C. clupeaformis* | 19 | GCF_020615455.1 | NC_059222.1 | 13680015 | 13681061 |  |
|  | 20 |  | NC_059192.1 | 95051878 | 95052654 |  |
| *T. thymallus* | 21 | GCA_023634145.1 | CM042387.1 | 22808132 | 22809233 |  |
|  | 22 |  | CM042388.1 | 11440396 | 11441186 |  |
| *H. hucho* | 23 | GCA_003317085.1 | QNTS01001395.1 | 107566 | 108622 |  |
|  | 24 |  | QNTS01001493.1 | 122445 | 127178 |  |
| *E. lucius* | 25 | GCF_011004845.1 | NC_047573.1 | 20413747 | 20414822 |  |
| *D. labrax* | 26 | GCF_905237075.1 | NW_026136712.1 | 18707566 | 18712966 |  |
