# Supplementary material for: PRDM9 drives the location and rapid evolution of recombination hotspots in salmonid fish
Source: PLoS Biol. 2025 Jan 6;23(1):e3002950. doi: 10.1371/journal.pbio.3002950 (PMC11703093; doi:10.1371/journal.pbio.3002950)
Supplement: S2 Table — Summary statistics of the variations in recombination rates smoothed in 2 kb sliding windows, and of recombination hotspots retrieved from the inter-SNP recombination landscapes. The raw hotspots were defined as the consecutives inter-SNP windows with a recombination rate 5-fold higher than the 50 kb flanking regions. (DOCX) [file pbio.3002950.s004.docx]

**S2 Table: Fine scale variations in recombination rates and raw recombination hotspots.** Summary statistics of the variations in recombination rates smoothed in 2 kb sliding windows, and of recombination hotspots retrieved from the inter-SNP recombination landscapes. The raw hotspots were defined as the consecutives inter-SNP windows with a recombination rate five-fold higher than the 50 kb flanking regions.

|  | ***O. kisutch*** | ***O. mykiss*** | ***S. salar*** | | | ***D. labrax*** |
| --- | --- | --- | --- | --- | --- | --- |
|  |  |  | **GP** | **BS** | **NS** |  |
| **Genome-wide recombination rates** | | | | | | |
| Genome-wide recombination rate (⍴/bp) | 0,0032 | 0.0123 | 0.0084 | 0.0065 | 0.0085 | 0.039 |
| Variation range of recombination rates (smoothed at 2 kb) | [4.49x10-7; 7.6] | [3.83x10-7; 5.07] | [2.48x10-7; 7.86] | [6.47x10-8; 5.82] | [7.33x10-8; 4.23] | [3.26x10-7; 5.68] |
| Percent of recombination in 20% of the genome | 90.1 % | 89.1 % | 98.3 % | 98 % | 98.1 % | 84.6 % |
| **Raw recombination hotspots** | | | | | | |
| Number of hotspots | 49742 | 90696 | 25139 | 25973 | 26550 | 7897 |
| Mean fold recombination rate in hotspots | 19 | 19.2 | 19.6 | 19.8 | 20.5 | 18.9 |
| Mean hotspots size (in bp) | 418 | 309 | 927 | 980 | 1073 | 1995 |
| Fraction of recombination in hotspots | 24% | 17% | 34% | 37% | 39% | 3% |
| Proportion of hotspots shorter than 2 kb | 93,5 % | 97,5 % | 80,1 % | 76 % | 73,1 % | 63.7% |
