# Supplementary material for: PRDM9 drives the location and rapid evolution of recombination hotspots in salmonid fish
Source: PLoS Biol. 2025 Jan 6;23(1):e3002950. doi: 10.1371/journal.pbio.3002950 (PMC11703093; doi:10.1371/journal.pbio.3002950)
Supplement: S3 Table — Overlaps were assessed for 400-bp wide windows centered on DSB hotspot centers and TSS/TES regions, defined as sequences found within 1 kb of distance from the transcription start/end site. The expected overlaps were estimated as the chance for 2 kb windows of overlapping 400 bp DSB hotspots genome wide. (DOCX) [file pbio.3002950.s005.docx]

**S3 Table: Overlaps between DSB hotspots and TSS/TES regions.** Overlaps were assessed for 400 bp-wide windows centered on DSB hotspot centers and TSS/TES regions, defined as sequences found within 1 kb of distance from the transcription start/end site. The expected overlaps were estimated as the chance for 2 kb windows of overlapping 400 bp DSB hotspots genome-wide.

| **Sample** | **TSS category** | **# DSB HS** | **# TSS** | **Overlap. DSB HS** | **% of overlap. DSB HS** | **Exp. Overlap. DSB HS** | **Exp. % of overlap. DSB HS** | **p-value (chi2)** |
| --- | --- | --- | --- | --- | --- | --- | --- | --- |
| **DSB hotspots and TSS** | | | | | | | | |
| RT-52 | all genes | 1924 | 72603 | 96 | **5.0%** | 146 | 7.6% | 1.7E-05 |
| TAC-1 | all genes | 616 | 72603 | 28 | **4.5%** | 47 | 7.6% | 0.0039 |
| TAC-3 | all genes | 209 | 72603 | 11 | **5.3%** | 16 | 7.6% | 0.19 |
| RT-52 | protein coding genes | 1924 | 41890 | 89 | **4.6%** | 84 | 4.4% | 0.58 |
| TAC-1 | protein coding genes | 616 | 41890 | 24 | **3.9%** | 27 | 4.4% | 0.55 |
| TAC-3 | protein coding genes | 209 | 41890 | 7 | **3.3%** | 9 | 4.4% | 0.50 |
| RT-52 | non-coding genes | 1924 | 30713 | 7 | **0.4%** | 62 | 3.2% | 1.2E-12 |
| TAC-1 | non-coding genes | 616 | 30714 | 4 | **0.6%** | 20 | 3.2% | 0.00028 |
| TAC-3 | non-coding genes | 209 | 30715 | 4 | **1.9%** | 7 | 3.2% | 0.25 |
| **DSB hotspots and TES** | | | | | | | | |
| RT-52 | all genes | 1924 | 72589 | 164 | **8.5%** | 146 | 7.6% | 0.12 |
| TAC-1 | all genes | 616 | 72589 | 45 | **7.3%** | 47 | 7.6% | 0.76 |
| TAC-3 | all genes | 209 | 72589 | 22 | **10.5%** | 16 | 7.6% | 0.12 |
| RT-52 | protein coding genes | 1924 | 41886 | 150 | **7.8%** | 84 | 4.4% | 1.79E-13 |
| TAC-1 | protein coding genes | 616 | 41886 | 39 | **6.3%** | 27 | 4.4% | 0.018 |
| TAC-3 | protein coding genes | 209 | 41886 | 15 | **7.2%** | 9 | 4.4% | 0.041 |
| RT-52 | non-coding genes | 1924 | 30703 | 17 | **0.9%** | 62 | 3.2% | 6.27E-09 |
| TAC-1 | non-coding genes | 616 | 30703 | 7 | **1.1%** | 20 | 3.2% | 0.0031 |
| TAC-3 | non-coding genes | 209 | 30703 | 7 | **3.3%** | 7 | 3.2% | 1 |
