# Supplementary material for: PRDM9 drives the location and rapid evolution of recombination hotspots in salmonid fish
Source: PLoS Biol. 2025 Jan 6;23(1):e3002950. doi: 10.1371/journal.pbio.3002950 (PMC11703093; doi:10.1371/journal.pbio.3002950)
Supplement: S4 Table — Information is retrieved on the reference genome sizes, population sample sizes, mapping depth statistics, variant calling statistics, and effective sizes of genomic features for each population. (DOCX) [file pbio.3002950.s006.docx]

**S4 Table: Summary statistics of the LD-landscape reconstruction pipeline.** Information is retrieved on the reference genome sizes, population sample sizes, mapping depth statistics, variant calling statistics and effective sizes of genomic features for each population.

|  | ***O. kisutch*** | ***O. mykiss*** | ***S. salar*** | | | ***D. labrax*** |
| --- | --- | --- | --- | --- | --- | --- |
|  |  |  | **GP** | **BS** | **NS** |  |
| **Reference genome** | | | | | | |
| Genome size | 1,686,580,692 | 1,949,962,539 | 2,499,322,922 | | | 578,963,054 |
| Number of chromosomes | 30 | 29 | 29 | | | 24 |
| **Sample collection** | | | | | | |
| Number of samples | 20 | 22 | 20 | 20 | 20 | 14 |
| **Mapping statistics** | | | | | | |
| Mean depth per individual | 29.54X | 24.87X | 9.97X | | | >20X |
| Range of mean depth per individual | [23.47-32.95] | [10.97-31.27] | [7.43-13.07] | | | ~[15-40] |
| **Variant calling and filtering statistics** | | | | | | |
| Number of SNPs after variant calling | 9,590,270 | 38,601,311 | 27,061,466 | | | 14,579,961 |
| Number of SNPs after filtering | 5,133,567 | 10,797,232 | 2,829,055 | 2,700,533 | 2,521,890 | 5,074,249 |
| SNP density (per bp) after filtering | 0.0031 | 0.0055 | 0.0011 | 0.0011 | 0.0010 | 0.009 |
| **Genomic features** | | | | | | |
| Number of genes | 31,150 | 44,615 | 52,016 | | | 18,536 |
| Number of exons | 600,584 | 836,394 | 1,405,313 | | | 192,583 |
| Number of introns | 289,493 | 370,185 | 432,062 | | | 175,218 |
| Number of TSS | 31,137 | 44,615 | 52,015 | | | 18,191 |
| Number of TES | 31,132 | 44,588 | 51,988 | | | 18,141 |
| Number of CGIs | 466,523 | 684,137 | 648,505 | | | 131,512 |
| Percent of TSS in CGIs | 60% | 58% | 57% | | | 38% |
| Percent of TEs elements | 47.37% | 48.56% | 52.26% | | | *NA* |
| Total length of interspersed repeats (in bp) | 1,122,660,520 | 1,058,078,392 | 1,440,595,994 | | | *NA* |
| % Tc1-mariner | 13.16% | 14.7% | 12.48% | | | *NA* |
| Number of Tc1 mariner | 799,417 | 748,980 | 782,170 | | | *NA* |
