# Supplementary material for: PRDM9 drives the location and rapid evolution of recombination hotspots in salmonid fish
Source: PLoS Biol. 2025 Jan 6;23(1):e3002950. doi: 10.1371/journal.pbio.3002950 (PMC11703093; doi:10.1371/journal.pbio.3002950)
Supplement: S5 Table — (DOCX) [file pbio.3002950.s007.docx]

**S5 Table: Comparison of fine-scale recombination landscapes across vertebrates**

| **Clade** | **Species** | **Common name** | **Prdm9** | **Reference** | **Method** | **F20 (a)** | **Number of hotspots reported (b)** | **Genome size (Gb)** | **Number of hotspots/Gb** | **Data source** |
| --- | --- | --- | --- | --- | --- | --- | --- | --- | --- | --- |
| Aves | *Ficedula hypoleuca* | Pied flycatcher | No Prdm9 | (1) | Ldhelmet | 0.57 | 2500 | 1.1 | 2272.73 | <https://datadryad.org/stash/dataset/doi:10.5061/dryad.hp5h2> |
| Aves | *Sylvia atricapilla* | Eurasian blackcap | No Prdm9 | (2) | pyrho | 0.67 | ND | 1.1 | ND | <https://zenodo.org/records/10234492> |
| Aves | *Taeniopygia guttata* | Zebra finch | No Prdm9 | (3) | Ldhelmet | 0.83 | 3949 | 1.1 | 3590.00 | <https://datadryad.org/stash/dataset/doi:10.5061/dryad.fd24j> |
| Aves | *Tyto alba* | Barn owl | No Prdm9 | (4) | pyrho | 0.63 | 3949 | 1.2 | 3290.83 | F20 based on Fig. 2C from (4) |
| Mammalia | *Canis lupus familiaris* | Dog | No Prdm9 | (5) | Ldhat | 0.73 | 7677 | 2.5 | 3070.80 | <https://github.com/auton1/dog_recomb> |
| Mammalia | *Chlorocebus aethiops sabaeus* | Vervet Monkeys | Full-length Prdm9 | (6) | Ldhat | 0.99 | ND | 2.9 | ND | <http://spfeiferlab.org/wp-content/uploads/2020/04/LDhat_rate_estimates.zip> |
| Mammalia | *Homo sapiens* | Human | Full-length Prdm9 | (7) | Ldhat | 0.87 | 32996 | 3.1 | 10643.87 | <https://ftp.ncbi.nlm.nih.gov/hapmap/recombination/latest/rates/> |
| Mammalia | *Macaca mulatta* | Rhesus Macaque | Full-length Prdm9 | (8) | pyrho | 0.64 | ND | 3 | ND | <http://spfeiferlab.org/wp-content/uploads/2023/10/pyrho_rate_estimates.tar.gz> |
| Mammalia | *Mus musculus castaneus* | Mouse | Full-length Prdm9 | (9) | LDhelmet | 0.78 | 39972 | 2.7 | 14804.44 | <https://github.com/TBooker/M.m.castaneus_recombination-maps> |
| Mammalia | *Pan troglodytes verus* | Western chimpanzee | Full-length Prdm9 | (10) | Ldhat | 0.82 | 5026 | 3.2 | 1570.63 | ftp://birch.well.ox.ac.uk/panMap/ (downloaded in 2012; link no longer active) |
| Mammalia | *Papio anubis* | Olive baboon | Full-length Prdm9 | (11) | pyrho | 0.78 | ND | 2.9 | ND | <https://datadryad.org/stash/dataset/doi:10.7272/Q6HH6H9D> |
| Squamata | *Pantherophis guttatus* | Corn snake | Full-length Prdm9 | (12) | LDhelmet | 0.78 | 13580 | 1.7 | 7988.24 | <https://zenodo.org/records/10197003> |
| Teleostei | *Astatotilapia calliptera* | Cichlid fish | KRAB-less Prdm9 | (13) | pyrho | 0.62 | 2322 | 0.9 | 2580.00 | F20 based on Fig. 2B from (13) |
| Teleostei | *Dicentrarchus labrax* | European sea bass | KRAB-less Prdm9 | this study | LDhelmet | 0.85 | 7897 | 0.7 | 11281.43 | this study |
| Teleostei | *Gasterosteus aculeatus* | Threespine Stickleback | KRAB-less Prdm9 | (14) | LDhelmet | 0.72 | 2368 | 0.5 | 4736.00 | Kindly provided by the authors |
| Teleostei | *Oncorhynchus kisutch* | Coho salmon | Full-length Prdm9 | this study | LDhelmet | 0.90 | 22948 | 2.4 | 9561.67 | this study |
| Teleostei | *Oncorhynchus mykiss* | Rainbow trout | Full-length Prdm9 | this study | LDhelmet | 0.89 | 21145 | 2.3 | 9193.48 | this study |
| Teleostei | *Salmo salar* | Atlantic salmon | Full-length Prdm9 | this study | LDhelmet | 0.98 | 17064 | 2.8 | 6094.29 | this study |

(a): F20: fraction of recombination in the 20% of the genome with the highest recombination rate

(b): Number of recombination hotspots reported by the authors. NB: the criteria that have been used to define hotspots vary across studies

**References**

1. Kawakami T, Mugal CF, Suh A, Nater A, Burri R, Smeds L, et al. Whole-genome patterns of linkage disequilibrium across flycatcher populations clarify the causes and consequences of fine-scale recombination rate variation in birds. Mol Ecol. 2017;26(16):4158-72.

2. Bascon-Cardozo K, Bours A, Manthey G, Durieux G, Dutheil JY, Pruisscher P, et al. Fine-Scale Map Reveals Highly Variable Recombination Rates Associated with Genomic Features in the Eurasian Blackcap. Genome Biol Evol. 2024;16(1).

3. Singhal S, Leffler EM, Sannareddy K, Turner I, Venn O, Hooper DM, et al. Stable recombination hotspots in birds. Science. 2015;350(6263):928-32.

4. Topaloudis A, Lavanchy E, Cumer T, Ducrest A-L, Simon C, Machado AP, et al. The recombination landscape of the barn owl, from families to populations. bioRxiv. 2024:2024.04.11.589103.

5. Auton A, Rui Li Y, Kidd J, Oliveira K, Nadel J, Holloway JK, et al. Genetic Recombination Is Targeted towards Gene Promoter Regions in Dogs. PLoS Genet. 2013;9(12):e1003984.

6. Pfeifer SP. A Fine-Scale Genetic Map for Vervet Monkeys. Mol Biol Evol. 2020;37(7):1855-65.

7. Frazer KA, Ballinger DG, Cox DR, Hinds DA, Stuve LL, Gibbs RA, et al. A second generation human haplotype map of over 3.1 million SNPs. Nature. 2007;449(7164):851-61.

8. Versoza CJ, Weiss S, Johal R, La Rosa B, Jensen JD, Pfeifer SP. Novel Insights into the Landscape of Crossover and Noncrossover Events in Rhesus Macaques (Macaca mulatta). Genome Biol Evol. 2024;16(1).

9. Booker TR, Ness RW, Keightley PD. The Recombination Landscape in Wild House Mice Inferred Using Population Genomic Data. Genetics. 2017;207(1):297-309.

10. Auton A, Fledel-Alon A, Pfeifer S, Venn O, Segurel L, Street T, et al. A Fine-Scale Chimpanzee Genetic Map from Population Sequencing. Science. 2012;336(6078):193-8.

11. Wall JD, Robinson JA, Cox LA. High-Resolution Estimates of Crossover and Noncrossover Recombination from a Captive Baboon Colony. Genome Biol Evol. 2022;14(4).

12. Hoge C, de Manuel M, Mahgoub M, Okami N, Fuller Z, Banerjee S, et al. Patterns of recombination in snakes reveal a tug-of-war between PRDM9 and promoter-like features. Science. 2024;383(6685):eadj7026.

13. Talbi M, Turner GF, Malinsky M. Rapid evolution of recombination landscapes during the divergence of cichlid ecotypes in Lake Masoko. bioRxiv. 2024:2024.03.20.585960.

14. Shanfelter AF, Archambeault SL, White MA. Divergent Fine-Scale Recombination Landscapes between a Freshwater and Marine Population of Threespine Stickleback Fish. Genome Biol Evol. 2019;11(6):1573-85.
