# Supplementary material for: PRDM9 drives the location and rapid evolution of recombination hotspots in salmonid fish
Source: PLoS Biol. 2025 Jan 6;23(1):e3002950. doi: 10.1371/journal.pbio.3002950 (PMC11703093; doi:10.1371/journal.pbio.3002950)
Supplement: S6 Table — (DOCX) [file pbio.3002950.s008.docx]

**S6 Table: Presence of *Zcwpw1*, *Zcwpw2*, *Tex15* and *Fbox47* genes in the genomes of analyzed species**

| **Gene** | **Species** | **Status** | **RefSeq Accession** | **AA Length** | **Domain Architecture** | **Definition** |
| --- | --- | --- | --- | --- | --- | --- |
| ***Zcwpw1*** | *Salmo salar* | present | XP_045577337.1 | 682 | zfCW-PWWP | zinc finger CW-type PWWP domain protein 1-like isoform X3 |
|  | *Oncorhynchus mykiss* | present | XP_036814305.1 | 769 | zfCW-PWWP | zinc finger CW-type PWWP domain protein 1 isoform X1 |
|  | *Oncorhynchus kisutch* | present | XP_031688551.1 (a) | 765 (a) | zfCW-PWWP | zinc finger CW-type PWWP domain protein 1 isoform X1 |
|  | *Dicentrarchus labrax* | not found (b) | NA | NA | NA | NA |
| ***Zcwpw2*** | *Salmo salar* | present | XP_014041947.1 | 271 | zfCW-PWWP | zinc finger CW-type PWWP domain protein 2-like isoform X2 |
|  | *Oncorhynchus mykiss* | present | XP_021442531.2 | 271 | zfCW-PWWP | zinc finger CW-type PWWP domain protein 2 isoform X2 |
|  | *Oncorhynchus kisutch* | present | XP_020334279.2 | 271 | zfCW-PWWP | zinc finger CW-type PWWP domain protein 2 |
|  | *Dicentrarchus labrax* | not found (c) | NA | NA | NA | NA |
| ***Tex15*** | *Salmo salar* | present | XP_045549530.1 | 2702 | DUF3715-TEX15 | uncharacterized protein LOC123726301 isoform X1 |
|  | *Oncorhynchus mykiss* | present | XP_036832662 | 2546 | DUF3715-TEX15 | uncharacterized protein LOC110523183 isoform X1 |
|  | *Oncorhynchus kisutch* | present | XP_020313884 | 2576 | DUF3715-TEX15 | testis-expressed protein 15 |
|  | *Dicentrarchus labrax* | not found (c) | NA | NA | NA | NA |
| ***Fbxo47*** | *Salmo salar* | present | XP_014029416 | 446 | F-BOX | PREDICTED: F-box only protein 47 |
|  | *Oncorhynchus mykiss* | present | XP_021438879 | 447 | F-BOX | F-box only protein 47 |
|  | *Oncorhynchus kisutch* | present | XP_020355253 | 446 | F-BOX | F-box only protein 47 |
|  | *Dicentrarchus labrax* | present | XP_051259065 | 445 | F-BOX | F-box only protein 47-like |

(a) The original RefSeq protein (XP_031688551.1) is partial (493 AA), due to a frameshift in exon 14 of *Zcwpw1*. The analysis of RNAseq data from *Oncorhynchus kisutch* (SRX4998080) showed that this frameshift is not present among RNAseq reads covering exon 14 (N=89 sequence reads), which suggests that the frameshift corresponds to an error in the genome assembly. After correction of this frameshift, the genome encodes a full-length protein (765 AA).

(b) There is one weak TBLASTN hit on scaffold NW_026136702 (genome assembly GCF_905237075.1), but it corresponds to another protein containing a PWWP domain (Msh6: XP_051275326).

(c) No TBLASTN hit on genome assembly (GCF_905237075.1)
