# Supplementary material for: PRDM9 drives the location and rapid evolution of recombination hotspots in salmonid fish
Source: PLoS Biol. 2025 Jan 6;23(1):e3002950. doi: 10.1371/journal.pbio.3002950 (PMC11703093; doi:10.1371/journal.pbio.3002950)
Supplement: S7 Table — Details about the Salmo salar and Oncorhynchus mykiss samples used in this study and the corresponding Prdm9ɑ genotypes identified. (DOCX) [file pbio.3002950.s009.docx]

**S7 Table: List of genotyped samples.** Details about the *Salmo salar* and *Oncorhynchus mykiss* samples used in this study and the corresponding *Prdm9ɑ* genotypes identified.

| **Sample** | **Sample location** | **PRDM9ɑ genotypes** | |
| --- | --- | --- | --- |
| ***Salmo salar*** |  | ***α1.a.2*** | ***α2.2*** |
| ssa12816 | l’Oir / la Sélune river | allele 3 / allele 4 | allele 1 / allele 1 |
| ssa12805 | l’Oir / la Sélune river | allele 1 / allele 2 | allele 1 / allele 1 |
| ssa13740 | l’Oir / la Sélune river | allele 3 / allele 3 | allele 1 / allele 1 |
| ssa13649 | l’Oir / la Sélune river | allele 1 / allele 4 | allele 1 / allele 1 |
| ssa13672 | l’Oir / la Sélune river | allele 1 / allele 3 | allele 1 / allele 1 |
| ssa13651 | l’Oir / la Sélune river | allele 3 / allele 3 | allele 1 / allele 1 |
| ssa13652 | l’Oir / la Sélune river | allele 1 / allele 3 | allele 1 / allele 1 |
| ssa13653 | l’Oir / la Sélune river | allele 5 / allele 6 | allele 1 / allele 1 |
| ssa13654 | l’Oir / la Sélune river | allele 3 / allele 7 | allele 1 / allele 1 |
| ssa13655 | l’Oir / la Sélune river | allele 1 / allele 6 | allele 1 / allele 1 |
| ssa13656 | l’Oir / la Sélune river | allele 1 / allele 9 | allele 1 / allele 1 |
| ssa13657 | l’Oir / la Sélune river | allele 3 / allele 10 | allele 1 / allele 1 |
| ssa13658 | l’Oir / la Sélune river | allele 1 / allele 8 | allele 1 / allele 1 |
| ssa13659 | l’Oir / la Sélune river | allele 1 / allele 3 | allele 1 / allele 1 |
| ssa13661 | l’Oir / la Sélune river | allele 3 / allele 3 | allele 1 / allele 1 |
| ssa13662 | l’Oir / la Sélune river | allele 3 / allele 6 | allele 1 / allele 1 |
| ssa13663 | l’Oir / la Sélune river | allele 1 / allele 3 | allele 1 / allele 1 |
| ssa13664 | l’Oir / la Sélune river | allele 1 / allele 9 | allele 1 / allele 1 |
| ssa13668 | l’Oir / la Sélune river | allele 3 / allele 5 | allele 1 / allele 1 |
| ssa13669 | l’Oir / la Sélune river | allele 1 / allele 3 | allele 1 / allele 1 |
| PIT-475 | l’Oir / la Sélune river | allele 3 / allele 9 |  |
| PIT-693 | l’Oir / la Sélune river | allele 3 / allele 3 |  |
| PIT-948 | l’Oir / la Sélune river | allele 6 / allele 6 |  |
| ssa-261 | l’Oir / la Sélune river | allele 1 / allele 11 |  |
| ssa-673 | l’Oir / la Sélune river | allele 1 / allele 1 |  |
| ssa-728 | l’Oir / la Sélune river | allele 3 / allele 3 |  |
| ***Oncorhynchus mykiss*** | | ***α1.a.1*** | ***α2.2*** |
| rt601 | INRAE PEIMA (Sizun) | allele 1 / allele 6 | allele 1 / allele 1 |
| rt602 | INRAE PEIMA (Sizun) | allele 2 / allele 4 | allele 1 / allele 1 |
| rt603 | INRAE PEIMA (Sizun) | allele 2 / allele 6 | allele 2 / allele 2 |
| rt604 | INRAE PEIMA (Sizun) | allele 3 / allele 5 | allele 1 / allele 1 |
| rt605 | INRAE PEIMA (Sizun) | allele 1 / allele 3 | allele 1 / allele 4 |
| rt606 | INRAE PEIMA (Sizun) | allele 4 / allele 4 | allele 1 / allele 3 |
| rt607 | INRAE PEIMA (Sizun) | allele 4 / allele 6 | allele 1 / allele 5 |
| rt608 | INRAE PEIMA (Sizun) | allele 4 / allele 5 | allele 1 / allele 3 |
| rt609 | INRAE PEIMA (Sizun) | allele 4 / allele 4 | allele 1 / allele 1 |
| rt610 | INRAE PEIMA (Sizun) | allele 2 / allele 6 | allele 2 / allele 2 |
| rt611 | INRAE PEIMA (Sizun) | allele 5 / allele 6 | allele 1 / allele 3 |
| rt612 | INRAE PEIMA (Sizun) | allele 4 / allele 4 | allele 1 / allele 2 |
| rt613 | INRAE PEIMA (Sizun) | allele 4 / allele 7 | allele 1 / allele 2 |
| rt614 | INRAE PEIMA (Sizun) | allele 6 / allele 6 | allele 3 / allele 3 |
| rt615 | INRAE PEIMA (Sizun) | allele 2 / allele 6 | allele 1 / allele 1 |
| rt616 | INRAE PEIMA (Sizun) | allele 4 / allele 5 | allele 1 / allele 3 |
| rt617 | INRAE PEIMA (Sizun) | allele 4 / allele 6 | allele 1 / allele 1 |
| rt618 | INRAE PEIMA (Sizun) | allele 4 / allele 4 | allele 1 / allele 3 |
| rt619 | INRAE PEIMA (Sizun) | allele 4 / allele 4 | allele 1 / allele 1 |
| rt620 | INRAE PEIMA (Sizun) | allele 2 / allele 6 | allele 1 / allele 3 |
| RT-52 | INRAE PEIMA (Sizun) | allele 1 / allele 2 |  |
| TAC-1 | INRAE PEIMA (Sizun) | allele 1 / allele 5 |  |
| TAC-3 | INRAE PEIMA (Sizun) | allele 2 / allele 6 |  |
