# Supplementary material for: PRDM9 drives the location and rapid evolution of recombination hotspots in salmonid fish
Source: PLoS Biol. 2025 Jan 6;23(1):e3002950. doi: 10.1371/journal.pbio.3002950 (PMC11703093; doi:10.1371/journal.pbio.3002950)
Supplement: S8 Table — The primers used in this study to genotype the zinc finger array of Prdm9ɑ in Salmo salar and Oncorhynchus mykiss. (DOCX) [file pbio.3002950.s010.docx]

**S8 Table: List of primers.** The primers used in this study to genotype the zinc finger array of *Prdm9ɑ* in *Salmo salar* and *Oncorhynchus mykiss*.

| **Primer ID** | **Sequence (5'->3')** | **Target paralog** | **Use** |
| --- | --- | --- | --- |
| ***Salmo salar*** | | | |
| ssa05Pa2F | TGTTGAGGAGTGGAGAGATCAGA | *α1.a.2* | PCR and sequencing |
| ssa05Pa2R | CCCGCCCAGTTAGGCTTCTA | *α1.a.2* | PCR |
| ssa05Pa3R | AACAGTTAGTTGAAAGTTCCACG | *α1.a.2* | Sequencing |
| ssa17Pa1F | TGTTCTCCTTCACGGCTCAG | *α2.2* | PCR |
| ssa17Pa2R | TGCACTGTCTTTGGGGCTGATTA | *α2.2* | PCR |
| ssa17Pa3F | GTGGCTCTCAACGATGTTCAA | *α2.2* | Sequencing |
| ssa17Pa3R | CCAGTAAATCAGTTGGTGCTA | *α2.2* | Sequencing |
| ***Oncorhynchus mykiss*** | | | |
| rt31Pa1F_new | CTCTGGCTGTCCGTTCTCCTTCACC | *α1.a.1* | PCR |
| rt31Pa2R_new | GACTGCAGTTGGTGTGGGTACTGG | *α1.a.1* | PCR |
| ssa05Pa2F | TGTTGAGGAGTGGAGAGATCAGA | *α1.a.1* | Sequencing |
| rt31Pa1R | ATACACAAGATGGACGCCAGAGT | *α1.a.1* | Sequencing |
| rt07Pa2F | GCTCTCAGCGGTGTTCAACAACT | *α2.2* | PCR and sequencing |
| rt07Pa1R | ACATGTCTCACTGCAGTCACC | *α2.2* | PCR and sequencing |
