# Supplementary material for: PRDM9 drives the location and rapid evolution of recombination hotspots in salmonid fish
Source: PLoS Biol. 2025 Jan 6;23(1):e3002950. doi: 10.1371/journal.pbio.3002950 (PMC11703093; doi:10.1371/journal.pbio.3002950)
Supplement: S9 Table — Details about the ChIP-seq experiment performed in Oncorhynchus mykiss testes. In the third column DMC1-R1 or -GP refer to the animal used to raise the antibody, respectively, rabbit individual 1 and guinea pig. (DOCX) [file pbio.3002950.s011.docx]

**S9 Table: List of ChIP-seq experiments performed.** Details about the ChIP-seq experiment performed in *Oncorhynchus mykiss* testes. In the third column DMC1-R1 or -GP refer to the animal used to raise the antibody, respectively rabbit individual 1 and guinea pig.

| **Sample** | **Maturation stage** | **Antibody** | **Experiment** | **Replicate** | **Raw reads** | **Mapped reads** | **Accession number** |
| --- | --- | --- | --- | --- | --- | --- | --- |
| RT-52 | IV | DMC1 - R1 | ChIP-seq | 1 | 110M | 70M | GSM8522616 |
| RT-52 | IV | DMC1 - GP | ChIP-seq | 2 | 94M | 69M | GSM8522617 |
| RT-52 | IV | None | Input | 1 | 105M | 90M | GSM8522618 |
| TAC-1 | III | DMC1 - R1 | ChIP-seq | 1 | 87M | 64M | GSM8522619 |
| TAC-1 | III | DMC1 - R1 | ChIP-seq | 2 | 100M | 78M | GSM8522620 |
| TAC-1 | III | H3K4me3 | ChIP-seq | 1 | 486M | 461M | GSM8522629 |
| TAC-1 | III | H3K4me3 | ChIP-seq | 2 | 400M | 371M | GSM8522631 |
| TAC-1 | III | H3K36me3 | ChIP-seq | 1 | 555M | 524M | GSM8522630 |
| TAC-1 | III | H3K36me3 | ChIP-seq | 2 | 394M | 370M | GSM8522632 |
| TAC-1 | III | None | Input | 1 | 518M | 509M | GSM8522633 |
| TAC-3 | III | DMC1 - R1 | ChIP-seq | 1 | 91M | 68M | GSM8522621 |
| TAC-3 | III | DMC1 - R1 | ChIP-seq | 2 | 67M | 51M | GSM8522622 |
| TAC-3 | III | H3K4me3 | ChIP-seq | 1 | 229M | 210M | GSM8522623 |
| TAC-3 | III | H3K4me3 | ChIP-seq | 2 | 472M | 456M | GSM8522626 |
| TAC-3 | III | H3K36me3 | ChIP-seq | 1 | 251M | 229M | GSM8522624 |
| TAC-3 | III | H3K36me3 | ChIP-seq | 2 | 449M | 433M | GSM8522627 |
| TAC-3 | III | None | Input | 1 | 327M | 316M | GSM8522625 |
| TAC-3 | III | None | Input | 2 | 536M | 528M | GSM8522628 |
