# Supplementary material for: PRDM9 drives the location and rapid evolution of recombination hotspots in salmonid fish
Source: PLoS Biol. 2025 Jan 6;23(1):e3002950. doi: 10.1371/journal.pbio.3002950 (PMC11703093; doi:10.1371/journal.pbio.3002950)
Supplement: S10 Table — Population samples of O. kisutch, O. mykiss, and S. salar used to build the linkage disequilibrium-based recombination landscapes were selected from [118–120]. (DOCX) [file pbio.3002950.s012.docx]

**S10 Table:** **Sample accession number and location.** Population samples of *O. kisutch*, *O. mykiss* and *S. salar* used to build the linkage disequilibrium-based recombination landscapes were selected from (1-3).

| **Sample** | **SRA accession number** | **Sample location** |
| --- | --- | --- |
| ***Oncorhynchus kisutch*** | | |
| Okis_ca_01 | SRX3161578 | Capilano River Hatchery |
| Okis_ca_02 | SRX3161579 | Capilano River Hatchery |
| Okis_ca_03 | SRX3161580 | Capilano River Hatchery |
| Okis_ca_04 | SRX3161581 | Capilano River Hatchery |
| Okis_ca_05 | SRX3161582 | Capilano River Hatchery |
| Okis_ic_01 | SRX3161545 | Inch Creek Hatchery |
| Okis_ic_02 | SRX3161544 | Inch Creek Hatchery |
| Okis_ic_03 | SRX3161543 | Inch Creek Hatchery |
| Okis_ic_04 | SRX3161547 | Inch Creek Hatchery |
| Okis_ic_05 | SRX3161546 | Inch Creek Hatchery |
| Okis_ro_01 | SRX3161552 | Robertson Creak Hatchery |
| Okis_ro_02 | SRX3161555 | Robertson Creak Hatchery |
| Okis_ro_03 | SRX3161554 | Robertson Creak Hatchery |
| Okis_ro_04 | SRX3161557 | Robertson Creak Hatchery |
| Okis_ro_05 | SRX3161556 | Robertson Creak Hatchery |
| Okis_sa_01 | SRX3161583 | Salmon River |
| Okis_sa_02 | SRX3161584 | Salmon River |
| Okis_sa_03 | SRX3161585 | Salmon River |
| Okis_sa_04 | SRX3161575 | Salmon River |
| Okis_sa_06 | SRX3161576 | Salmon River |
| **Oncorhynchus mykiss** | | |
| Omyk_dw_01 | SRX2820108 | Dworshak |
| Omyk_dw_02 | SRX2820111 | Dworshak |
| Omyk_dw_03 | SRX2820105 | Dworshak |
| Omyk_dw_04 | SRX2820113 | Dworshak |
| Omyk_el_01 | SRX2820112 | Elwha |
| Omyk_el_02 | SRX2820103 | Elwha |
| Omyk_el_03 | SRX2820102 | Elwha |
| Omyk_el_04 | SRX2820071 | Elwha |
| Omyk_id_01 | SRX2820064 | Idaho (Big Bear River) |
| Omyk_id_02 | SRX2820079 | Idaho (Big Bear River) |
| Omyk_lq_01 | SRX2820073 | L. Quinault |
| Omyk_lq_02 | SRX2820107 | L. Quinault |
| Omyk_lq_03 | SRX2820106 | L. Quinault |
| Omyk_lq_04 | SRX2820084 | L. Quinault |
| Omyk_qu_01 | SRX2820093 | Quinault |
| Omyk_qu_02 | SRX2820085 | Quinault |
| Omyk_qu_03 | SRX2820087 | Quinault |
| Omyk_qu_04 | SRX2820104 | Quinault |
| Omyk_sk_01 | SRX2820109 | Skamania |
| Omyk_sk_02 | SRX2820098 | Skamania |
| Omyk_sk_03 | SRX2820101 | Skamania |
| Omyk_sk_04 | SRX2820072 | Skamania |
| ***Salmo Salar*** | | |
| Ssal_gp_01 | ERS4601709 | Bonaventure |
| Ssal_gp_02 | ERS4601710 | Bonaventure |
| Ssal_gp_03 | ERS4601714 | Bonaventure |
| Ssal_gp_04 | ERS4601715 | Bonaventure |
| Ssal_gp_05 | ERS4601716 | Bonaventure |
| Ssal_gp_06 | ERS4601717 | Bonaventure |
| Ssal_gp_07 | ERS4601718 | Bonaventure |
| Ssal_gp_08 | ERS4601977 | Petite riviere Cascapedia |
| Ssal_gp_09 | ERS4601978 | Petite riviere Cascapedia |
| Ssal_gp_10 | ERS4601979 | Petite riviere Cascapedia |
| Ssal_gp_11 | ERS4601980 | Petite riviere Cascapedia |
| Ssal_gp_12 | ERS4601981 | Petite riviere Cascapedia |
| Ssal_gp_13 | ERS4601983 | Petite riviere Cascapedia |
| Ssal_gp_14 | ERS4601985 | Petite riviere Cascapedia |
| Ssal_gp_15 | ERS4601986 | Petite riviere Cascapedia |
| Ssal_gp_16 | ERS4601721 | De la Chaloupe |
| Ssal_gp_17 | ERS4601723 | De la Chaloupe |
| Ssal_gp_18 | ERS4601724 | De la Chaloupe |
| Ssal_gp_19 | ERS4601725 | De la Chaloupe |
| Ssal_gp_20 | ERS4601726 | De la Chaloupe |
| Ssal_bs_01 | ERS4601833 | Komagelva |
| Ssal_bs_02 | ERS4601834 | Komagelva |
| Ssal_bs_03 | ERS4601835 | Komagelva |
| Ssal_bs_04 | ERS4601836 | Komagelva |
| Ssal_bs_05 | ERS4601837 | Komagelva |
| Ssal_bs_06 | ERS4601839 | Komagelva |
| Ssal_bs_07 | ERS4601842 | Komagelva |
| Ssal_bs_08 | ERS4601935 | Neiden |
| Ssal_bs_09 | ERS4601936 | Neiden |
| Ssal_bs_10 | ERS4601938 | Neiden |
| Ssal_bs_11 | ERS4601939 | Neiden |
| Ssal_bs_12 | ERS4601941 | Neiden |
| Ssal_bs_13 | ERS4601942 | Neiden |
| Ssal_bs_14 | ERS4601943 | Neiden |
| Ssal_bs_15 | ERS4601944 | Neiden |
| Ssal_bs_16 | ERS4601862 | Langfjordvassdraget |
| Ssal_bs_17 | ERS4601865 | Langfjordvassdraget |
| Ssal_bs_18 | ERS4601866 | Langfjordvassdraget |
| Ssal_bs_19 | ERS4601867 | Langfjordvassdraget |
| Ssal_bs_20 | ERS4601869 | Langfjordvassdraget |
| Ssal_ns_01 | ERS4602054 | Suldalslaagen |
| Ssal_ns_02 | ERS4602056 | Suldalslaagen |
| Ssal_ns_03 | ERS4602057 | Suldalslaagen |
| Ssal_ns_04 | ERS4602058 | Suldalslaagen |
| Ssal_ns_05 | ERS4602059 | Suldalslaagen |
| Ssal_ns_06 | ERS4602060 | Suldalslaagen |
| Ssal_ns_07 | ERS4602061 | Suldalslaagen |
| Ssal_ns_08 | ERS4602062 | Suldalslaagen |
| Ssal_ns_09 | ERS4602063 | Suldalslaagen |
| Ssal_ns_10 | ERS4602090 | Vikedalselva i Vindafjord |
| Ssal_ns_11 | ERS4602091 | Vikedalselva i Vindafjord |
| Ssal_ns_12 | ERS4602092 | Vikedalselva i Vindafjord |
| Ssal_ns_13 | ERS4602093 | Vikedalselva i Vindafjord |
| Ssal_ns_14 | ERS4602094 | Vikedalselva i Vindafjord |
| Ssal_ns_15 | ERS4602097 | Vikedalselva i Vindafjord |
| Ssal_ns_16 | ERS4602098 | Vikedalselva i Vindafjord |
| Ssal_ns_17 | ERS4602099 | Vikedalselva i Vindafjord |
| Ssal_ns_18 | ERS4601778 | Flaamselva |
| Ssal_ns_19 | ERS4601780 | Flaamselva |
| Ssal_ns_20 | ERS4601784 | Flaamselva |

**References**

1. Bertolotti AC, Layer RM, Gundappa MK, Gallagher MD, Pehlivanoglu E, Nome T, et al. The structural variation landscape in 492 Atlantic salmon genomes. Nat Commun. 2020;11(1):5176.

2. Gao G, Nome T, Pearse DE, Moen T, Naish KA, Thorgaard GH, et al. A New Single Nucleotide Polymorphism Database for Rainbow Trout Generated Through Whole Genome Resequencing. Front Genet. 2018;9:147.

3. Rondeau EB, Christensen KA, Minkley DR, Leong JS, Chan MTT, Despins CA, et al. Population-size history inferences from the coho salmon (Oncorhynchus kisutch) genome. G3 (Bethesda). 2023;13(4).
