# Supplementary material for: PRDM9 drives the location and rapid evolution of recombination hotspots in salmonid fish
Source: PLoS Biol. 2025 Jan 6;23(1):e3002950. doi: 10.1371/journal.pbio.3002950 (PMC11703093; doi:10.1371/journal.pbio.3002950)
Supplement: S11 Table — Details of the program versions used at each step of the reconstruction of LD-based recombination landscapes in the 5 salmonid populations. * The D. labrax data set was taken from [128], who used a reference panel of 22 genomes fully phased-by-transmission using trio-sequencing as a learning reference for the statistical phasing of 46 additional genomes with Eagle2 v2.4. Variants were oriented using whole-genome resequencing data (>20×) from the closely related species Dicentrarchus punctatus, which was used as an outgroup. (DOCX) [file pbio.3002950.s013.docx]

**S11 Table: Program versions.** Details of the program versions used at each step of the reconstruction of LD-based recombination landscapes in the five salmonid populations. * The *D. labrax* dataset was taken from (1), who used a reference panel of 22 genomes fully phased-by-transmission using trio-sequencing as a learning reference for the statistical phasing of 46 additional genomes with Eagle2 v2.4. Variants were oriented using whole-genome resequencing data (>20x) from the closely related species *Dicentrarchus punctatus*, which was used as an outgroup.

| Steps | Read mapping | BAM formating | Variant calling | Variant Filtering | Variant Filtering | Pre- phasing | Phasing | Variant orientation | Recombination rates estimation | TEs de novo annotation | TEs mapping | CGIs annotation |
| --- | --- | --- | --- | --- | --- | --- | --- | --- | --- | --- | --- | --- |
| Programs | bwa mem | Picard | GATK | Bcftools | VCFtools | WhatsHap | Shapeit | Est-sfs | LDhelmet | Repeat Modeler | Repeat Masker | EMBOSS |
| *O. kisutch* | v0.7.17 | v2.25.6 | v3.8-0 (2) | 1.9 | v 0.1.16 | 0.18 | 4.2.1 | 2.03 | 1.9 | 2.03 | 4.1.3 | 6.6.0 |
| *O. mykiss* | v0.7.17 | v2.25.6 | v4.2.2.0 | 1.9 | v 0.1.16 | 0.18 | 4.2.1 | 2.03 | 1.9 | 2.03 | 4.1.3 | 6.6.0 |
| *S. salar* GP | v0.7.17 | v2.18.29 | 4.1.8.1 | 1.9 | 0.1.17 | 1.3 | 4.2.2 | 2.04 | 1.9 | 2.03 | 4.1.3 | 6.6.0 |
| *S. salar* BS | v0.7.17 | v2.18.29 | 4.1.8.1 | 1.9 | 0.1.17 | 1.3 | 4.2.2 | 2.04 | 1.9 | 2.03 | 4.1.3 | 6.6.0 |
| *S. salar* NS | v0.7.17 | v2.18.29 | 4.1.8.1 | 1.9 | 0.1.17 | 1.3 | 4.2.2 | 2.04 | 1.9 | 2.03 | 4.1.3 | 6.6.0 |
| *D. labrax* | v0.7.5a | v1.112 | v3.3-0 (1) | - | v0.1.11 |  | * | - | v1.10 | - | - | 6.6.0 |

**References**

1. Duranton M, Allal F, Valière S, Bouchez O, Bonhomme F, Gagnaire PA. The contribution of ancient admixture to reproductive isolation between European sea bass lineages. Evol Lett. 2020;4(3):226-42.

2. Rondeau EB, Christensen KA, Minkley DR, Leong JS, Chan MTT, Despins CA, et al. Population-size history inferences from the coho salmon (Oncorhynchus kisutch) genome. G3 (Bethesda). 2023;13(4).
