# Supplementary material for: PRDM9 drives the location and rapid evolution of recombination hotspots in salmonid fish
Source: PLoS Biol. 2025 Jan 6;23(1):e3002950. doi: 10.1371/journal.pbio.3002950 (PMC11703093; doi:10.1371/journal.pbio.3002950)
Supplement: S12 Table — (DOCX) [file pbio.3002950.s014.docx]

**S12 Table.** Assembly statistics of the reference genome of *O. kisutch*, *O. mykiss* and *S. salar* that were used to map the population resequencing data for the LD-based recombination landscapes and the ChIP-Seq DMC1 peaks.

|  | ***O. kisutch*** | ***O. mykiss*** | | ***S. salar*** |
| --- | --- | --- | --- | --- |
| Reference genome | Okis_V1 | Omyk_1 | USDA_OmykA_1.1 | Ssal_v3 |
| Accession number | GCF_002021735.1 | GCF_002163495.1 | GCF_013265735.2 | GCF_905237065.1 |
| Year | 2017 | 2017 | 2020 | 2021 |
| Genome size | 2.4 Gb | 2.2 Gb | 2.3 Gb | 2.8 Gb |
| Total ungapped length | 2.3 Gb | 1.9 Gb | 2.3 Gb | 2.8 Gb |
| Assembly level | Chromosome | Chromosome | Chromosome | Chromosome |
| Number of chromosomes | 30 | 29 | 32 | 29 |
| Number of scaffolds | 22,812 | 139,799 | 938 | 4,223 |
| Scaffold N50 | 1.3 Mb | 1.7 Mb | 39.2 Mb | 28.1 Mb |
| Gaps between scaffolds | 1,566 | 7,839 | 196 | 212 |
| Genome coverage | 213.0x | 244.0x | 111.6x | 70.0x |
