# Supplementary material for: PRDM9 drives the location and rapid evolution of recombination hotspots in salmonid fish
Source: PLoS Biol. 2025 Jan 6;23(1):e3002950. doi: 10.1371/journal.pbio.3002950 (PMC11703093; doi:10.1371/journal.pbio.3002950)
Supplement: S13 Table — Outgroups 1, 2, and 3 for O. kisutch were O. tshawytscha, O. nerka, and O. mykiss, respectively; O. tschawytscha, O. nerka, and O. kisutch for O. mykiss; and Salmo trutta, Salvelinus alpinus, and O. mykiss for S. salar. (DOCX) [file pbio.3002950.s015.docx]

**S13 Table: Mean sequence identity score obtained from the blast search of the 100 kb flanking sequences of the variants in the ingroup species against the reference genome of each outgroup.** Outgroups 1, 2 and 3 for *O. kisutch* were *O. tshawytscha*, *O. nerka* and *O. mykiss* respectively; *O. tschawytscha*, *O. nerka* and *O. kisutch* for *O. mykiss*; and *Salmo trutta*, *Salvelinus alpinus* and *O. mykiss* for *S. salar*.

|  | **Outgroup 1** | **Outgroup 2** | **Outgroup 3** |
| --- | --- | --- | --- |
| *O. kisutch* | 96.53 | 95.71 | 95.48 |
| *O. mykiss* | 95.76 | 95.64 | 95.62 |
| *S. salar* | 96.95 | 93.95 | 93.31 |
