# Supplementary material for: PRDM9 drives the location and rapid evolution of recombination hotspots in salmonid fish
Source: PLoS Biol. 2025 Jan 6;23(1):e3002950. doi: 10.1371/journal.pbio.3002950 (PMC11703093; doi:10.1371/journal.pbio.3002950)
Supplement: S1 Fig — The phylogenetic tree was realized on the concatenated 3 exons of the SET domain, with 1,000 bootstrap replicates (values shown at nodes). To facilitate visualization, the branch of D. labrax is not drawn to scale. In column from left to right, (i) species; (ii) annotated paralog copy; (iii) Prdm9 copy status. The scale bar is in unit of substitution per site. The right panel shows the coding potential of each paralog and indicates the presence of substitutions in the catalytic tyrosines of the SET domain (Y276, Y341, and Y357). Canonical (full length) PRDM9 proteins contain 4 key domains: KRAB (encoded by 2 exons), SSXRD (encoded by 1 exon), SET (encoded by 3 exons), and the ZF array (encoded by 1 exon). Complete exons are shown in blue. Missing or truncated exons are shown in pink. Other regions of the protein (upstream of the KRAB domain, and between KRAB and SSXRD), are encoded by additional exons (not shown here), that are not conserved between α and β clades. All β copies have lost KRAB and SSXRD domains, and have substitutions in at least two of the 3 catalytic tyrosines of the SET domain. β copies are well conserved across all species (including in the ZF array), which indicates that these truncated PRDM9 homologs are under purifying selection, and hence that they have a function. The last column indicates indexes referring to the S1 Table with additional information on the corresponding copy. The data and codes underlying this figure can be found in https://doi.org/10.5281/zenodo.11083953. (DOCX) [file pbio.3002950.s016.docx]

**
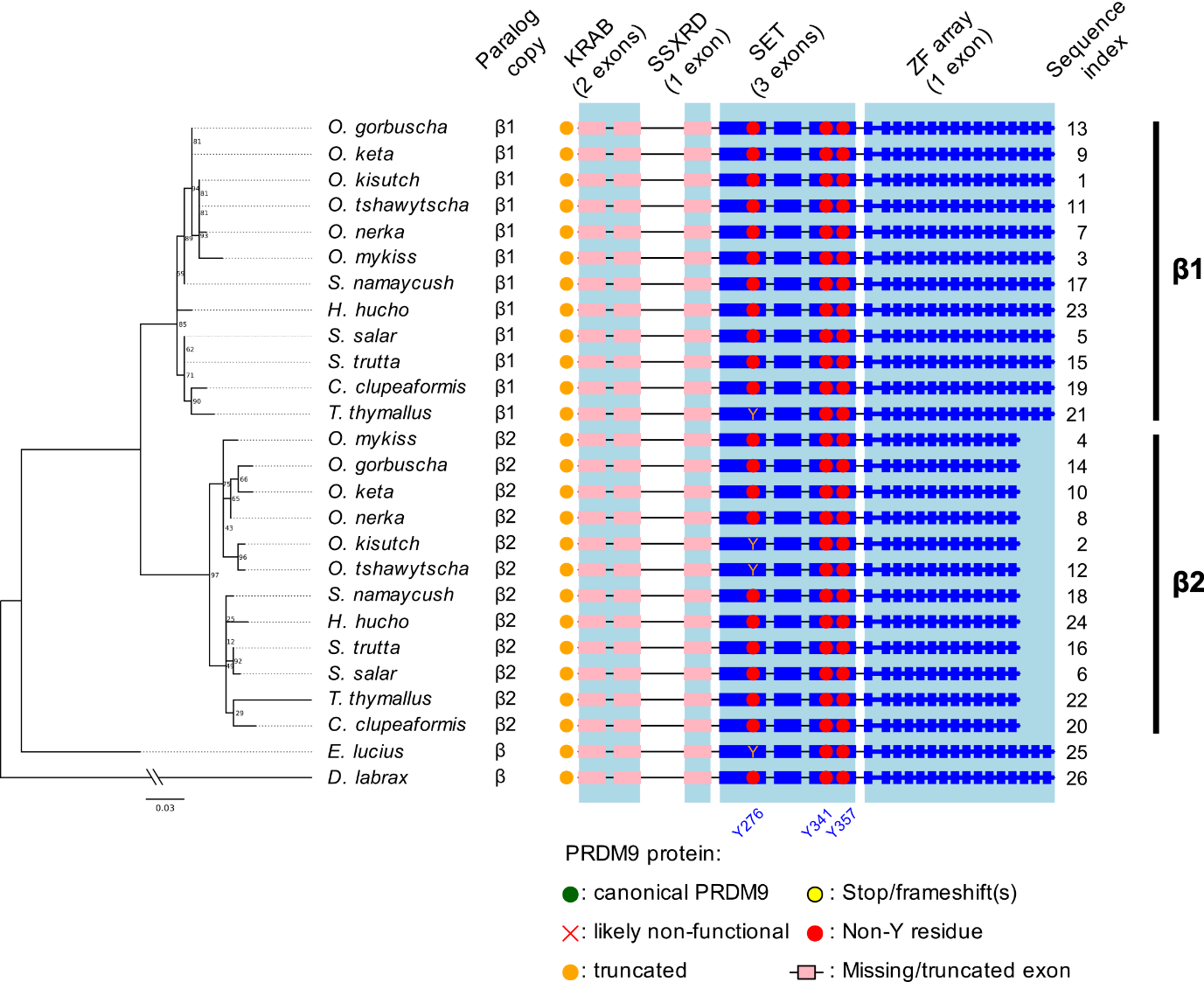
**

**S1 Fig: Phylogenetic distribution of PRDM9β paralogs** in twelve salmonids, the northern pike (*Esox lucius*) and the European sea bass (*Dicentrarchus labrax*) as outgroup species. The phylogenetic tree was realised on the concatenated 3 exons of the SET domain, with 1000 bootstrap replicates (values shown at nodes). To facilitate visualisation, the branch of *D. labrax* is not drawn to scale. In column from left to right, i) species; ii) annotated paralog copy; iii) Prdm9 copy status. The scale bar is in unit of substitution per site. The right panel shows the coding potential of each paralog, and indicates the presence of substitutions in the catalytic tyrosines of the SET domain (Y276, Y341 and Y357). Canonical (full-length) PRDM9 proteins contain four key domains: KRAB (encoded by two exons), SSXRD (encoded by one exon), SET (encoded by three exons) and the ZF array (encoded by one exon). Complete exons are shown in blue. Missing or truncated exons are shown in pink. Other regions of the protein (upstream of the KRAB domain, and between KRAB and SSXRD), are encoded by additional exons (not shown here), that are not conserved between α and β clades. All β copies have lost KRAB and SSXRD domains, and have substitutions in at least two of the three catalytic tyrosines of the SET domain. β copies are well conserved across all species (including in the ZF array), which indicates that these truncated PRDM9 homologs are under purifying selection, and hence that they have a function. The last column indicates indexes referring to the **S1 Table** with additional information on the corresponding copy. The data and codes underlying this figure can be found in https://doi.org/10.5281/zenodo.11083953.
