# Supplementary material for: PRDM9 drives the location and rapid evolution of recombination hotspots in salmonid fish
Source: PLoS Biol. 2025 Jan 6;23(1):e3002950. doi: 10.1371/journal.pbio.3002950 (PMC11703093; doi:10.1371/journal.pbio.3002950)
Supplement: S2 Fig — Relative position and orientation of the a (in red) and b (in blue) tandem duplicated copies of the PRDM9α1.1 and 1.2 paralogs for each species. The chromosome/scaffold name on which the copy seats is shown. α1.1 and 1.2, which occur as single copies in some species, are also shown (in gray). The data underlying this figure can be found in S1 Table. (DOCX) [file pbio.3002950.s017.docx]

**
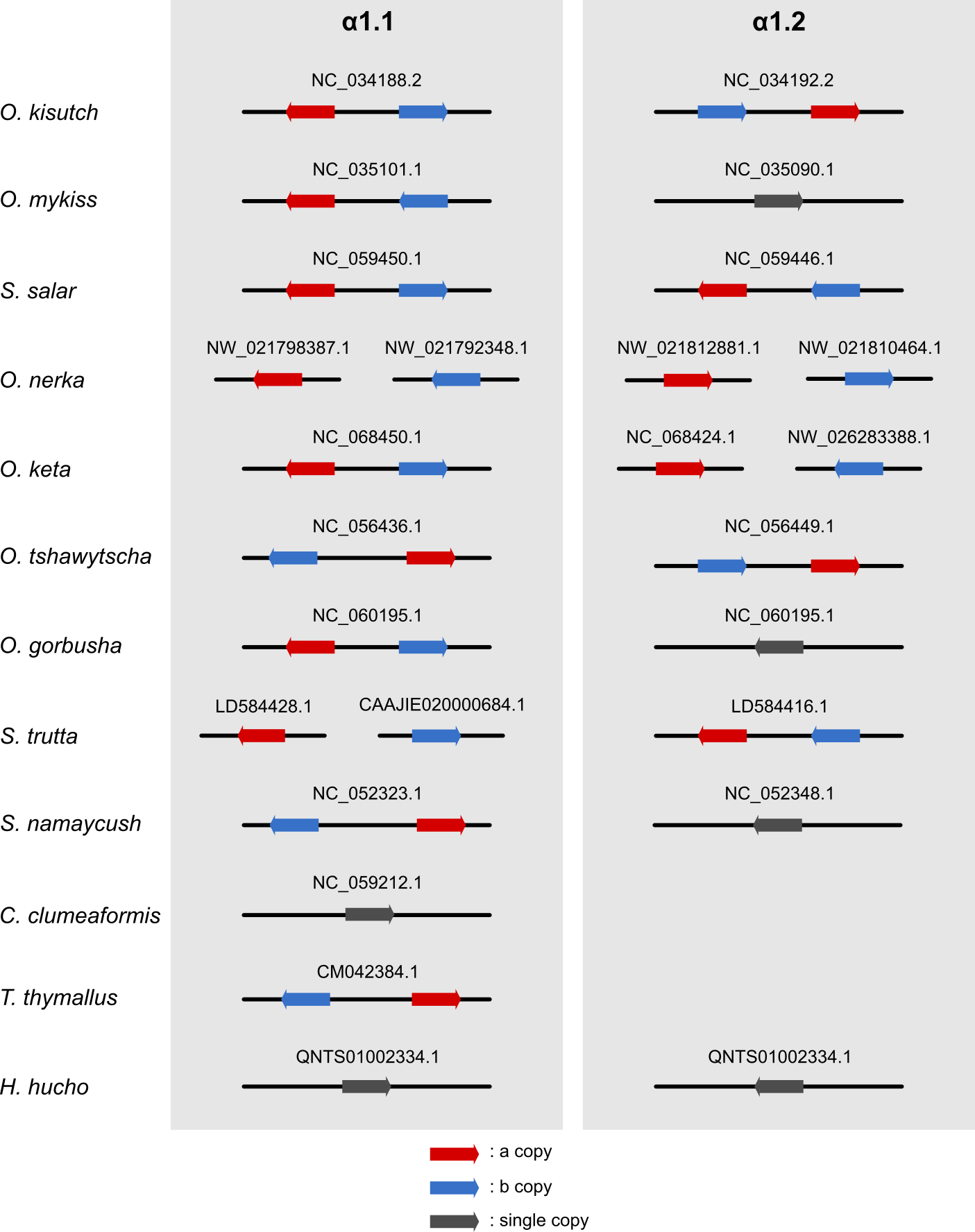
**

**S2 Fig: Chromosome position of the tandem duplicated PRDM9α a and b copies.** Relative position and orientation of the a (in red) and b (in blue) tandem duplicated copies of the PRDM9α1.1 and 1.2 paralogs for each species. The chromosome/scaffold name on which the copy seats is shown. α1.1 and 1.2, which occur as single copies in some species, are also shown (in grey). The data underlying this figure can be found in **S1 Table**.
