# Supplementary material for: PRDM9 drives the location and rapid evolution of recombination hotspots in salmonid fish
Source: PLoS Biol. 2025 Jan 6;23(1):e3002950. doi: 10.1371/journal.pbio.3002950 (PMC11703093; doi:10.1371/journal.pbio.3002950)
Supplement: S3 Fig — Amino acid sequences of all unique zinc fingers found in alleles identified in S. salar PRDM9α1.a.2 and α2.2, and in O. mykiss PRDM9α1.a.1 and α2.2 (Figs 2A and S5). In bold colored boxes are indicated the 3 hypervariable DNA-binding residues. In red are reported the cysteine (C) and histidine (H) residues involved stabilizing the structure of the array. In blue are indicated the polymorphic residues compared to the consensus, outside the 3 amino acids in contact with DNA. In shaded gray are reported the synonym variations in respect to the consensus. The complementary information about the DNA sequences of all alleles identified is available in S1 Methods. (DOCX) [file pbio.3002950.s018.docx]

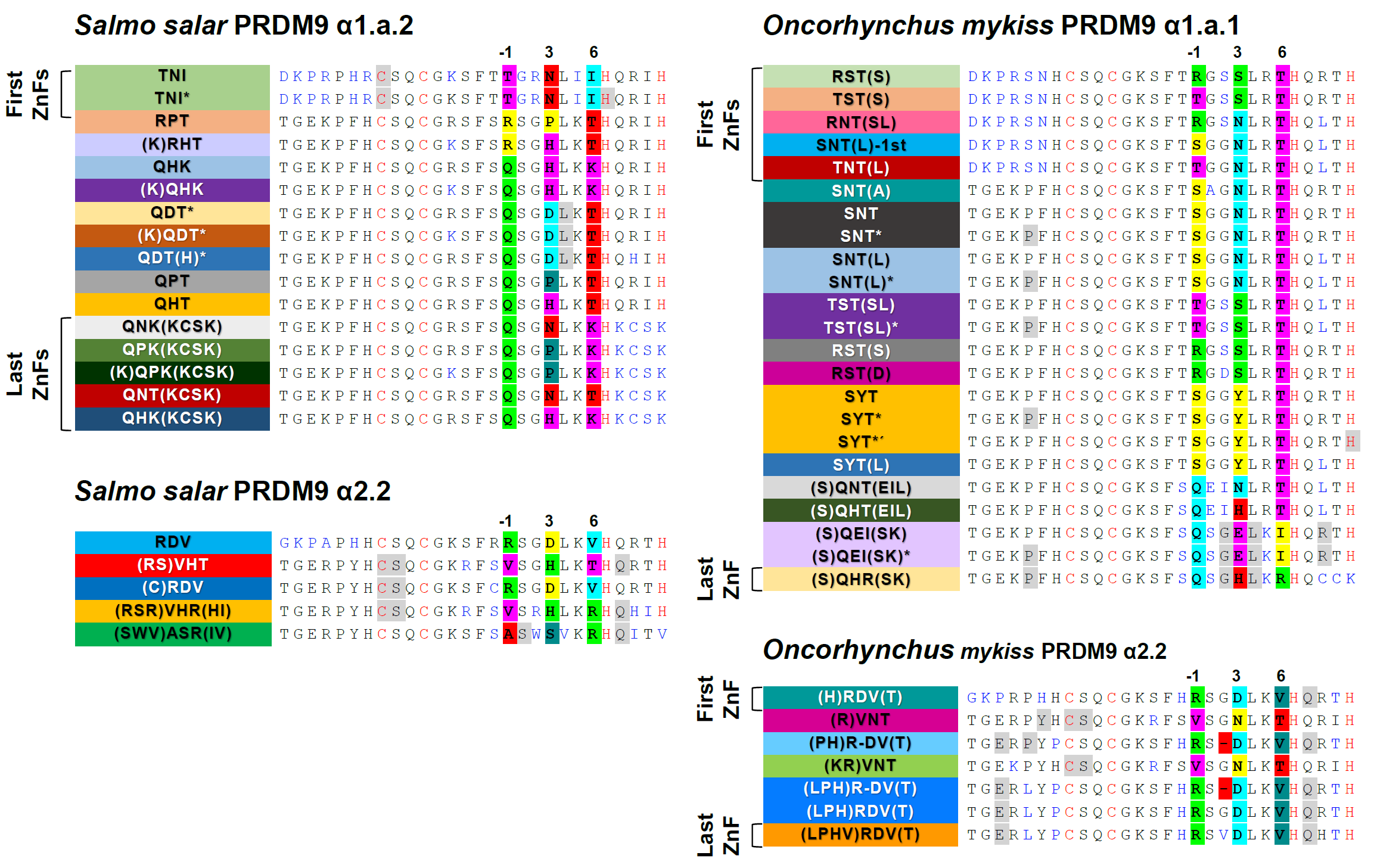


**S3 Fig: Amino-acid diversity in full-length and partial PRDM9 zinc fingers in *S. salar* and *O. mykiss*.** Amino acid sequences of all unique zinc fingers found in alleles identified in *S. salar* PRDM9α1.a.2 and α2.2, and in *O. mykiss* PRDM9α1.a.1 and α2.2 (**Fig 2A**, **S5** **Fig**). In bold colored boxes are indicated the 3 hypervariable DNA-binding residues. In red are reported the cysteine (C) and histidine (H) residues involved stabilizing the structure of the array. In blue are indicated the polymorphic residues compared to the consensus, outside the 3 amino-acids in contact with DNA. In shaded grey are reported the synonym variations in respect to the consensus. The complementary information about the DNA sequences of all alleles identified is available in the **S1 Methods**.
