# Supplementary material for: PRDM9 drives the location and rapid evolution of recombination hotspots in salmonid fish
Source: PLoS Biol. 2025 Jan 6;23(1):e3002950. doi: 10.1371/journal.pbio.3002950 (PMC11703093; doi:10.1371/journal.pbio.3002950)
Supplement: S4 Fig — Cartoon showing the functional domains of PRDM9 paralogs analyzed in this study. The amino acid sequences were obtained from the reference genome and analyzed using previously described methodology [144]. α1 copies and the O. mykiss α2.2 copy possess a complete KRAB domain, and we refer to these copies as canonical PRDM9. S. salar α2.2 copy possess a partial KRAB domain, and we refer to this copy as truncated PRDM9. All 4 copies present the 3 catalytic tyrosine residues in the SET domain, required for methyltransferase activity. (DOCX) [file pbio.3002950.s019.docx]

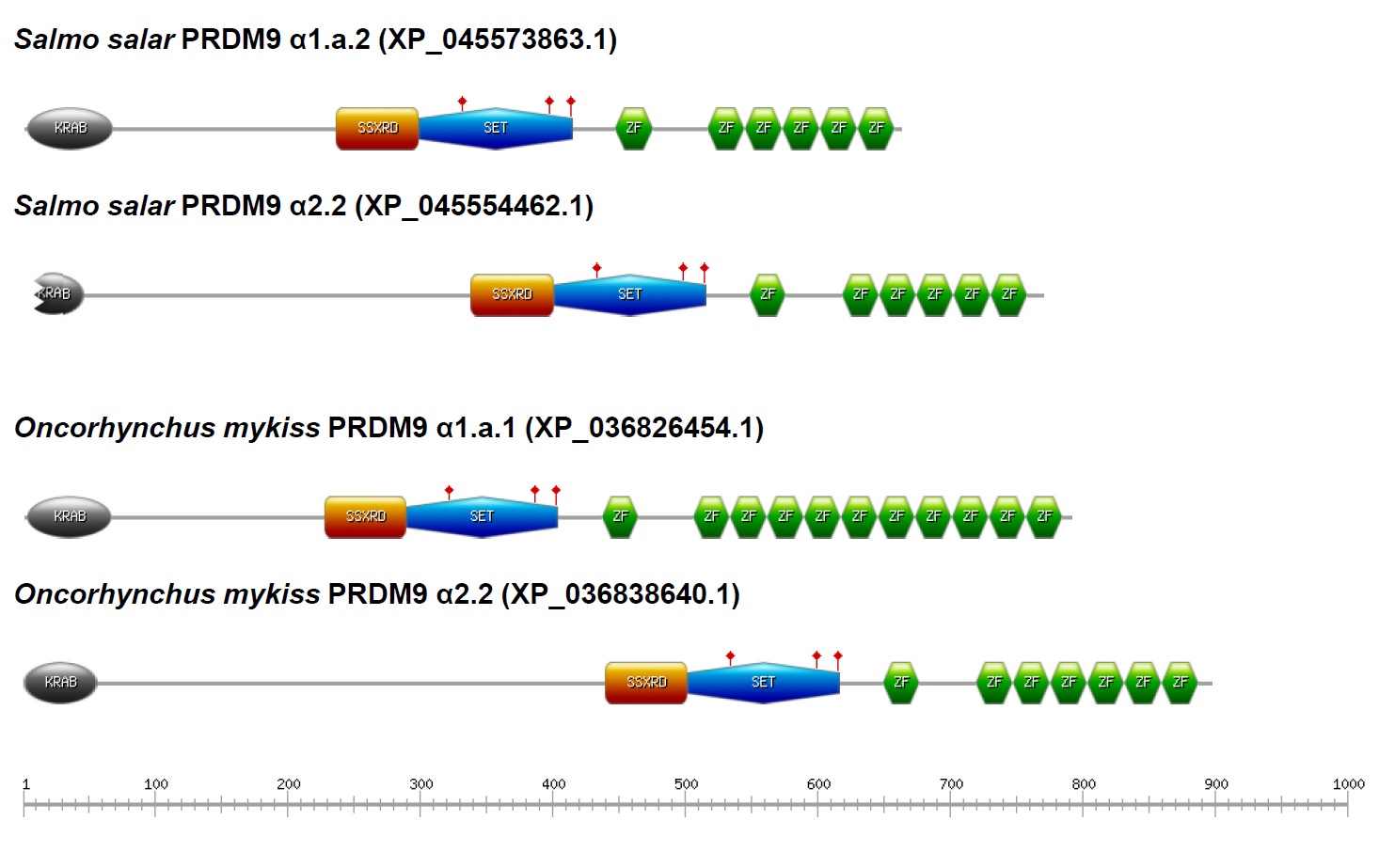


**S4 Fig: Graphical view of PRDM9 paralogs.** Cartoon showing the functional domains of PRDM9 paralogs analyzed in this study. The amino acid sequences were obtained from the reference genome and analyzed using previously described methodology (158). α1 copies and the *O. mykiss* α2.2 copy possess a complete KRAB domain, and we refer to these copies as canonical PRDM9. *S. salar* α2.2 copy possess a partial KRAB domain, and we refer to this copy as truncated PRDM9. All four copies present the three catalytic tyrosine residues in the SET domain, required for methyltransferase activity.
