# Supplementary material for: PRDM9 drives the location and rapid evolution of recombination hotspots in salmonid fish
Source: PLoS Biol. 2025 Jan 6;23(1):e3002950. doi: 10.1371/journal.pbio.3002950 (PMC11703093; doi:10.1371/journal.pbio.3002950)
Supplement: S5 Fig — (A) Structure of PRDM9 zinc finger arrays of identified alleles in S. salar PRDM9α2.2 and O. mykiss PRDM9α2.2. Colored boxes represent unique zinc fingers, characterized by the 3 amino acids in contact with DNA (3-letter code). Additional variations relative to a reference sequence are indicated between brackets. A white star indicates the zinc fingers missing one amino acid residue (27 a.a. instead of 28). The complete zinc finger amino-acid sequences are shown in S3B Fig. Frequencies of the alleles displayed on panel A among the 20 S. salar and 20 O. mykiss individuals that were genotyped for PRDM9. (C) Distribution of amino acid diversity among all unique zinc fingers found in alleles displayed on panel A, following previously described methodology [19]. The amino acid diversity is plotted as a function of amino acid position in the ZF alignment, ranging from position 1 to position 28 (first and last residues) of a ZF unit. The ratio of amino acid diversity at DNA-binding residues of the ZF array (−1, 2, 3, and 6), indicated as r, is shown in the upper box. The data underlying this figure can be found in https://doi.org/10.5281/zenodo.11083953 and in S7 Table. (DOCX) [file pbio.3002950.s020.docx]

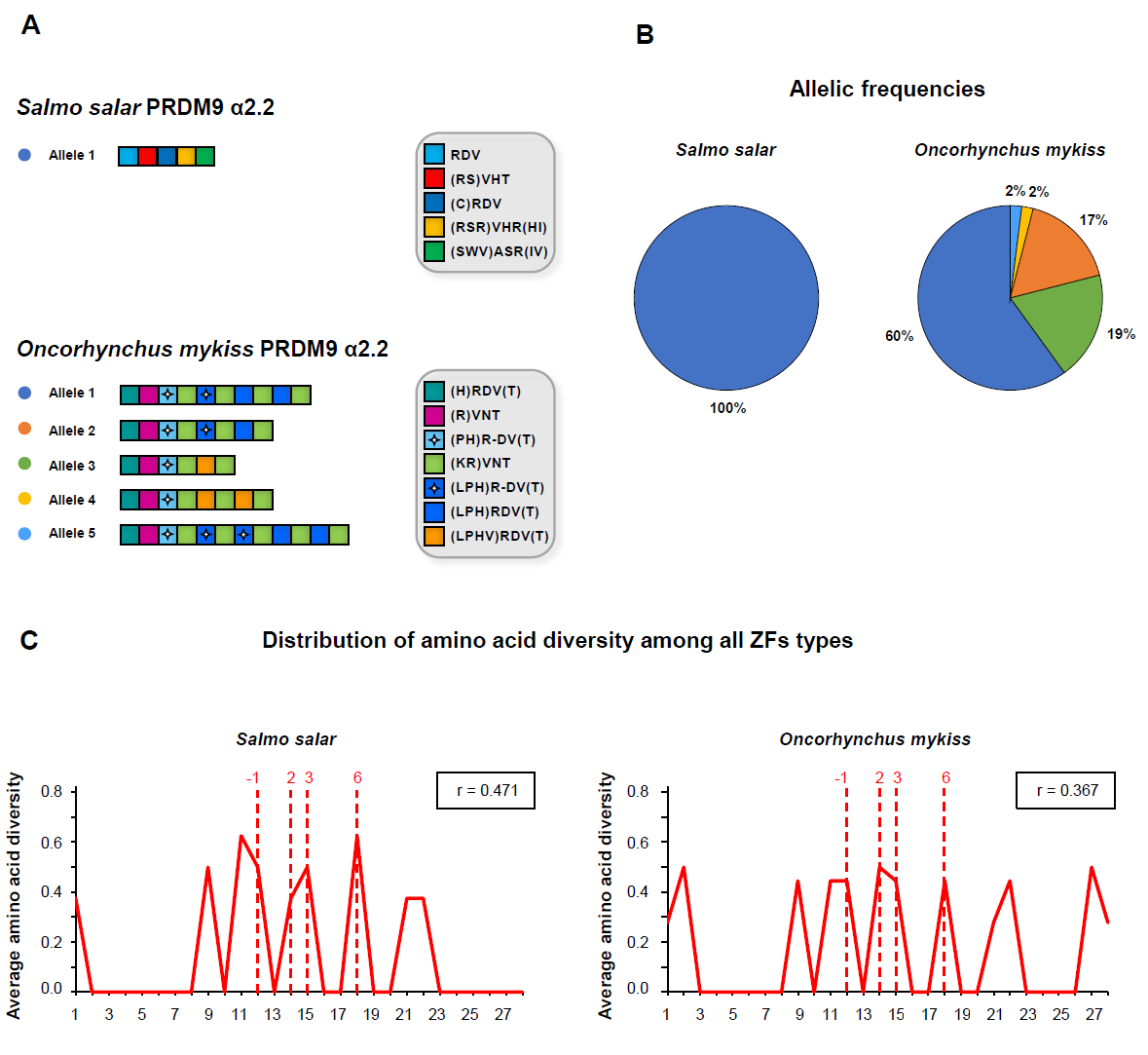


**S5 Fig: PRDM9α2.2 zinc finger allelic diversity in *S. salar* and *O. mykiss*.** **A)** Structure of PRDM9 zinc finger arrays of identified alleles in *S. salar* PRDM9α2.2 and *O. mykiss* PRDM9α2.2. Colored boxes represent unique zinc fingers, characterized by the 3 amino-acids in contact with DNA (3-letter code). Additional variations relative to a reference sequence are indicated between brackets. A white star indicates the zinc fingers missing one amino-acid residue (27 a.a. instead of 28). The complete zinc finger amino-acid sequences are shown in **S3B** **Fig.** Frequencies of the alleles displayed on panel A among the 20 *S. salar* and 20 *O. mykiss* individuals that were genotyped for PRDM9. **C)** Distribution of amino-acid diversity among all unique zinc fingers found in alleles displayed on panel A, following previously described methodology (19). The amino acid diversity is plotted as a function of amino acid position in the ZF alignment, ranging from position 1 to position 28 (first and last residues) of a ZF unit. The ratio of amino acid diversity at DNA-binding residues of the ZF array (-1, 2, 3 and 6), indicated as r, is shown in the upper box. The data underlying this figure can be found in <https://doi.org/10.5281/zenodo.11083953> and in **S7 Table**.
