# Supplementary material for: PRDM9 drives the location and rapid evolution of recombination hotspots in salmonid fish
Source: PLoS Biol. 2025 Jan 6;23(1):e3002950. doi: 10.1371/journal.pbio.3002950 (PMC11703093; doi:10.1371/journal.pbio.3002950)
Supplement: S6 Fig — (A) Average profile of DMC1 ChIP-seq ssDNA fragments orientation (fragments per million, FPM) in TAC-1, TAC-3, and RT-52 testes, at DSB hotspots detected in TAC-1, TAC-3, and RT-52. The profile from each experiment performed is shown (2 replicates/sample). Signal mapped on the forward strand is depicted in blue, signal aligned to the reverse strand is shown in green, as shown in the cartoon on top of the panel. (B) Upset plot showing intersections between DSB hotspots from TAC-1 (n = 616), TAC-3 (n = 209), and RT-52 (n = 1,924). (C) DMC1 ChIP-seq signal fold enrichment (scaled by the average signal in intergenic regions) at multiple genomic features. TSS inside and outside CGIs are highlighted in purple and turquoise, respectively. The data and codes underlying this figure can be found in https://doi.org/10.5281/zenodo.11083953 and https://zenodo.org/records/14198863. (DOCX) [file pbio.3002950.s021.docx]

**S6 Fig: Meiotic DSB hotspots features in *O. mykiss*. A)** Average profile of DMC1 ChIP-seq ssDNA fragments orientation (fragments per million, FPM) in TAC-1, TAC-3 and RT-52 testes, at DSB hotspots detected in TAC-1, TAC-3 and RT-52. The profile from each experiment performed is shown (two replicates/sample). Signal mapped on the forward strand is depicted in blue, signal aligned to the reverse strand is shown in green, as shown in the cartoon on top of the panel. **B)** Upset plot showing intersections between DSB hotspots from TAC-1 (n=616), TAC-3 (n=209) and RT-52 (n=1924). **C)** DMC1 ChIP-seq signal fold enrichment (scaled by the average signal in intergenic regions) at multiple genomic features. TSS inside and outside CGIs are highlighted in purple and turquoise, respectively. The data and codes underlying this figure can be found in <https://doi.org/10.5281/zenodo.11083953> and https://zenodo.org/records/14198863.
