# Supplementary material for: PRDM9 drives the location and rapid evolution of recombination hotspots in salmonid fish
Source: PLoS Biol. 2025 Jan 6;23(1):e3002950. doi: 10.1371/journal.pbio.3002950 (PMC11703093; doi:10.1371/journal.pbio.3002950)
Supplement: S7 Fig — (A) Distribution of DSB hotspots from TAC-1, TAC-3, and RT-52 along chromosomes (paces of 1/30 of chromosome length). (B) Average profile and heatmap of DMC1 ChIP-seq ssDNA fragments orientation (fragments per million, FPM) in TAC-1, TAC-3, and RT-52 testes, at DSB hotspots shared by pairs of samples. Shared DMC1 peaks: TAC-1 intersecting RT-52 (n = 167), TAC-1 intersecting TAC-3 (n = 55), and RT-52 intersecting TAC-3 (n = 42). The plots depict one replicate for each experiment performed (replicate 1). Signal mapped on the forward strand is depicted in blue, signal aligned to the reverse strand is shown in green. The data and codes underlying this figure can be found in https://doi.org/10.5281/zenodo.11083953 and https://zenodo.org/records/14198863. (DOCX) [file pbio.3002950.s022.docx]

**S7 Fig: Distribution of DSB hotspots along chromosomes in *O. mykiss*. A)** Distribution of DSB hotspots from TAC-1, TAC-3 and RT-52 along chromosomes (paces of 1/30 of chromosome length). **B)** Average profile and heatmap of DMC1 ChIP-seq ssDNA fragments orientation (fragments per million, FPM) in TAC-1, TAC-3 and RT-52 testes, at DSB hotspots shared by pairs of samples. Shared DMC1 peaks: TAC-1 intersecting RT-52 (n=167), TAC-1 intersecting TAC-3 (n=55), and RT-52 intersecting TAC-3 (n=42). The plots depict one replicate for each experiment performed (replicate 1). Signal mapped on the forward strand is depicted in blue, signal aligned to the reverse strand is shown in green. The data and codes underlying this figure can be found in <https://doi.org/10.5281/zenodo.11083953> and https://zenodo.org/records/14198863.
