# Supplementary material for: PRDM9 drives the location and rapid evolution of recombination hotspots in salmonid fish
Source: PLoS Biol. 2025 Jan 6;23(1):e3002950. doi: 10.1371/journal.pbio.3002950 (PMC11703093; doi:10.1371/journal.pbio.3002950)
Supplement: S8 Fig — (A) Average profile and heatmap of H3K36me3 ChIP-seq signal in TAC-1 (blue) and TAC-3 (green) testes, at H3K4me3 peaks detected in brain (Aqua-FAANG). (B) Average profile and heatmap of H3K4me3 (left) and H3K36me3 (right) ChIP-seq signal in TAC-1 testes, at DSB hotspots detected in TAC-1 (blue), TAC-3 (cyan), and RT-52 (yellow). (C) Average profile and heatmap of H3K4me3 (left) and H3K36me3 (right) ChIP-seq signal in TAC-3 testes, at DSB hotspots detected in TAC-1 (blue), TAC-3 (cyan), and RT-52 (yellow). The data underlying this figure can be found in https://doi.org/10.5281/zenodo.11083953. (DOCX) [file pbio.3002950.s023.docx]

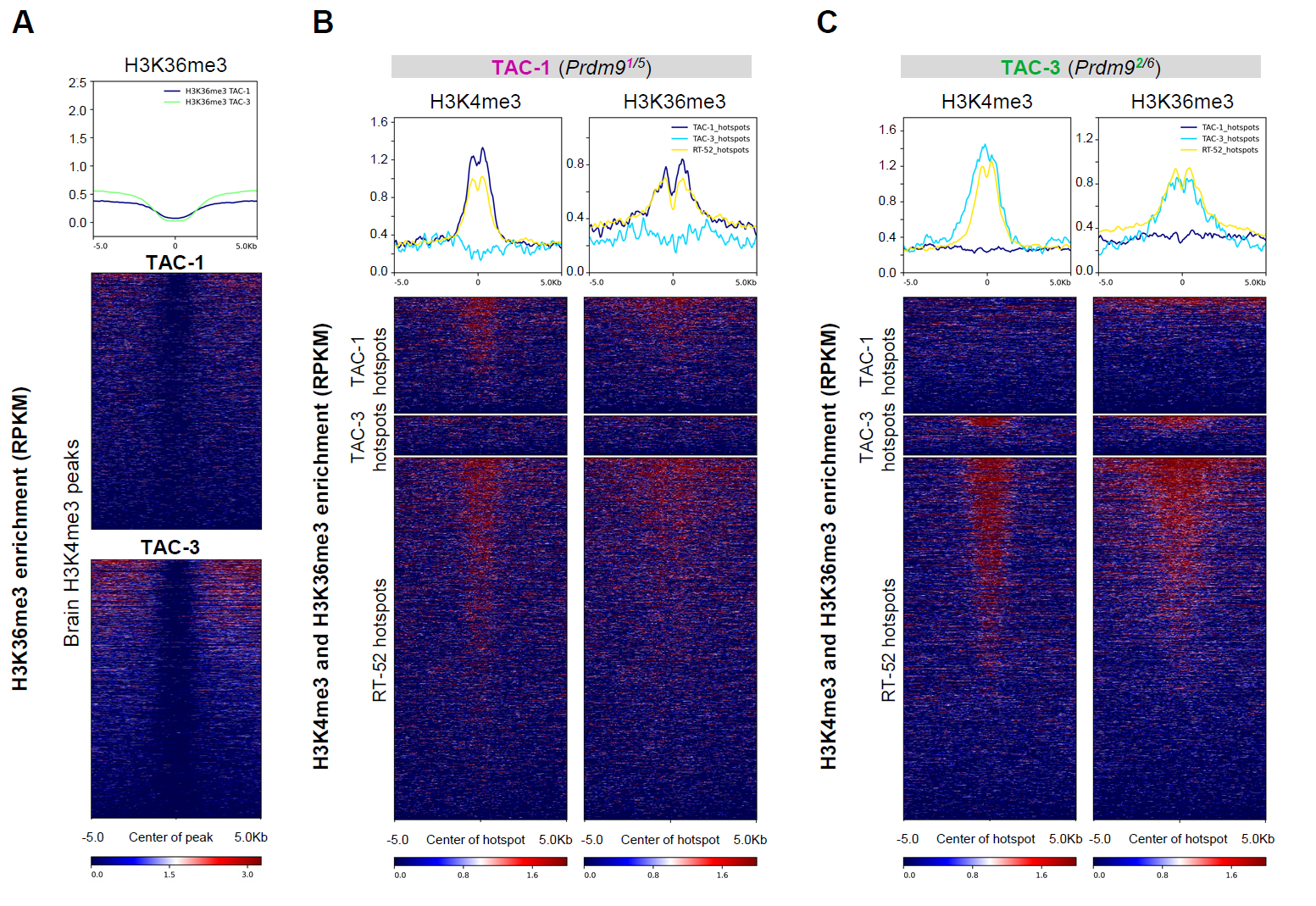


**S8 Fig: Histone modification signal at H3K4me3 peaks and at DSB hotspots. A)** Average profile and heatmap of H3K36me3 ChIP-seq signal in TAC-1 (blue) and TAC-3 (green) testes, at H3K4me3 peaks detected in brain (Aqua-FAANG). **B)** Average profile and heatmap of H3K4me3 (left) and H3K36me3 (right) ChIP-seq signal in TAC-1 testes, at DSB hotspots detected in TAC-1 (blue), TAC-3 (cyan) and RT-52 (yellow). **C)** Average profile and heatmap of H3K4me3 (left) and H3K36me3 (right) ChIP-seq signal in TAC-3 testes, at DSB hotspots detected in TAC-1 (blue), TAC-3 (cyan) and RT-52 (yellow). The data underlying this figure can be found in <https://doi.org/10.5281/zenodo.11083953>.
