# Supplementary material for: PRDM9 drives the location and rapid evolution of recombination hotspots in salmonid fish
Source: PLoS Biol. 2025 Jan 6;23(1):e3002950. doi: 10.1371/journal.pbio.3002950 (PMC11703093; doi:10.1371/journal.pbio.3002950)
Supplement: S9 Fig — (A) Scatterplots showing H3K4me3 and H3K36me3 ChIP-seq signal in TAC-1 and TAC-3 testes, at RT-52 DSB hotspots. (B) Left panels, scatterplots representing H3K4me3 (top) or H3K36me3 (bottom) ChIP-seq signal in TAC-1 and TAC-3 testes, at RT-52 DSB hotspots. Right panels, numbers of RT-52 hotspots with H3K4me3 (top) or H3K36me3 (bottom) ChIP-seq signal under or above 1 in TAC-1 and TAC-3. Chi-square test of homogeneity. The data underlying this figure can be found in https://doi.org/10.5281/zenodo.11083953. (DOCX) [file pbio.3002950.s024.docx]

**S9 Fig: Correlation of H3K4me3 and H3K36me3 signal at RT-52 hotspots. A)** Scatterplots showing H3K4me3 and H3K36me3 ChIP-seq signal in TAC-1 and TAC-3 testes, at RT-52 DSB hotspots. **B)** Left panels, scatterplots representing H3K4me3 (top) or H3K36me3 (bottom) ChIP-seq signal in TAC-1 and TAC-3 testes, at RT-52 DSB hotspots. Right panels, numbers of RT-52 hotspots with H3K4me3 (top) or H3K36me3 (bottom) ChIP-seq signal under or above 1 in TAC-1 and TAC-3. Chi-square test of homogeneity. The data underlying this figure can be found in https://doi.org/10.5281/zenodo.11083953.
