# Supplementary material for: PRDM9 drives the location and rapid evolution of recombination hotspots in salmonid fish
Source: PLoS Biol. 2025 Jan 6;23(1):e3002950. doi: 10.1371/journal.pbio.3002950 (PMC11703093; doi:10.1371/journal.pbio.3002950)
Supplement: S10 Fig — Recombination rates were averaged into percentiles of chromosome length and scaled by the genomic mean for (A) O. kisutch (in orange), O. mykiss (in green), and S. salar (in blue, only the NS population is shown); and (B) D. labrax (in red) and the three-spined stickleback Gasterosteus aculeatus (in black, data from [61]). The data and codes underlying this figure can be found in https://doi.org/10.5281/zenodo.11083953. (DOCX) [file pbio.3002950.s025.docx]

**
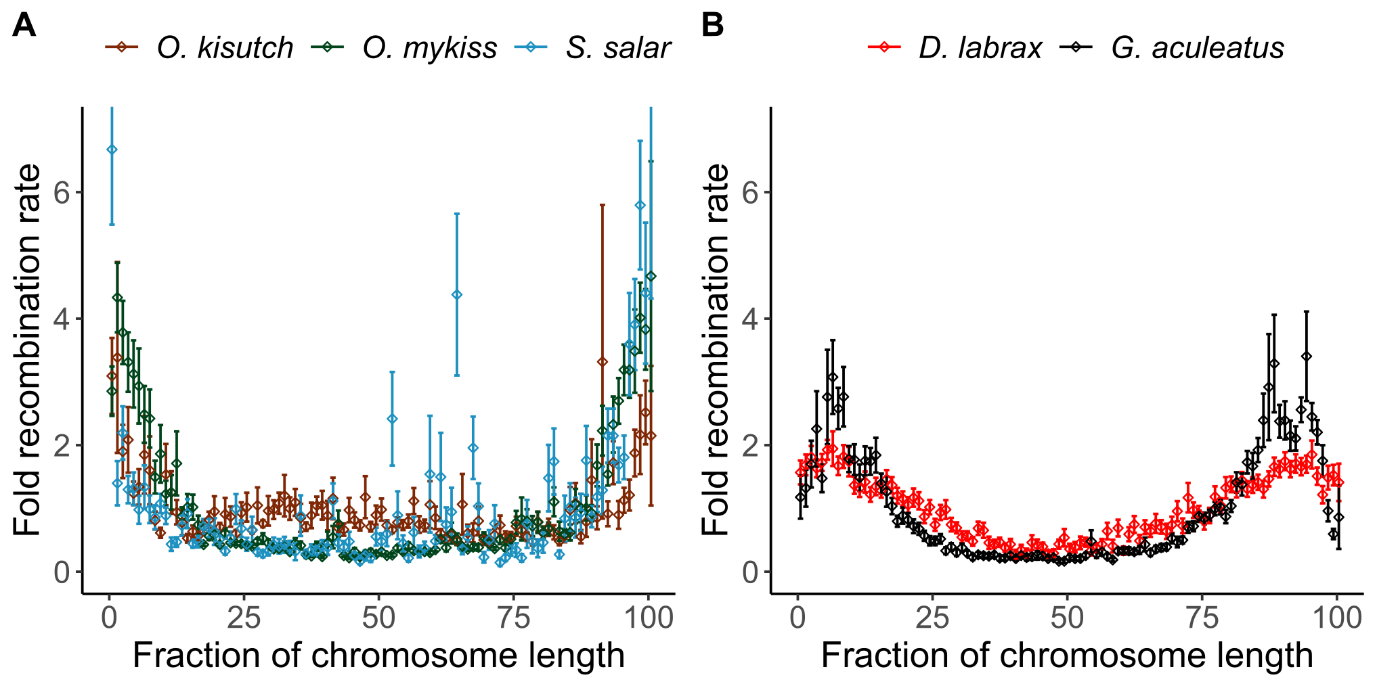
**

**S10 Fig:** **Broad scale recombination rate variations along the genome.** Recombination rates were averaged into percentiles of chromosome length, and scaled by the genomic mean for **A)** *O. kisutch* (in orange), *O. mykiss* (in green) and *S. salar* (in blue, only the NS population is shown); and **B)** *D. labrax* (in red) and the threespine stickleback *Gasterosteus aculeatus* (in black, data from (61)). The data and codes underlying this figure can be found in https://doi.org/10.5281/zenodo.11083953.
