# Supplementary material for: PRDM9 drives the location and rapid evolution of recombination hotspots in salmonid fish
Source: PLoS Biol. 2025 Jan 6;23(1):e3002950. doi: 10.1371/journal.pbio.3002950 (PMC11703093; doi:10.1371/journal.pbio.3002950)
Supplement: S11 Fig — The data and codes underlying this figure can be found in https://doi.org/10.5281/zenodo.11083953. (DOCX) [file pbio.3002950.s026.docx]

**
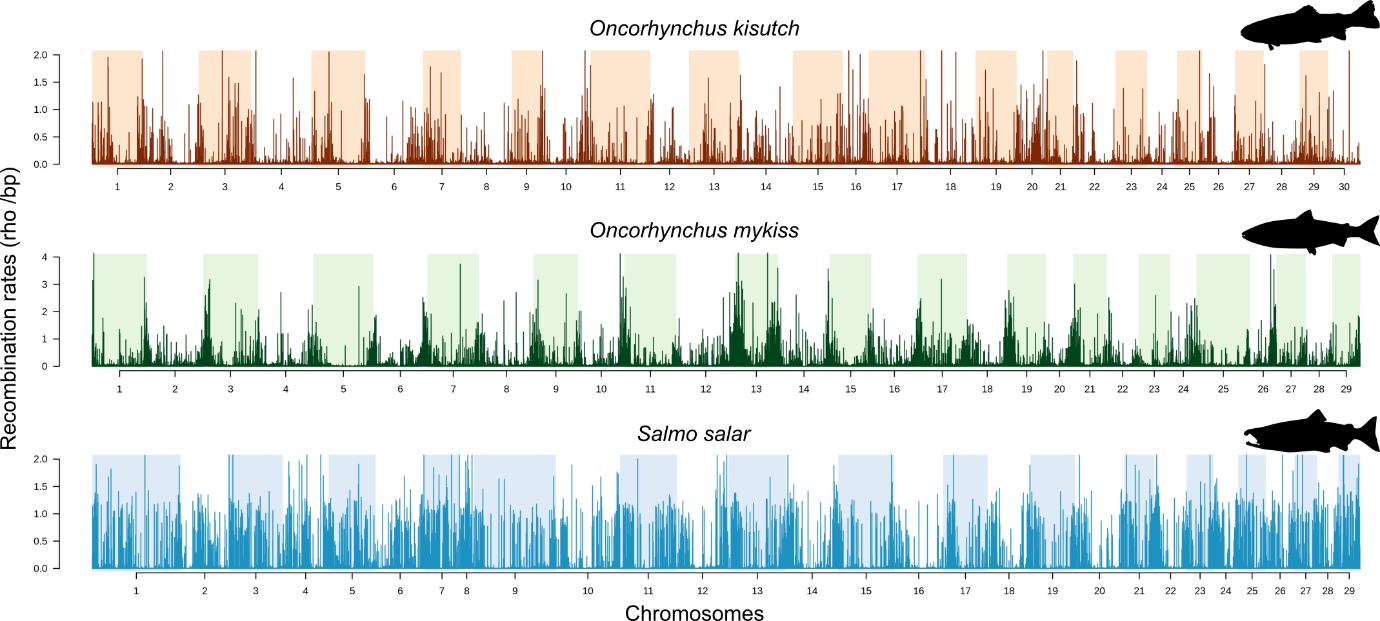
**

**S11 Fig:** Fine-scale recombination landscapes of *O. kisutch* (in orange), *O. mykiss* (in green) and *S. salar* (in blue, only the NS population is shown), with recombination rates smoothed in 2 kb sliding windows. The data and codes underlying this figure can be found in https://doi.org/10.5281/zenodo.11083953.
