# Supplementary material for: PRDM9 drives the location and rapid evolution of recombination hotspots in salmonid fish
Source: PLoS Biol. 2025 Jan 6;23(1):e3002950. doi: 10.1371/journal.pbio.3002950 (PMC11703093; doi:10.1371/journal.pbio.3002950)
Supplement: S12 Fig — The data and codes underlying this figure can be found in https://doi.org/10.5281/zenodo.11083953. (DOCX) [file pbio.3002950.s027.docx]

**
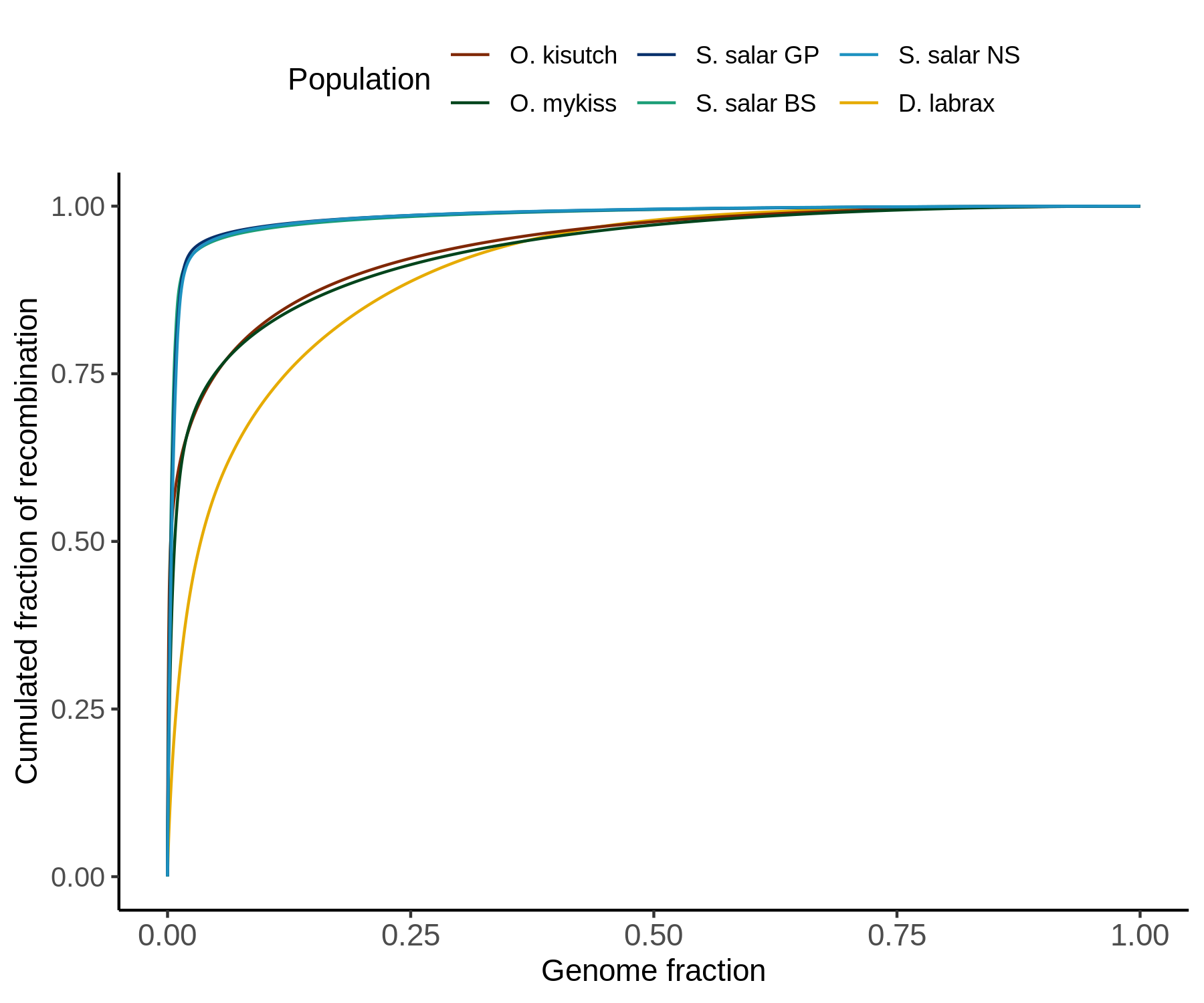
**

**S12 Fig:** Proportion of recombination according to proportion of the genome for *O. kisutch* (orange), *O. mykiss* (green), *S. salar* (shades of blue) and *D. labrax* (gold). The data and codes underlying this figure can be found in https://doi.org/10.5281/zenodo.11083953.
