# Supplementary material for: PRDM9 drives the location and rapid evolution of recombination hotspots in salmonid fish
Source: PLoS Biol. 2025 Jan 6;23(1):e3002950. doi: 10.1371/journal.pbio.3002950 (PMC11703093; doi:10.1371/journal.pbio.3002950)
Supplement: S13 Fig — Hotspots were defined as consecutives windows of 2 adjacent SNPs in which the recombination rate is at least 5-fold higher than the 50 kb flanking regions. The data and codes underlying this figure can be found in https://doi.org/10.5281/zenodo.11083953. (DOCX) [file pbio.3002950.s028.docx]

**
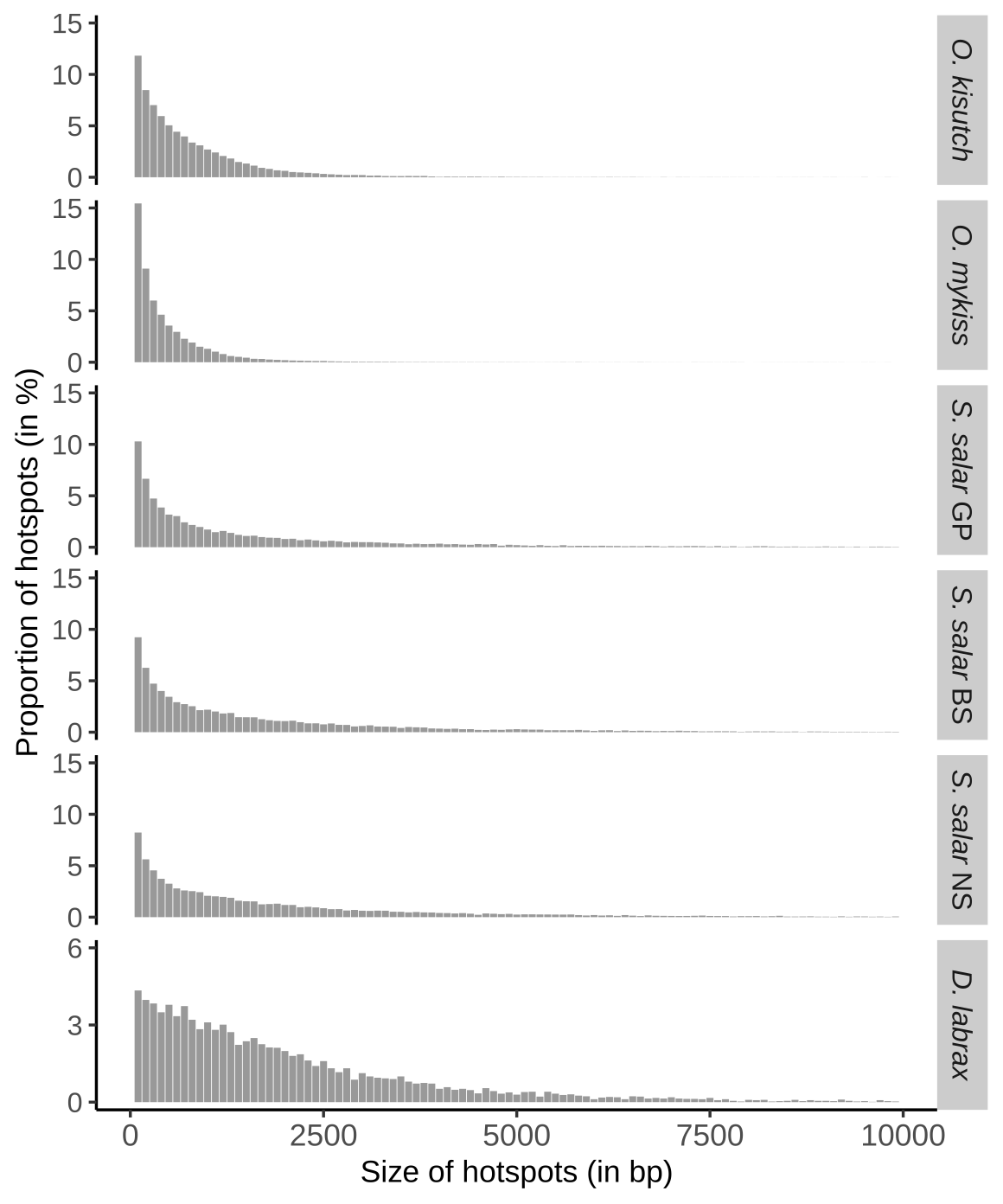
**

**S13 Fig:** Proportion of hotspots (in %) according to raw hotspot size. Hotspots were defined as consecutives windows of two adjacent SNPs in which the recombination rate is at least 5-fold higher than the 50 kb flanking regions. The data and codes underlying this figure can be found in https://doi.org/10.5281/zenodo.11083953.
