# Supplementary material for: PRDM9 drives the location and rapid evolution of recombination hotspots in salmonid fish
Source: PLoS Biol. 2025 Jan 6;23(1):e3002950. doi: 10.1371/journal.pbio.3002950 (PMC11703093; doi:10.1371/journal.pbio.3002950)
Supplement: S14 Fig — (A) Venn diagram showing the percentage of shared peaks between the ChIP-Seq peaks of the pooled samples (in brown) and the LD-based hotspots (in green). The percentage has been calculated using the number of DMC1 peaks as the denominator. (B) Random expected (blue) and observed values (orange) of shared peaks between LD and ChIP-Seq maps. (C) Recombination rates ⍴ in the syntenic location of the ChIP-Seq peaks, in the LD hotspots, in the shared ChIP-Seq and LD windows (i.e., 116 ChIP-Seq peaks shared with LD hotspots) and in the background landscapes (i.e., the genomic windows not containing neither a LD hotspot nor a ChIP-Seq peak). The data and codes underlying this figure can be found in https://doi.org/10.5281/zenodo.11083953. (DOCX) [file pbio.3002950.s029.docx]

**
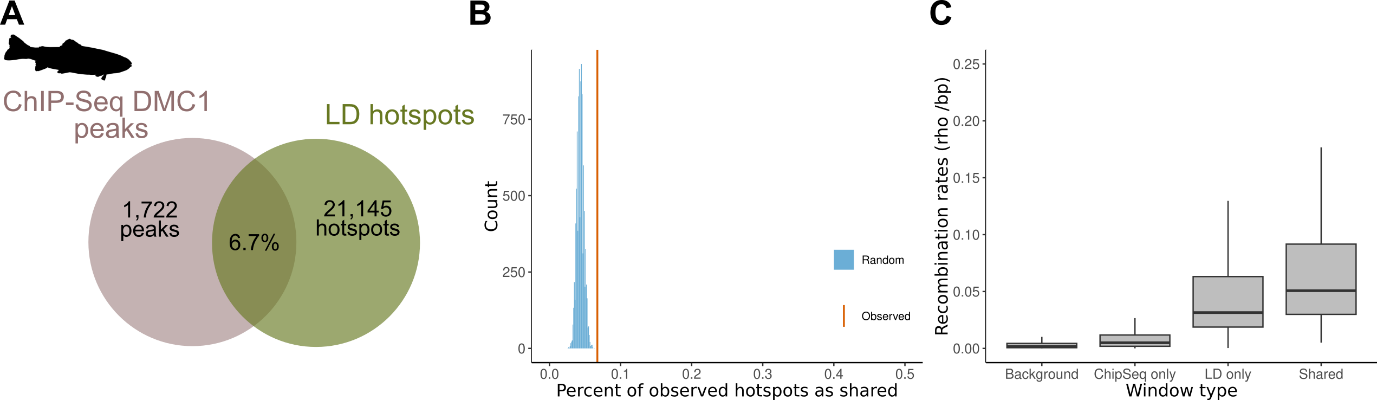
**

**S14 Fig: Comparison between the LD-based recombination landscape and the ChIP-Seq DMC1 map of the rainbow trout *O. mykiss*.** **A)** Venn diagram showing the percentage of shared peaks between the ChIP-Seq peaks of the pooled samples (in brown) and the LD-based hotspots (in green). The percentage has been calculated using the number of DMC1 peaks as the denominator. **B)** Random expected (blue) and observed values (orange) of shared peaks between LD and ChIP-Seq maps. **C)** Recombination rates *⍴* in the syntenic location of the ChIP-Seq peaks, in the LD hotspots, in the shared ChIP-Seq and LD windows (*i.e.* 116 ChIP-Seq peaks shared with LD hotspots) and in the background landscapes (*i.e.* the genomic windows not containing neither a LD hotspot nor a ChIP-Seq peak). The data and codes underlying this figure can be found in https://doi.org/10.5281/zenodo.11083953.
