# Supplementary material for: PRDM9 drives the location and rapid evolution of recombination hotspots in salmonid fish
Source: PLoS Biol. 2025 Jan 6;23(1):e3002950. doi: 10.1371/journal.pbio.3002950 (PMC11703093; doi:10.1371/journal.pbio.3002950)
Supplement: S15 Fig — (A) SNP density (per kb). (B) GC-content. (C) TE density (per 100 kb). Recombination rates, SNP density GC-content, and TE density were averaged in 100 kb sliding windows. Significance p-value of Spearman’s rank test <0.05 are indicated by an asterisk in panels. The vertical dashed line is the mean recombination rates and the horizontal dashed line is the mean y variable. The data and codes underlying this figure can be found in https://doi.org/10.5281/zenodo.11083953. (DOCX) [file pbio.3002950.s030.docx]

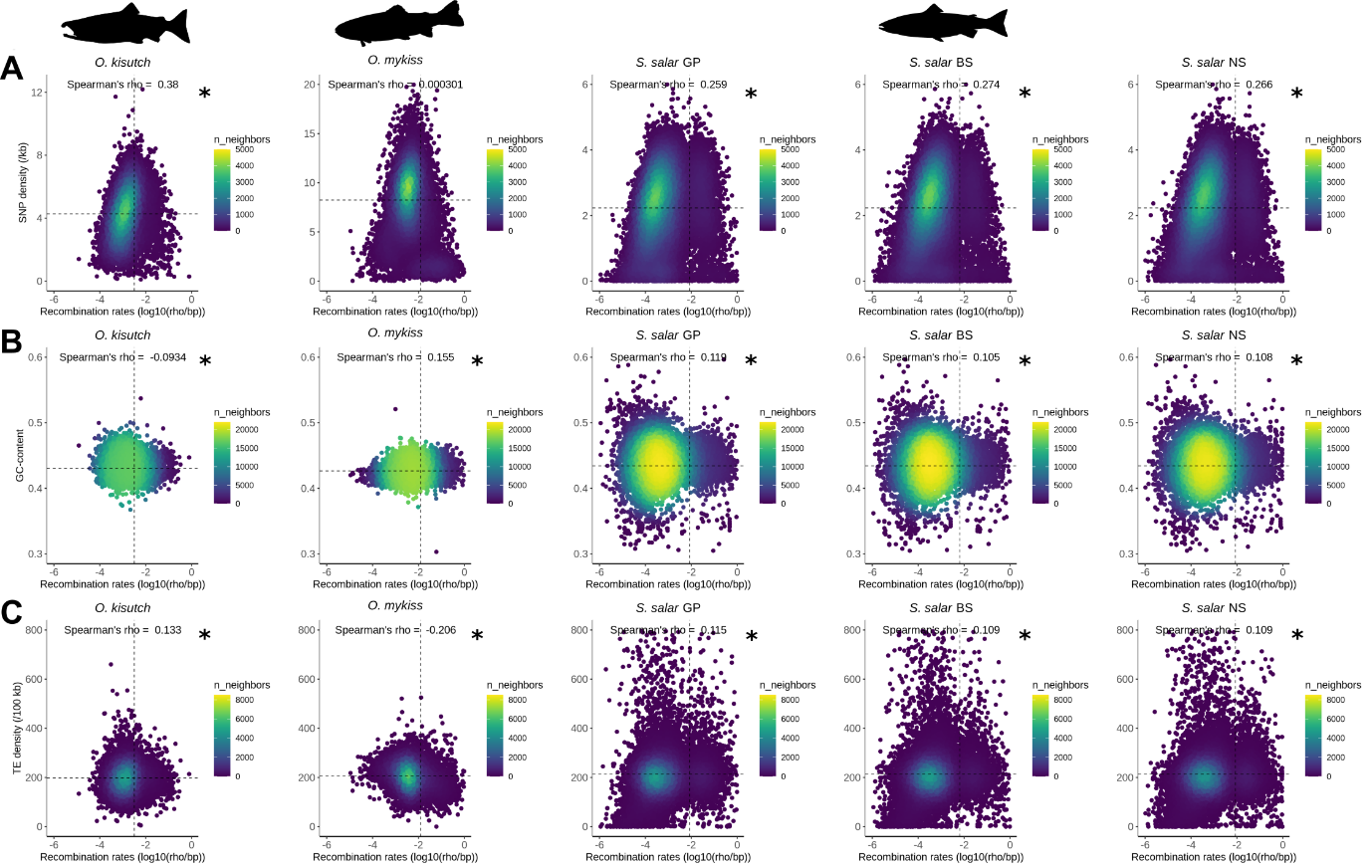


**S15 Fig: Broad scale variation in genomic variables according to recombination rates. A)** SNP density (per kb). **B)** GC-content. **C)** TE density (per 100 kb). Recombination rates, SNP density GC-content and TE density were averaged in 100 kb sliding windows. Significance p-value of Spearman’s rank test < 0.05 are indicated by an asterisk in panels. The vertical dashed line is the mean recombination rates and the horizontal dashed line is the mean y variable. The data and codes underlying this figure can be found in https://doi.org/10.5281/zenodo.11083953.
