# Supplementary material for: PRDM9 drives the location and rapid evolution of recombination hotspots in salmonid fish
Source: PLoS Biol. 2025 Jan 6;23(1):e3002950. doi: 10.1371/journal.pbio.3002950 (PMC11703093; doi:10.1371/journal.pbio.3002950)
Supplement: S16 Fig — (A) SNPs density (per kb) and (B) GC content, according to distance to the nearest recombination hotspots. SNP density, GC-content, and recombination rates were averaged in 2 kb windows. Colored (orange, green, and blue) dashed lines show the mean of the y variable at hotspots of the corresponding populations, the black dashed line is the genomic mean (outside hotspots). Loess curves are shown for a span of 0.5. The data and codes underlying this figure can be found in https://doi.org/10.5281/zenodo.11083953. (DOCX) [file pbio.3002950.s031.docx]

**
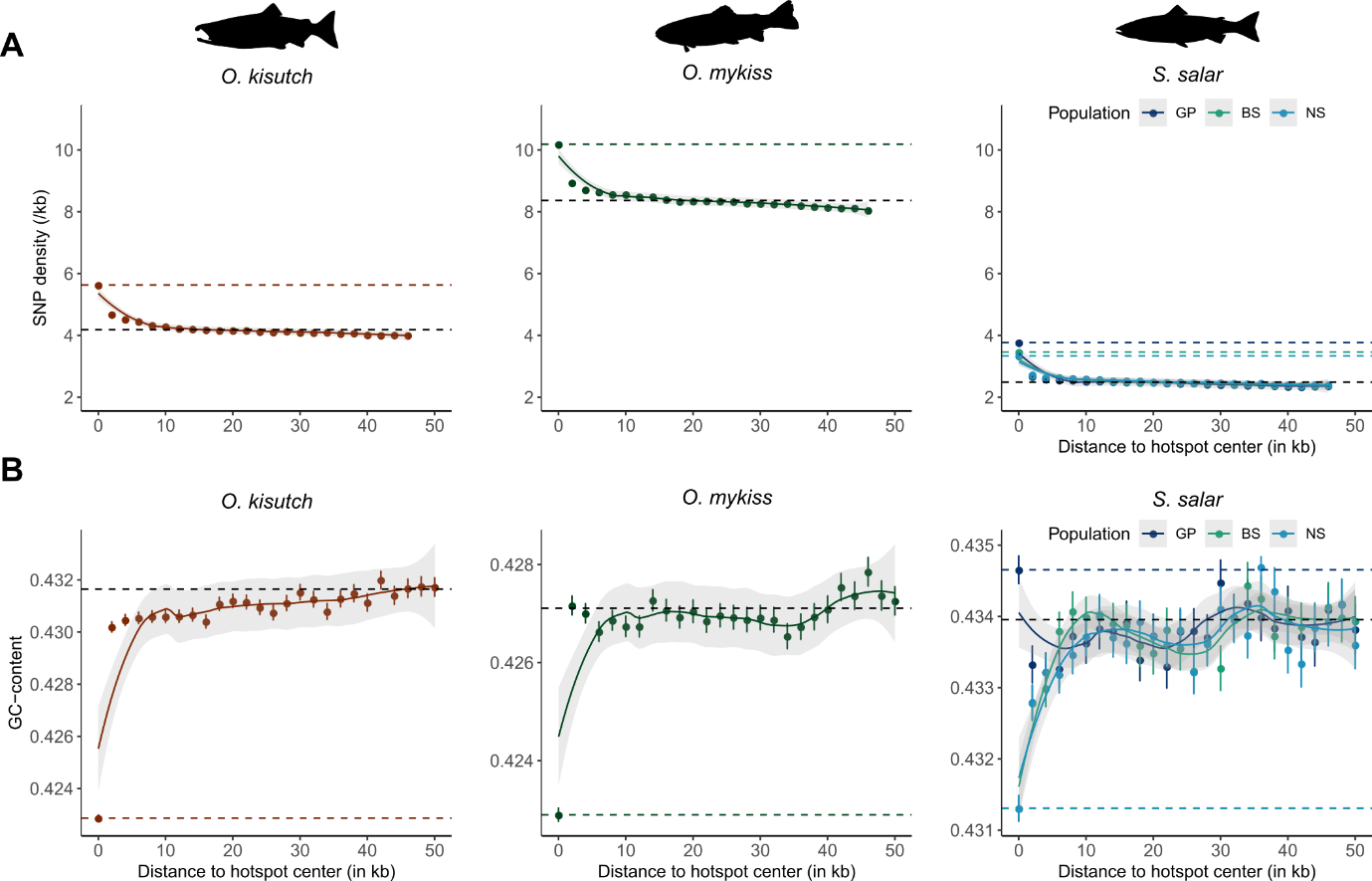
**

**S16 Fig: Genetic diversity and base composition at recombination hotspots. A)** SNPs density (per kb), and **B)** GC content, according to distance to the nearest recombination hotspots. SNP density, GC-content and recombination rates were averaged in 2 kb windows. Colored (orange, green blue) dashed lines show the mean of the y variable at hotspots of the corresponding populations, the black dashed line is the genomic mean (outside hotspots). Loess curves are shown for a span of 0.5. The data and codes underlying this figure can be found in https://doi.org/10.5281/zenodo.11083953.
