# Supplementary material for: PRDM9 drives the location and rapid evolution of recombination hotspots in salmonid fish
Source: PLoS Biol. 2025 Jan 6;23(1):e3002950. doi: 10.1371/journal.pbio.3002950 (PMC11703093; doi:10.1371/journal.pbio.3002950)
Supplement: S18 Fig — (A) Averaged recombination rate per chromosome. Gray bars indicate chromosomes without residual tetrasomic regions (2N), and yellow bars indicate chromosomes with residual tetrasomic regions (4N) in the 5 Oncorhynchus and Salmo populations. Gray dashed lines represent the genome averaged recombination rates of the 2N chromosomes, and the yellow line in the 4N chromosomes. Recombination rates are significantly higher in 4N chromosomes compared to 2N chromosomes (Student test, t(13.258) = −3.9404, p < 0.05) in O. mykiss population, but not in O. kisutch (Student test, t(25.867) = −0.88786, p > 0.05) neither in S. salar populations (Student test, t(23.519) = −0.0026857, p > 0.05 for GP, t(20.99) = −2.2572, p < 0.05 for BS and t(19.677) = −1.9741, p = 0.06258 for NS). (B) Recombination rates along the genome. Recombination rates were averaged into percentiles of chromosome length and scaled by the genomic mean. Same color as panel A. The data and codes underlying this figure can be found in https://doi.org/10.5281/zenodo.11083953. (DOCX) [file pbio.3002950.s033.docx]

**
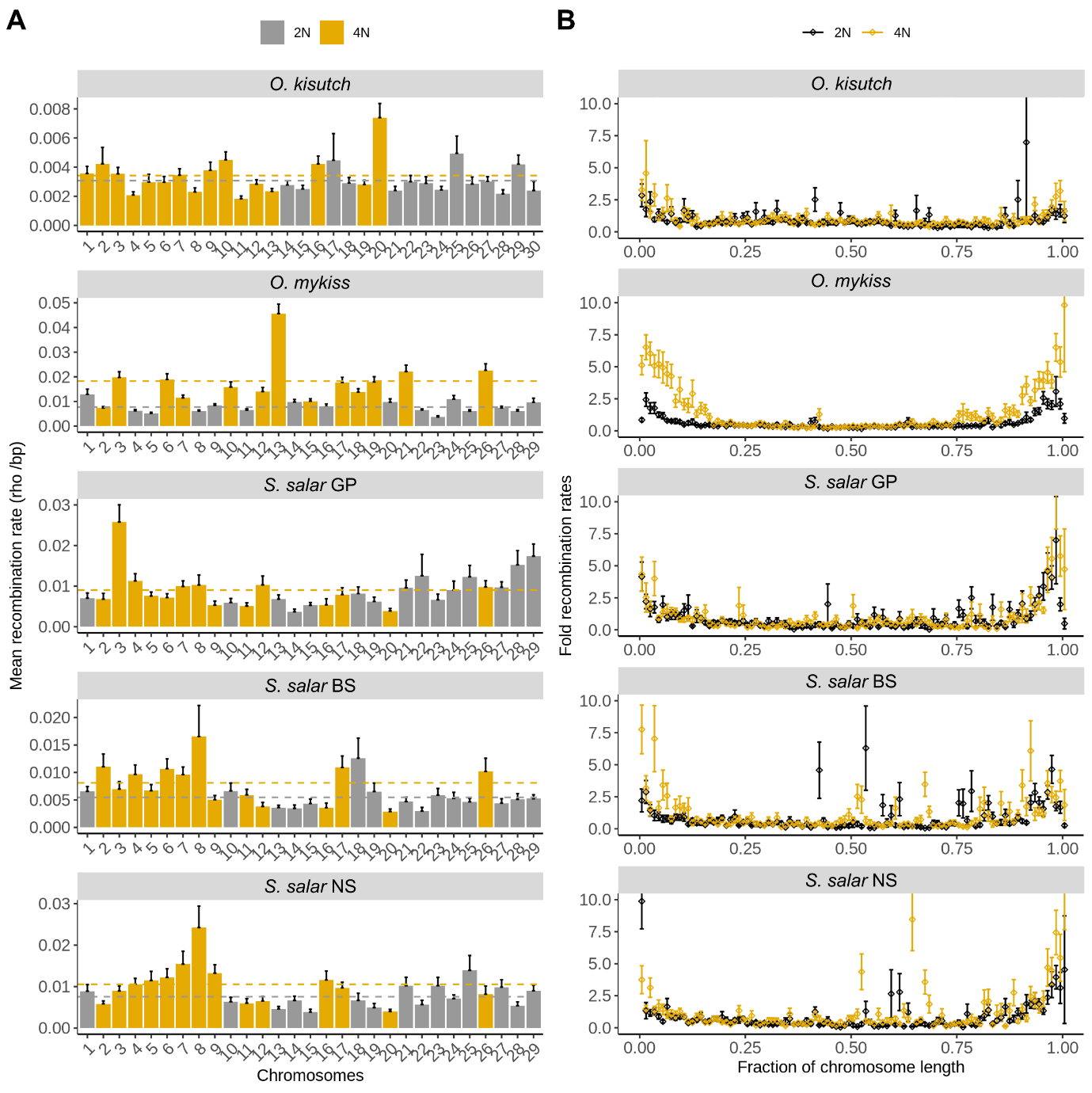
**

**S18 Fig: Inter- and intra-chromosome variation in recombination rates in residual tetraploid chromosomes. A)** Averaged recombination rate per chromosome. Grey bars indicate chromosomes without residual tetrasomic regions (2N), and yellow bars indicate chromosomes with residual tetrasomic regions (4N) in the five *Oncorhynchus* and *Salmo* populations. Grey dashed lines represent the genome averaged recombination rates of the 2N chromosomes, and the yellow line in the 4N chromosomes. Recombination rates are significantly higher in 4N chromosomes compared to 2N chromosomes (Student test, t(13.258) =-3.9404, p < 0.05) in *O. mykiss* population, but not in *O. kisutch* (Student test, t(25.867) =-0.88786, p > 0.05) neither in *S. salar* populations (Student test, t(23.519) =-0.0026857, p > 0.05 for GP, t(20.99) = -2.2572, p < 0.05 for BS and t(19.677) =-1.9741, p = 0.06258 for NS). **B)** Recombination rates along the genome. Recombination rates were averaged into percentiles of chromosome length, and scaled by the genomic mean. Same color as panel A. The data and codes underlying this figure can be found in https://doi.org/10.5281/zenodo.11083953.
