# Supplementary material for: PRDM9 drives the location and rapid evolution of recombination hotspots in salmonid fish
Source: PLoS Biol. 2025 Jan 6;23(1):e3002950. doi: 10.1371/journal.pbio.3002950 (PMC11703093; doi:10.1371/journal.pbio.3002950)
Supplement: S19 Fig — (A) Fold recombination rates (scaled by the average recombination rate at 50 kb from the nearest feature) according to distance to the nearest promoter-like features (i.e., TSS overlapping or not a CGI). Recombination rates in chromosomes not containing residual tetraploid regions are shown by the continuous line and by the dashed line for the 4N chromosomes. (B) Fold recombination rates (scaled by the average recombination rates in intergenic regions) in genomic features, in 2N (gray) and 4N (yellow) chromosomes. The horizontal line shows the intergenic recombination level. TSS and TES were defined as the first and last positions of genes. CGIs were mapped with EMBOSS using CpGoe > 0.6 and GC > 0. The data and codes underlying this figure can be found in https://doi.org/10.5281/zenodo.11083953. (DOCX) [file pbio.3002950.s034.docx]

**
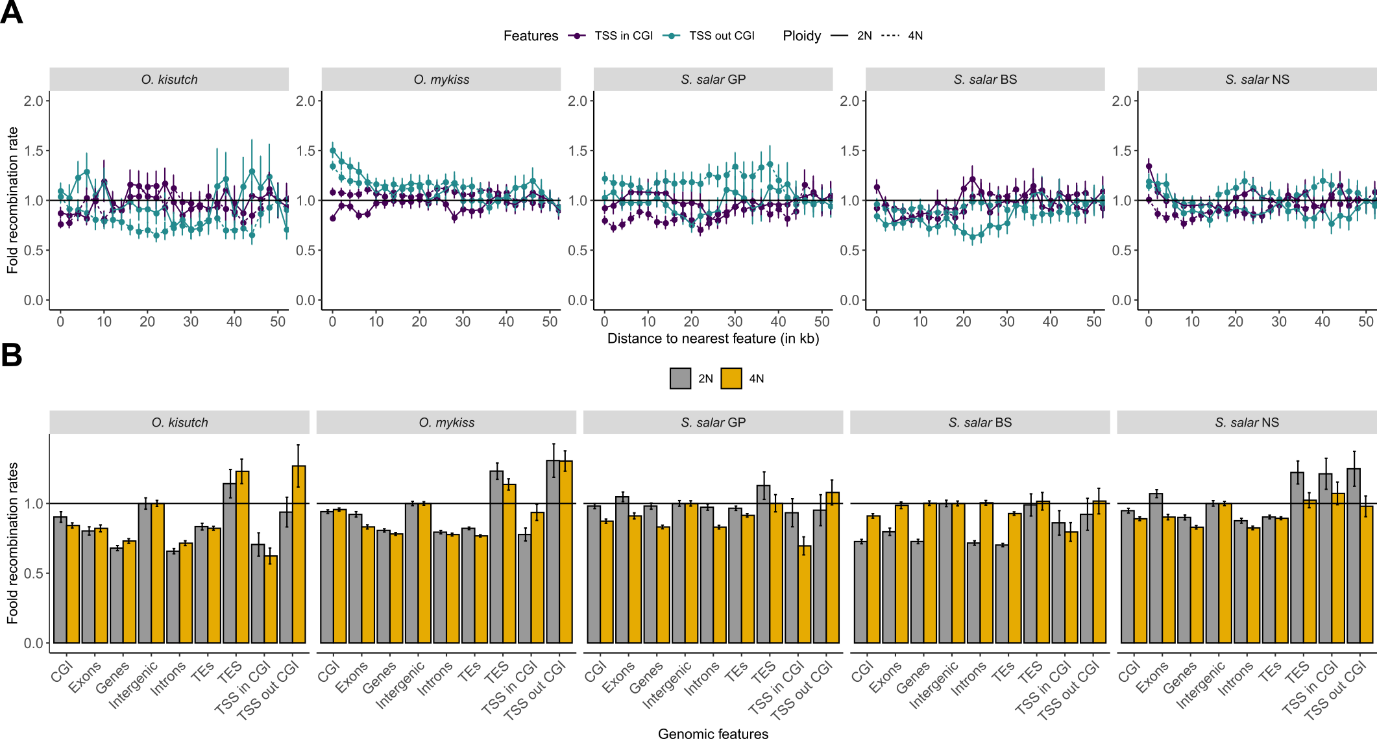
**

**S19 Fig: Recombination rates at genomic features in residual tetraploid chromosomes. A)** Fold recombination rates (scaled by the average recombination rate at 50 kb from the nearest feature) according to distance to the nearest promoter-like features (*i.e.* TSS overlapping or not a CGI). Recombination rates in chromosomes not containing residual tetraploid regions are shown by the continuous line, and by the dashed line for the 4N chromosomes. **B)** Fold recombination rates (scaled by the average recombination rates in intergenic regions) in genomic features, in 2N (grey) and 4N (yellow) chromosomes. The horizontal line shows the intergenic recombination level. TSS and TES were defined as the first and last positions of genes. CGIs were mapped with EMBOSS using CpGoe > 0.6 and GC > 0. The data and codes underlying this figure can be found in https://doi.org/10.5281/zenodo.11083953.
