# Supplementary material for: PRDM9 drives the location and rapid evolution of recombination hotspots in salmonid fish
Source: PLoS Biol. 2025 Jan 6;23(1):e3002950. doi: 10.1371/journal.pbio.3002950 (PMC11703093; doi:10.1371/journal.pbio.3002950)
Supplement: S20 Fig — Random expectations (blue) and observed values (orange) of shared hotspots between (A) O. kisutch and O. mykiss; between S. salar populations (B) GP and BS; (C) GP and NS; and between (D) BS and NS. Shared hotspots were defined as 2 kb hotspots overlapping by at least 1 bp. Percent shared is calculated using the number of hotspots in the species/population with fewer hotspots as the denominator. The expected distribution of shared hotspots has been obtained from 1,000 pairwise comparisons of random spot. The data and codes underlying this figure can be found in https://doi.org/10.5281/zenodo.11083953. (DOCX) [file pbio.3002950.s035.docx]

**
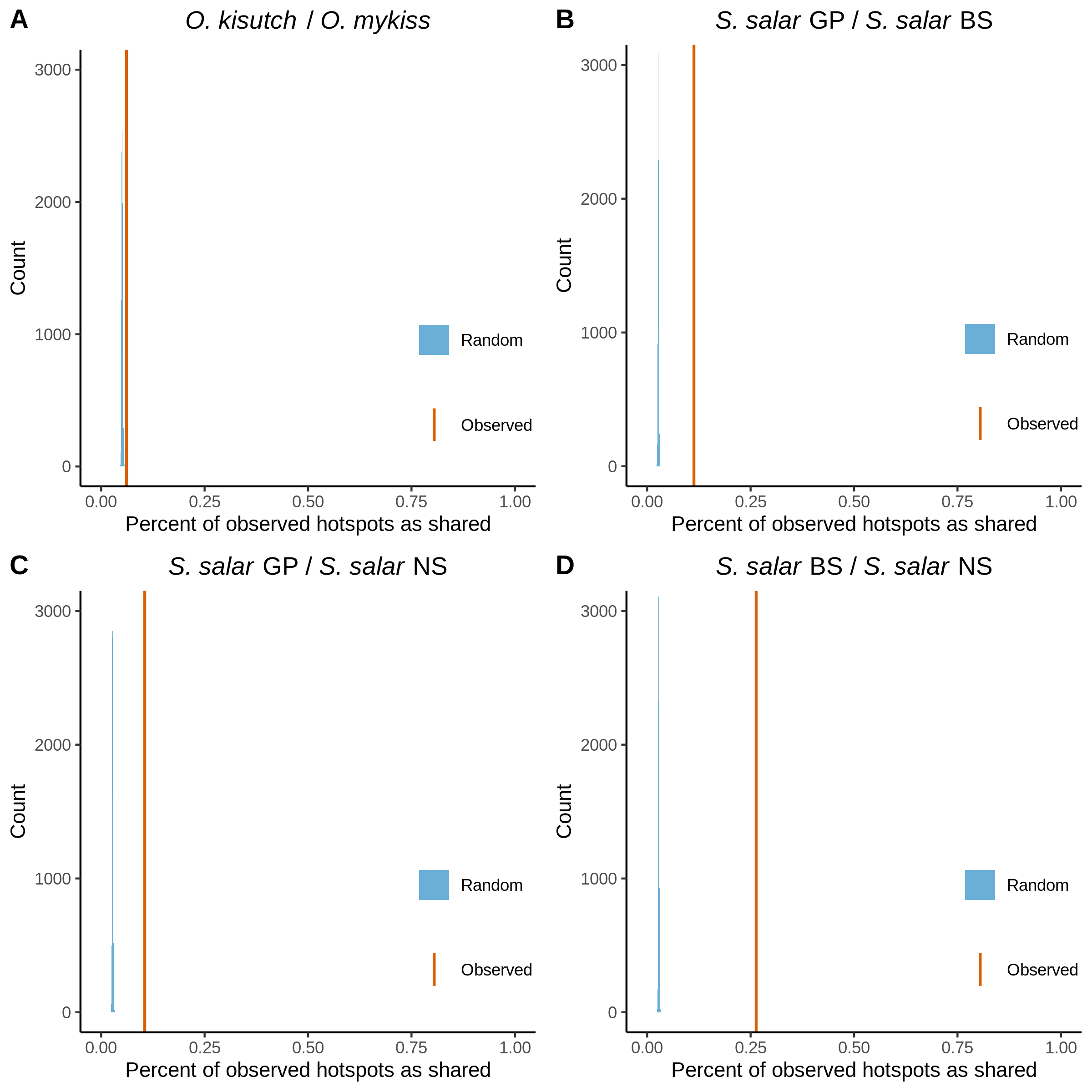
**

**S20 Fig: Significance of hotspots sharing between closely related populations.** Random expectations (blue) and observed values (orange) of shared hotspots between **A)** *O. kisutch* and *O. mykiss*; between *S. salar* populations **B)** GP and BS; **C)** GP and NS; and between **D)** BS and NS. Shared hotspots were defined as 2 kb hotspots overlapping by at least 1 bp. Percent shared is calculated using the number of hotspots in the species/population with fewer hotspots as the denominator. The expected distribution of shared hotspots has been obtained from 1000 pairwise comparisons of random spot. The data and codes underlying this figure can be found in https://doi.org/10.5281/zenodo.11083953.
