# Supplementary material for: PRDM9 drives the location and rapid evolution of recombination hotspots in salmonid fish
Source: PLoS Biol. 2025 Jan 6;23(1):e3002950. doi: 10.1371/journal.pbio.3002950 (PMC11703093; doi:10.1371/journal.pbio.3002950)
Supplement: S21 Fig — (A) Comparison between GP and BS populations. (B) Comparison between GP and NS populations. (C) Comparison between BS and NS populations. Spearman’s rank test p-value <0.05. Loess curves are shown for a span of 0.7. The data and codes underlying this figure can be found in https://doi.org/10.5281/zenodo.11083953. (DOCX) [file pbio.3002950.s036.docx]

**
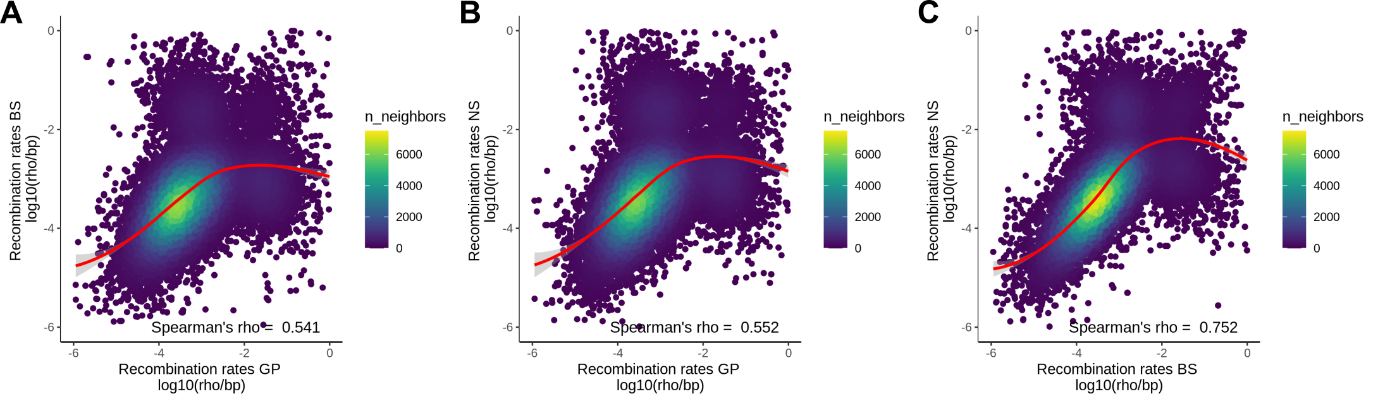
**

**S21 Fig: Pairwise comparison of 100 kb smoothed recombination maps between *S. salar* populations. A)** Comparison between GP and BS populations. **B)** Comparison between GP and NS populations. **C)** Comparison between BS and NS populations. Spearman’s rank test p-value < 0.05. Loess curves are shown for a span of 0.7. The data and codes underlying this figure can be found in https://doi.org/10.5281/zenodo.11083953.
