# Supplementary material for: PRDM9 drives the location and rapid evolution of recombination hotspots in salmonid fish
Source: PLoS Biol. 2025 Jan 6;23(1):e3002950. doi: 10.1371/journal.pbio.3002950 (PMC11703093; doi:10.1371/journal.pbio.3002950)
Supplement: S22 Fig — Frequency of sequences with at least one hit for PRDM9 allele 1 (left) and allele 2 (right) motifs at allele 1 and allele 2 sites, RT-52, TAC-1 and TAC-3 DSB hotspots, LD-hotspots and control sites. Fold enrichment relative to the control sites is shown on top of each column. The associated p-values indicate significant differences in fold enrichment relative to the control (Fisher exact test). “NS” indicates not significant (p > 0.05). The data and codes underlying this figure can be found in https://doi.org/10.5281/zenodo.11083953. (DOCX) [file pbio.3002950.s037.docx]

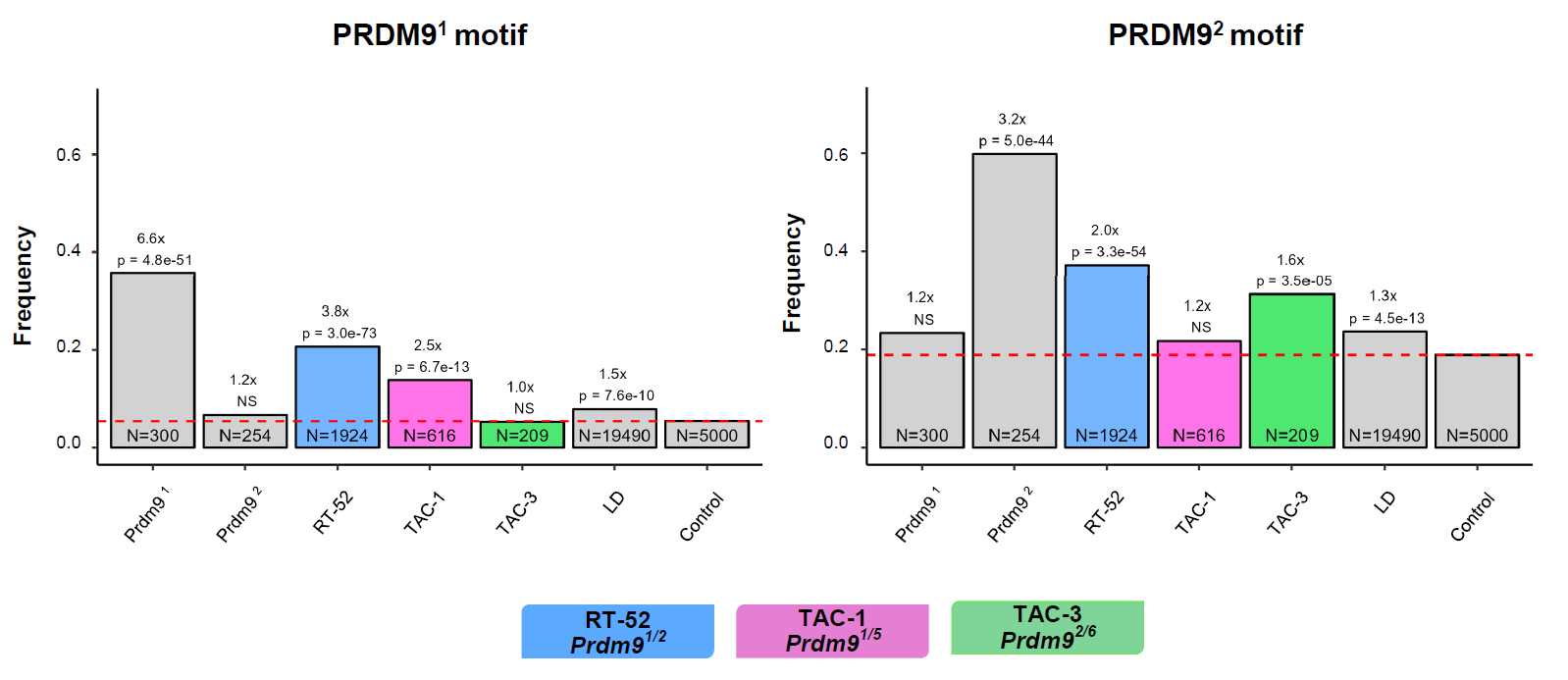


**S22 Fig: DSB and LD hotspots are enriched in PRDM9 allele-specific motifs.** Frequency of sequences with at least one hit for PRDM9 allele 1 (left) and allele 2 (right) motifs at allele 1 and allele 2 sites, RT-52, TAC-1 and TAC-3 DSB hotspots, LD-hotspots and control sites. Fold enrichment relative to the control sites is shown on top of each column. The associated p-values indicate significant differences in fold enrichment relative to the control (Fisher exact test). “NS” indicates not significant (p>0.05). The data and codes underlying this figure can be found in https://doi.org/10.5281/zenodo.11083953.
