# Supplementary material for: PRDM9 drives the location and rapid evolution of recombination hotspots in salmonid fish
Source: PLoS Biol. 2025 Jan 6;23(1):e3002950. doi: 10.1371/journal.pbio.3002950 (PMC11703093; doi:10.1371/journal.pbio.3002950)
Supplement: S23 Fig — Positional distribution of hits for Prdm9 allele 1 (pink) and allele 2 motifs (green) in RT-52, TAC-1, TAC-3 DSB hotspots, LD stronger hotspots (n = 5,000) and control sites (n = 5,000). The distribution is shown from the center of the sequence with a range of ±2.5 kb for the DSB hotspots and the control sites. The LD hotspots were centered on the SNP interval showing the highest recombination rate (ρ/bp) and the distribution extends up to 7.5 kb from the refined center. The signal is smoothed by weighted moving average and hits were calculated either in a 750 bp window for the LD hotpots and in a 250 bp window for all other sequences. The statistical significance of motif enrichment, adjusted for multiple tests, is shown (one-tail binomial test). “ns” indicates non-significant enrichment (p > 0.05). The data underlying this figure can be found in https://doi.org/10.5281/zenodo.11083953. (DOCX) [file pbio.3002950.s038.docx]

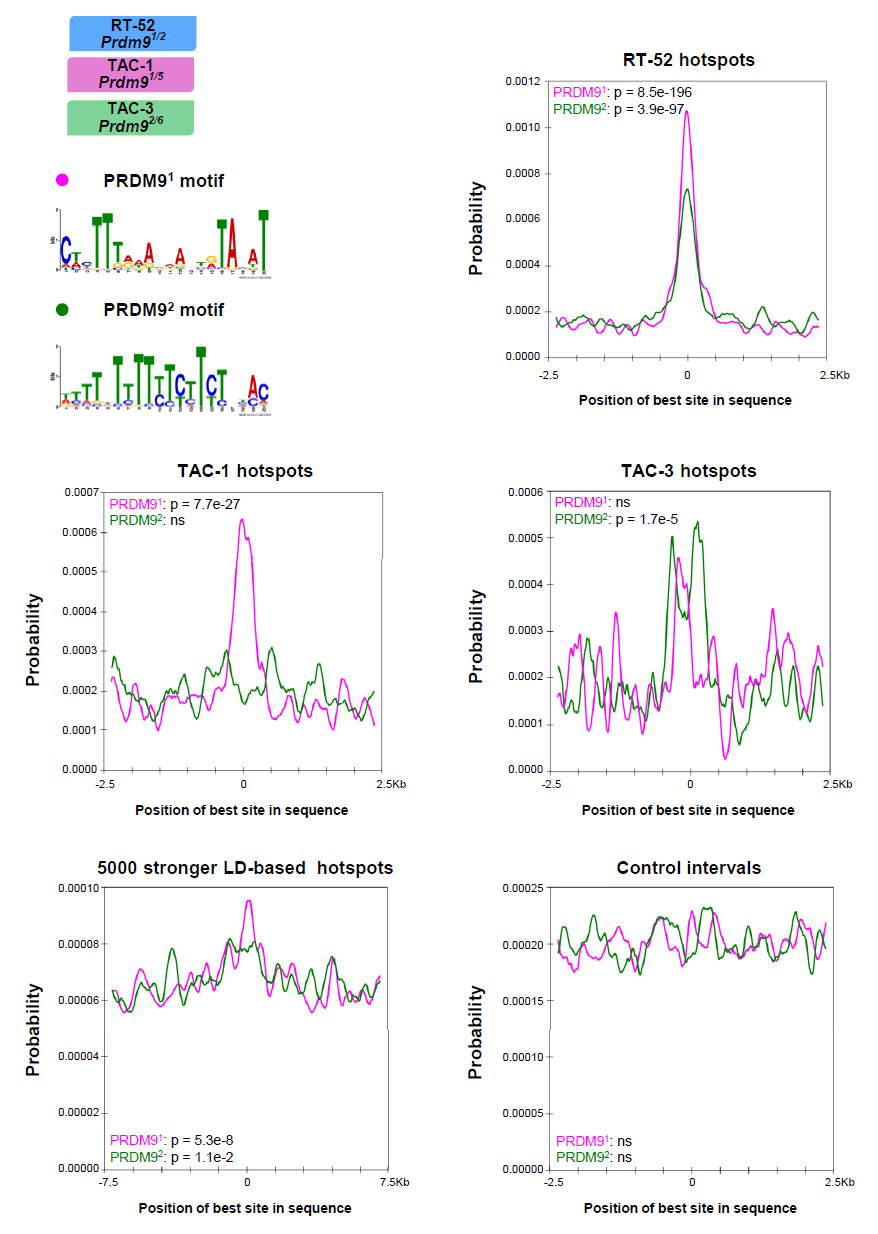


**S23 Fig: DSB and LD-based hotspots are enriched in PRDM9 allele-specific motifs.** Positional distribution of hits for *Prdm9* allele 1 (pink) and allele 2 motifs (green) in RT-52, TAC-1, TAC-3 DSB hotspots, LD stronger hotspots (n=5000) and control sites (n=5000). The distribution is shown from the center of the sequence with a range of ± 2.5 kb for the DSB hotspots and the control sites. The LD hotspots were centered on the SNP interval showing the highest recombination rate (ρ/bp) and the distribution extends up to 7.5 kb from the refined center. The signal is smoothed by weighted moving average and hits were calculated either in a 750 bp window for the LD hotpots and in a 250 bp window for all other sequences. The statistical significance of motif enrichment, adjusted for multiple tests, is shown (one-tail binomial test). “ns” indicates non-significant enrichment (p>0.05). The data underlying this figure can be found in https://doi.org/10.5281/zenodo.11083953.
