# Supplementary material for: PRDM9 drives the location and rapid evolution of recombination hotspots in salmonid fish
Source: PLoS Biol. 2025 Jan 6;23(1):e3002950. doi: 10.1371/journal.pbio.3002950 (PMC11703093; doi:10.1371/journal.pbio.3002950)
Supplement: S24 Fig — (A) Average recombination rate in motifs found enriched at hotspot. Yellow boxes show motifs found in at least 5% of hotspots showing 2-fold enrichment compared to the control set of random spots. (B) Average recombination rate in hotspots containing the retained motifs from panel A (with the corresponding motifs shown) compared to hotspots not containing the retained motifs. Significant Student’s tests are indicated (***, p-value <0.05). (C) Percentage of hotspots containing the retained motifs shown in yellow. The data and codes underlying this figure can be found in https://doi.org/10.5281/zenodo.11083953. (DOCX) [file pbio.3002950.s039.docx]

**
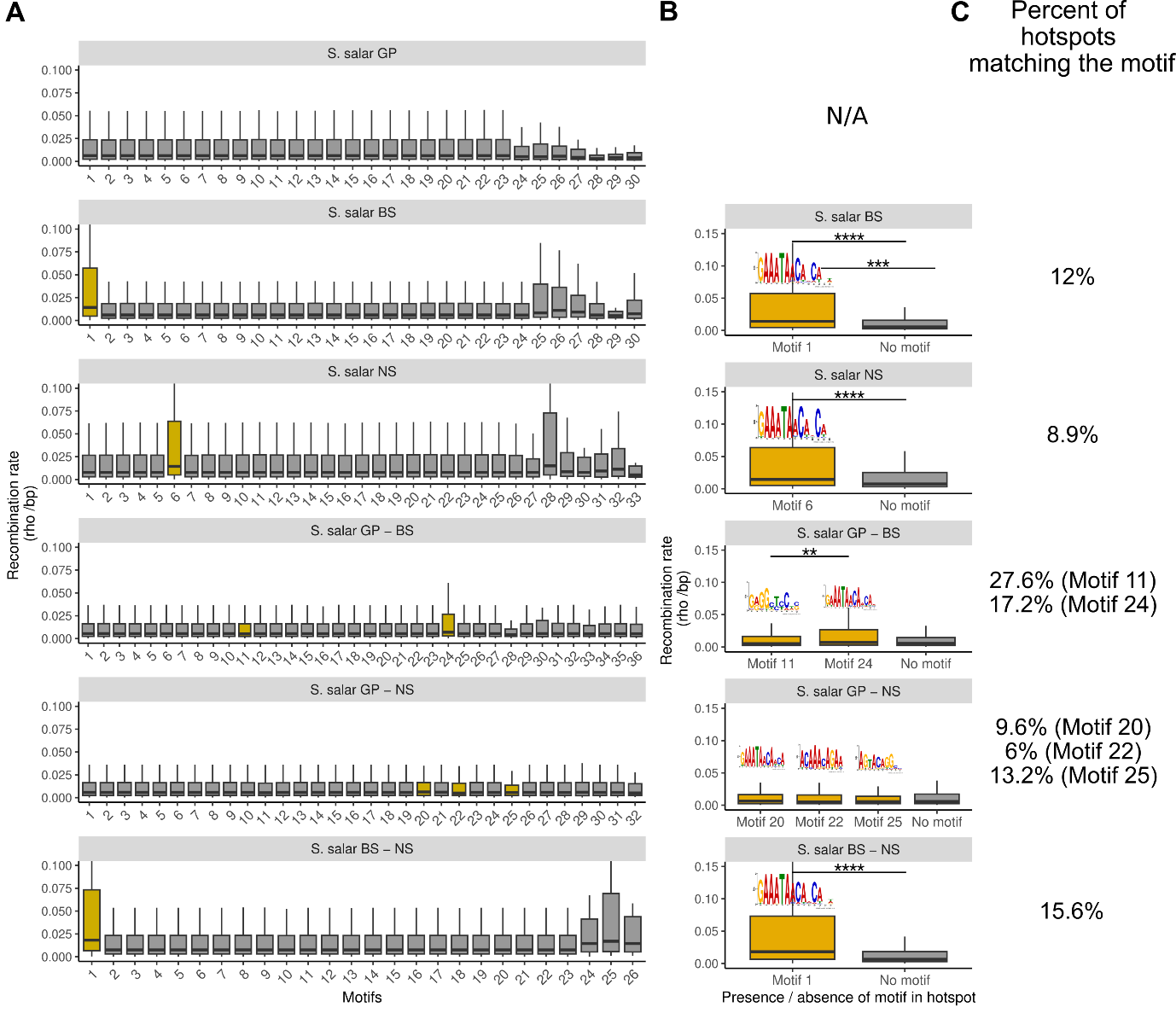
**

**S24 Fig: Motifs enrichment at population-specific and shared recombination hotspots in *S. salar* populations.** **A)** Average recombination rate in motifs found enriched at hotspot. Yellow boxes show motifs found in at least 5% of hotspots showing two-fold enrichment compared to the control set of random spots. **B)** Average recombination rate in hotspots containing the retained motifs from panel A (with the corresponding motifs shown) compared to hotspots not containing the retained motifs. Significant Student’s tests are indicated (***, p-value<0.05). **C)** Percentage of hotspots containing the retained motifs shown in yellow. The data and codes underlying this figure can be found in https://doi.org/10.5281/zenodo.11083953.
