# Supplementary material for: PRDM9 drives the location and rapid evolution of recombination hotspots in salmonid fish
Source: PLoS Biol. 2025 Jan 6;23(1):e3002950. doi: 10.1371/journal.pbio.3002950 (PMC11703093; doi:10.1371/journal.pbio.3002950)
Supplement: S25 Fig — (A) Distribution of PRDM91 (n = 68,047) and PRDM92 (n = 59,986) motifs in rainbow trout genome along chromosomes (paces of 1/30 of chromosome length). (B) Distribution of motifs enriched in the shared hotspots between the BS and NS populations of the Atlantic salmon (n = 936). (DOCX) [file pbio.3002950.s040.docx]

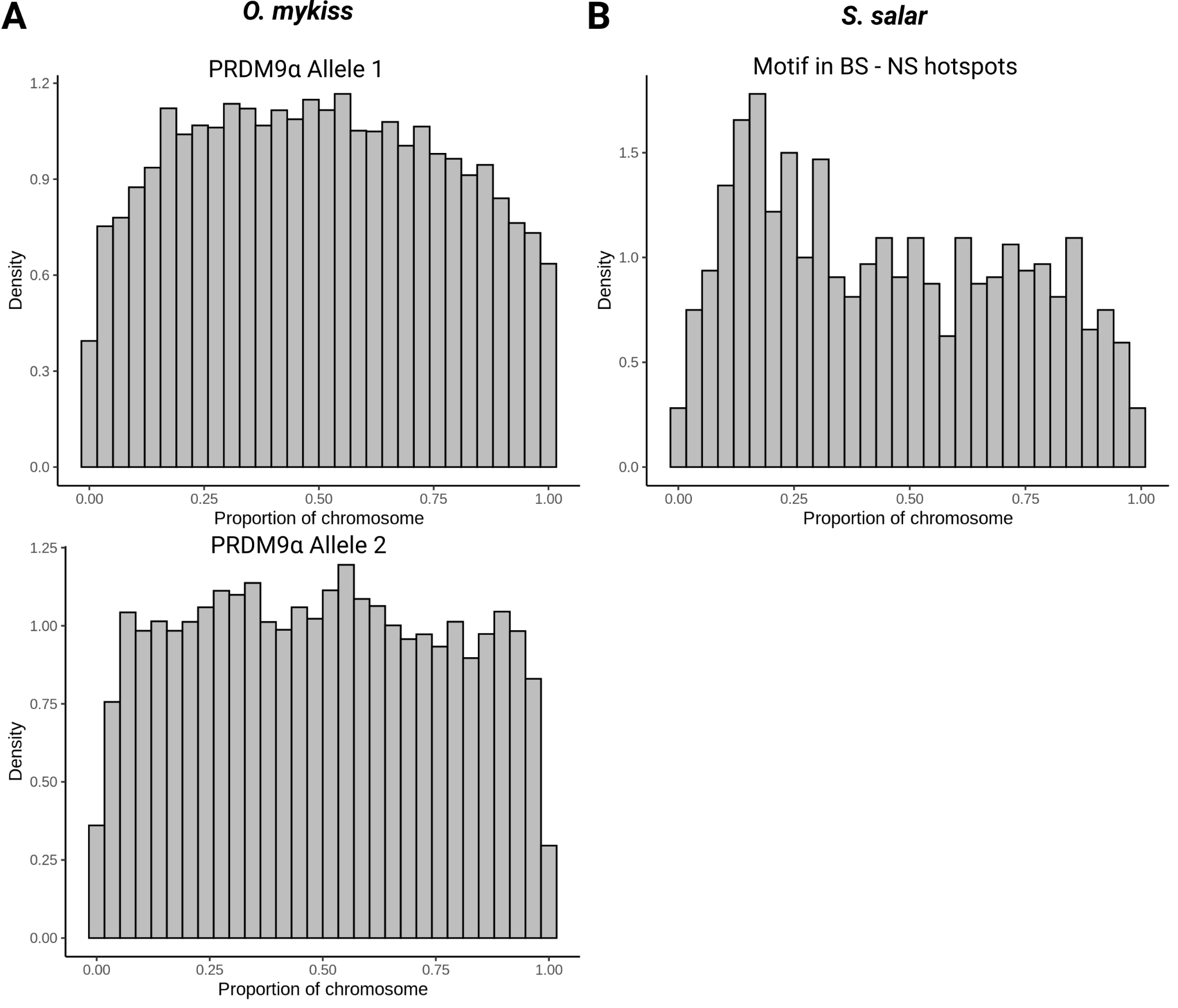


**S25 Fig: Genome wide distribution of PRDM9α motifs along chromosomes in *O. mykiss*. A)** Distribution of PRDM9^1^ (n=68047) and PRDM9^2^ (n=59986) motifs in rainbow trout genome along chromosomes (paces of 1/30 of chromosome length). **B)** Distribution of motifs enriched in the shared hotspots between the BS and NS populations of the Atlantic salmon (n=936). The data underlying this figure can be found in https://doi.org/10.5281/zenodo.11083953.
