# Supplementary material for: PRDM9 drives the location and rapid evolution of recombination hotspots in salmonid fish
Source: PLoS Biol. 2025 Jan 6;23(1):e3002950. doi: 10.1371/journal.pbio.3002950 (PMC11703093; doi:10.1371/journal.pbio.3002950)
Supplement: S26 Fig — (A) Hematoxylin-eosin-stained histological sections of testes from O. mykiss samples used in this study. In TAC-1, TAC-3 and RT-52 the seminiferous tubules were filled with round cells, mostly primary spermatocytes (Sc), some spermatids (ST), few spermatogonia (Sg), and almost no mature spermatozoa visible (Sz). For meiotic cells, between brackets is indicated the substage of prophase I: leptotene (L), zygotene (Z), diplotene (D), and diakinesis (DK). Scale bars are 20 μm. (B) Immunofluorescence of SYCP3, SMC3, and DMC1 in testes sections from a stage III O. mykiss sample not used for ChIP in this study. Scale bars are 10 μm. (DOCX) [file pbio.3002950.s041.docx]

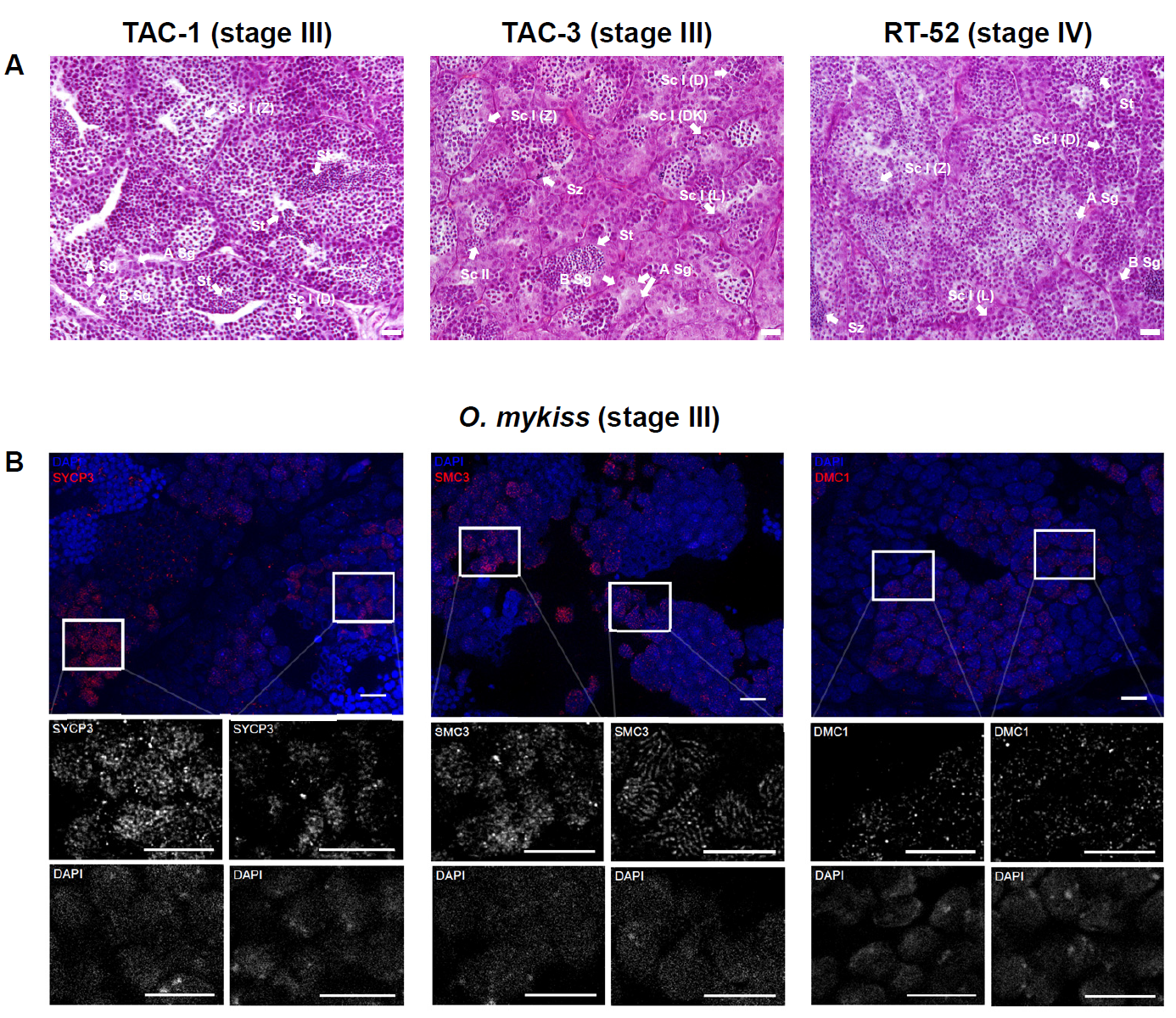


**S26 Fig: Histology and immunostaining of trout gonads. A)** Hematoxylin-eosin-stained histological sections of testes from *O. mykiss* samples used in this study. In TAC-1, TAC-3 and RT-52 the seminiferous tubules were filled with round cells, mostly primary spermatocytes (Sc), some spermatids (ST), few spermatogonia (Sg), and almost no mature spermatozoa visible (Sz). For meiotic cells, between brackets is indicated the substage of prophase I: leptotene (L), zygotene (Z), diplotene (D) and diakinesis (DK). Scale bars are 20μm. **B)** Immunofluorescence of SYCP3, SMC3 and DMC1 in testes sections from a stage III *O. mykiss* sample not used for ChIP in this study. Scale bars are 10μm.
