# Supplementary material for: PRDM9 drives the location and rapid evolution of recombination hotspots in salmonid fish
Source: PLoS Biol. 2025 Jan 6;23(1):e3002950. doi: 10.1371/journal.pbio.3002950 (PMC11703093; doi:10.1371/journal.pbio.3002950)
Supplement: S27 Fig — The 20 individuals of O. kisutch were samples in the Columbia River (in orange) [118], the 22 samples of O. mykiss come from North America rivers (in green) [119], and the 60 individuals of S. salar were sampled in Canada and Norway [120]. Based on population structure analysis, we subdivided the Atlantic salmon samples into 3 populations (in shades of blue): Gaspesie-Anticosti (GP), Barents sea (BS), and North sea (NS). The basemap shapefile used in this figure was derived from the CIA World DataBank II, accessed via the mapdata package in R. (DOCX) [file pbio.3002950.s042.docx]

**
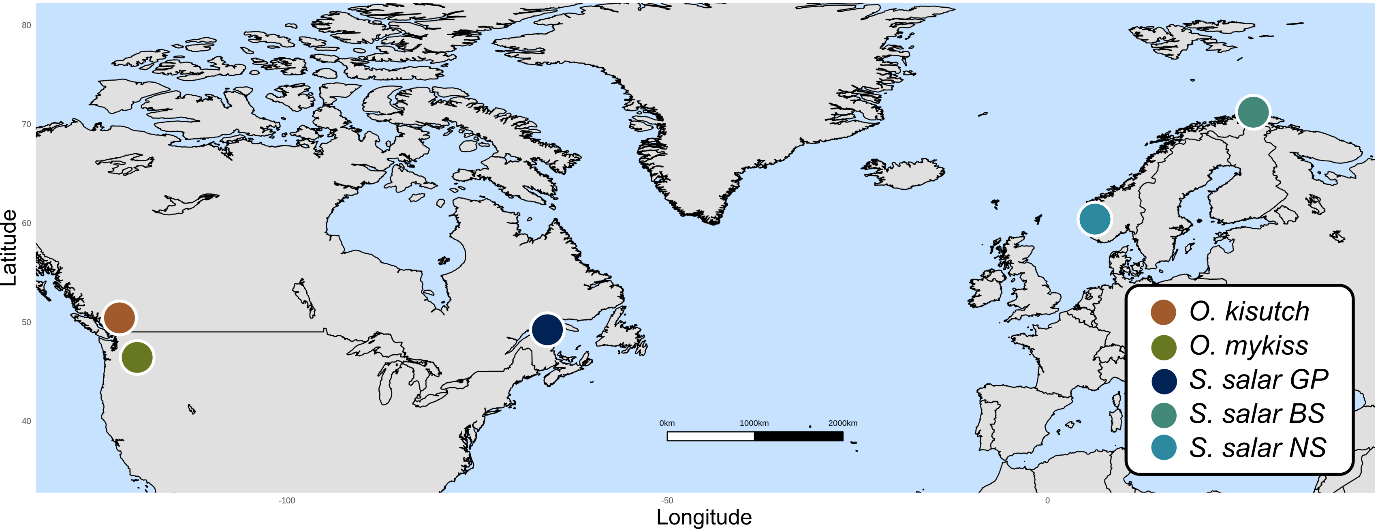
**

**S27 Fig: Sample location.** The 20 individuals of *O. kisutch* were samples in the Columbia River (in orange) (118), the 22 samples of *O. mykiss* come from North America rivers (in green) (119), and the 60 individuals of *S. salar* were sampled in Canada and Norway (120). Based on population structure analysis, we subdivided the Atlantic salmon samples into three populations (in shades of blue): Gaspesie-Anticosti (GP), Barents sea (BS) and North sea (NS). The basemap shapefile used in this figure was derived from the CIA World DataBank II, accessed via the mapdata package in R.
