# Supplementary material for: PRDM9 drives the location and rapid evolution of recombination hotspots in salmonid fish
Source: PLoS Biol. 2025 Jan 6;23(1):e3002950. doi: 10.1371/journal.pbio.3002950 (PMC11703093; doi:10.1371/journal.pbio.3002950)
Supplement: S28 Fig — Spearman’s rank correlation matrix for the 5 populations, p-value <0.05. The data and codes underlying this figure can be found in https://doi.org/10.5281/zenodo.11083953. (DOCX) [file pbio.3002950.s043.docx]

**
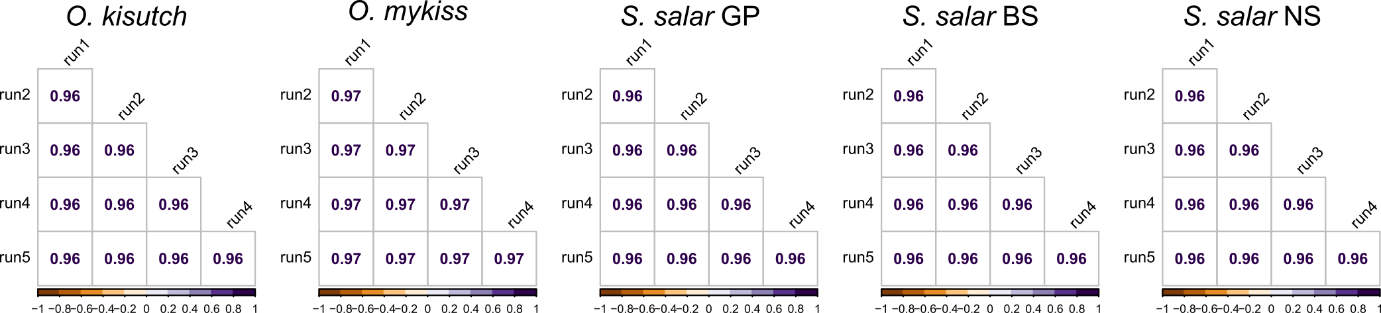
**

**S28 Fig: Pairwise correlation between the five independent runs of LDhelmet.** Spearman’s rank correlation matrix for the five populations, p-value < 0.05. The data and codes underlying this figure can be found in https://doi.org/10.5281/zenodo.11083953.
