# Supplementary material for: PRDM9 drives the location and rapid evolution of recombination hotspots in salmonid fish
Source: PLoS Biol. 2025 Jan 6;23(1):e3002950. doi: 10.1371/journal.pbio.3002950 (PMC11703093; doi:10.1371/journal.pbio.3002950)
Supplement: S29 Fig — (A) Average population recombination rate; (B) average hotspot density; and (C) average population recombination rate in hotspots, according to average sequencing coverage. Student t test p-values and Cohen’s D coefficient are shown in A, B, and C. (D) Fold recombination rates (scaled by the average recombination rate at 50 kb from the nearest feature) according to the distance to the nearest TSS (overlapping or not with a CGI) shown in color, and to the mean depth (shown by the line type). (E) Fold recombination rates (scaled by the average recombination rates in intergenic regions); and (F) hotspot density at the indicated genomic features according to the mean coverage shown in color. TSS and TES were defined as the first and last positions of each gene. CGIs were mapped using EMBOSS with CpGoe > 0.6 and GC > 0. “High” sequencing coverage corresponds to the half of the recombination map with the highest depth and “low” sequencing coverage corresponds to the half of the recombination map with the lowest depth. Only the NS population of S. salar is shown. The data and codes underlying this figure can be found in https://doi.org/10.5281/zenodo.11083953. (DOCX) [file pbio.3002950.s044.docx]

**
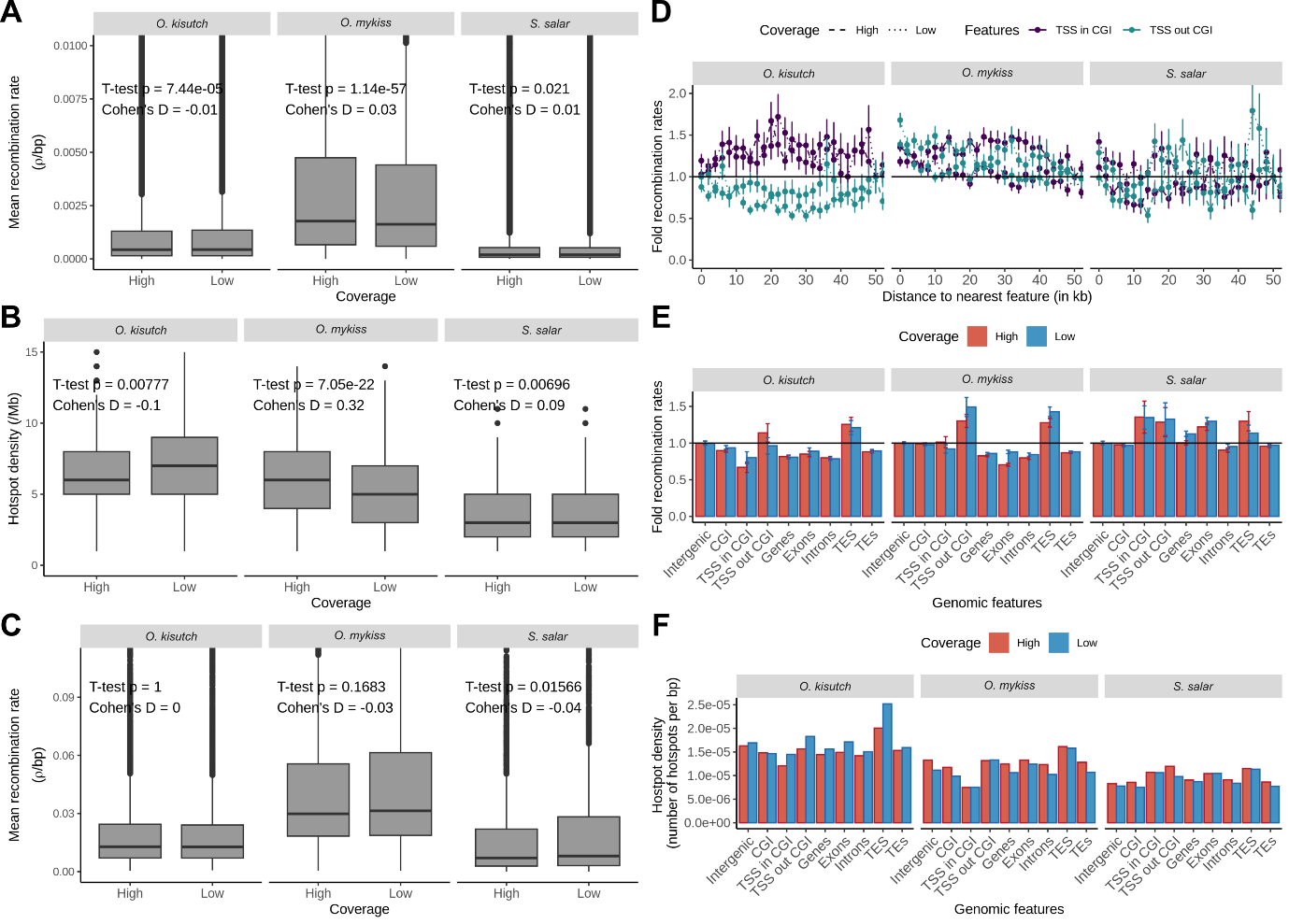
**

**S29 Fig: Patterns of population recombination rate variation controlled for sequencing coverage. A)** Average population recombination rate; **B)** average hotspot density; and **C)** average population recombination rate in hotspots, according to average sequencing coverage. Student t-test p-values and Cohen's D coefficient are shown in A, B and C. **D)** Fold recombination rates (scaled by the average recombination rate at 50kb from the nearest feature) according to the distance to the nearest TSS (overlapping or not with a CGI) shown in colour, and to the mean depth (shown by the line type). **E)** Fold recombination rates (scaled by the average recombination rates in intergenic regions); and **F)** hotspot density at the indicated genomic features according to the mean coverage shown in colour. TSS and TES were defined as the first and last positions of each gene. CGIs were mapped using EMBOSS with CpGoe > 0.6 and GC > 0. "High" sequencing coverage corresponds to the half of the recombination map with the highest depth, and "low" sequencing coverage corresponds to the half of the recombination map with the lowest depth. Only the NS population of *S. salar* is shown. The data and codes underlying this figure can be found in https://doi.org/10.5281/zenodo.11083953.
