# Supplementary material for: PRDM9 drives the location and rapid evolution of recombination hotspots in salmonid fish
Source: PLoS Biol. 2025 Jan 6;23(1):e3002950. doi: 10.1371/journal.pbio.3002950 (PMC11703093; doi:10.1371/journal.pbio.3002950)
Supplement: S30 Fig — Fold recombination rates around hotspots and at orthologous loci in the 2 taxa, for the 2 Oncorhynchus species (A and B), the American (GP population) and European (BS and NS populations) S. salar lineages (D and E), and between the 2 closely related European S. salar populations (BS and NS) (G and H), according to the mean coverage shown in panels. Random expectations (blue) and observed values (orange) of shared hotspots between (C) O. kisutch and O. mykiss; between S. salar populations (F) GP and BS; (I) BS and NS, according to the mean coverage shown in panels. Shared hotspots were defined as 2 kb hotspots overlapping by at least 1 bp. Percent shared is calculated using the number of hotspots in the population with fewer hotspots as the denominator. The expected distribution of shared hotspots has been obtained from 1,000 pairwise comparisons of random spots. “High” sequencing coverage corresponds to the half of the recombination map with the highest depth and “low” sequencing coverage corresponds to the half of the recombination map with the lowest depth. The data and codes underlying this figure can be found in https://doi.org/10.5281/zenodo.11083953. (DOCX) [file pbio.3002950.s045.docx]

**
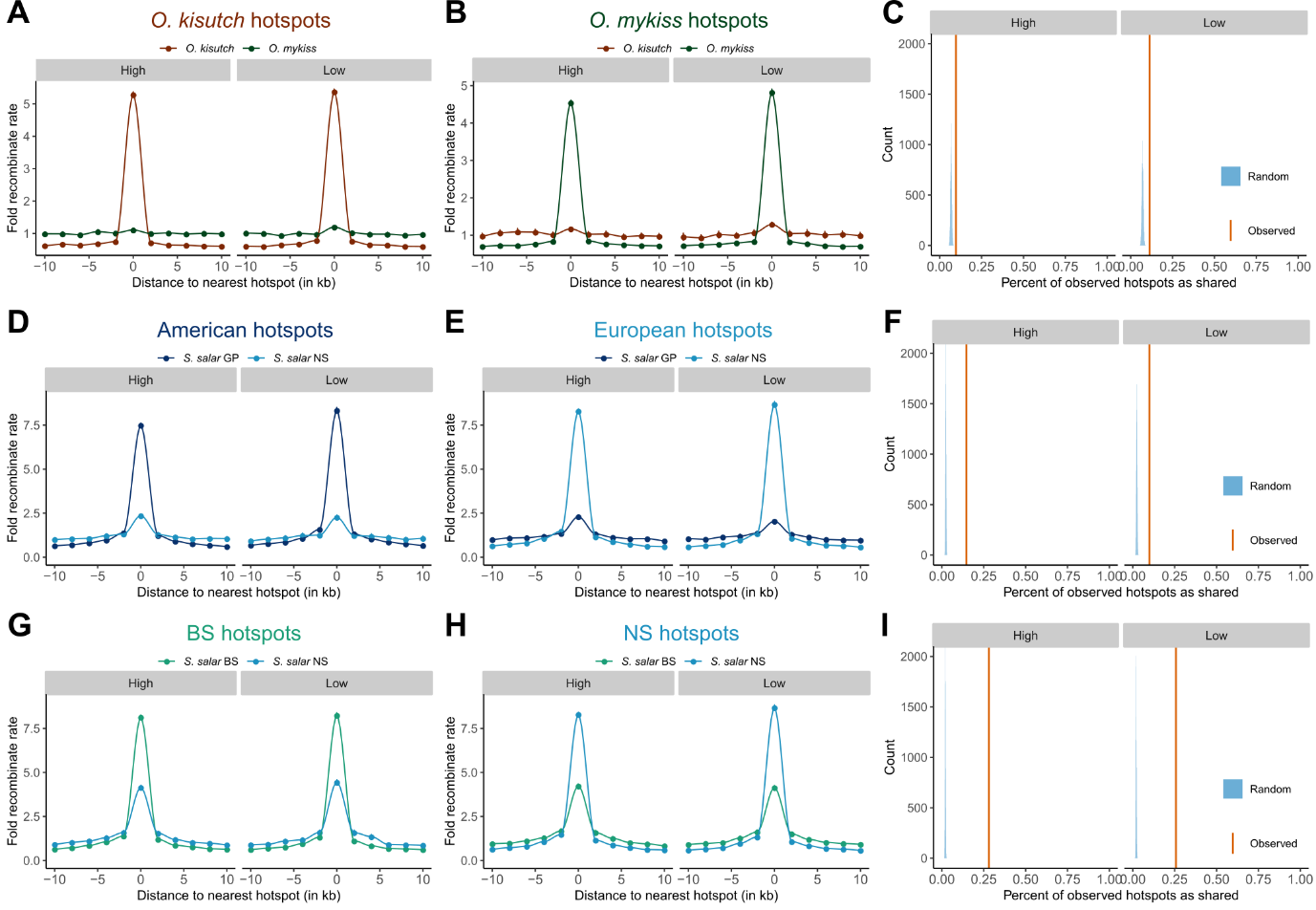
**

**S30 Fig: Hotspot sharing between populations controlled for sequencing coverage.** Fold recombination rates around hotspots and at orthologous loci in the two taxa, for the two *Oncorhynchus* species (**A** and **B**), the American (GP population) and European (BS and NS populations) *S. salar* lineages (**D** and **E**), and between the two closely related European *S. salar* populations (BS and NS) (**G** and **H**), according to the mean coverage shown in panels. Random expectations (blue) and observed values (orange) of shared hotspots between **C)** *O. kisutch* and *O. mykiss*; between *S. salar* populations **F)** GP and BS; **I)** BS and NS, according to the mean coverage shown in panels. Shared hotspots were defined as 2 kb hotspots overlapping by at least 1 bp. Percent shared is calculated using the number of hotspots in the population with fewer hotspots as the denominator. The expected distribution of shared hotspots has been obtained from 1000 pairwise comparisons of random spots. "High" sequencing coverage corresponds to the half of the recombination map with the highest depth, and "low" sequencing coverage corresponds to the half of the recombination map with the lowest depth. The data and codes underlying this figure can be found in https://doi.org/10.5281/zenodo.11083953.
