# Supplementary material for: PRDM9 drives the location and rapid evolution of recombination hotspots in salmonid fish
Source: PLoS Biol. 2025 Jan 6;23(1):e3002950. doi: 10.1371/journal.pbio.3002950 (PMC11703093; doi:10.1371/journal.pbio.3002950)
Supplement: Attachment — Submitted filename: RaynaudSanna_D-24-00615R1_AnswerstoReviewers.docx [file pbio.3002950.s046.docx]

Montpellier, September 25^th^, 2024

Dear editor,

We would like to thank you and the reviewers for your constructive comments. We provide below a detailed response to all the points raised by the reviewers. The original comments are written in blue, our reply in black, with the in-text modifications between commas and italicized. The main points we addressed are:

- We added estimates of Ne, r, and µ/r in Table 1, so that to provide indicators of the capacity of LDhelmet to infer population recombination rates (in response to reviewer #1).
- We performed complementary analyses to ensure that our estimates of recombination rates are robust to limited sequencing coverage (reviewer #1).
- We repeated our analyses using more robust sets of random sequences (controlling for the presence of assembly gaps, SNP density, etc.) to assess the significance of overlaps between hotspots and to search for motifs (reviewer #1 and #2). This did not affect the results.
- We made important changes in the third paragraph of the introduction to give a clearer overview of what was already known regarding the function of PRDM9 outside of mammals (reviewer #1 and #4).
- We analyzed the presence of the genes co-evolving with Prdm9 (ZCWPW1, ZCWPW2, TEX15 and FBXO47) in our four fish species (reviewer #1).
- In the discussion, we added a comparative analysis of fine-scale recombination landscapes across vertebrates (reviewer #4). For this, we combined our data with previously published LD-based maps, thus resulting in a dataset of 18 species (4 birds, 7 mammals, 1 snake and 6 teleost fish; 10 species with a full-length PRDM9, and 8 without).
- We now discuss in more details about the potential function of truncated PRDM9 paralogs (reviewer #4). We also completed Fig.1 to provide more information on the functionality of the different PRDM9 paralogs identified in each species.

As we were working on this latter point of the revisions, we noticed an error: in the submitted manuscript, we had considered that the PRDM9-ɑ2.2 paralog was truncated in *O. mykiss*. But in fact, this gene encodes a full-length PRDM9, with the 4 canonical domains. Our error was due to the fact that we had initially used the genome assembly Omyk_1.0, where Prdm9-ɑ2.2 appears to be incomplete (possibly due to an error in the genome assembly). But a full copy of the gene (XP_036838640.1) is present in the assembly OmykA_1.1 (GCF_013265735.2). Given this error, we decided to double-check our annotations, and found two additional full-length copies (an ɑ2.2 in *S. namaycush*, and an ɑ1.2 in *C. clupeaformis*). We therefore modified our text accordingly (see details in our reply to reviewer #4).

Our changes to the main text are shown in red in the submitted document ("Revised Article with Changes Highlighted").

We added several new figures and tables in the Supplementary material to respond to the reviewer’s comments. We therefore had to renumber these figures and tables. All these changes are detailed at the end of our reply to the reviewers.

We hope you’ll find our revised manuscript suitable for publication in Plos Biology.

Best regards,

Bernard de Massy, Pierre-Alexandre Gagnaire and Laurent Duret

# REVIEWERS' COMMENTS:

## Reviewer #1:

The authors use a combination of experimental and statistical approaches to examine the role of PRDM9 and infer fine-scale recombination rates in four fish species. They provide compelling evidence for the role of PRDM9 in directing double strand break locations in fish that carry an intact version of the gene, and a comparison of recombination landscapes in fish with and without an intact version.

My impression is that the overall conclusions are well supported, but that the comparison between species is hard to interpret as presented. I also believe a few analyses should be revisited-not because I expect the qualitative conclusions to change, but because I think they could be refined.

### Major comments:

1. As the authors appreciate (e.g., https://peercommunityjournal.org/articles/10.24072/pcjournal.254/), maps inferred from patterns of linkage disequilibrium (LD) are shaped by the ratio of recombination rates to mutation rates, the effective population size, and genome coverage, and therefore cannot be taken at face value. Unfortunately, as a result, the comparison presented in Table 1 is hard to interpret. To do so, the reader would need to know, not just the estimate of rho per bp, but the estimates of Ne (or the estimates of r). It would also be good to provide the estimated ratio of recombination to mutation for each species.

We agree : Ne and mutation to recombination ratios (µ/r) are important indicators to assess the power of LDhelmet to infer population recombination rates. We therefore estimated Ne (based on π measured in our resequencing data and on published estimates of µ), and the μ/r ratio (based on r values from published sex-averaged pedigree-based genetic maps). These values (Ne, r, µ/r) are now included in Table 1 (line 398), and we added a paragraph in the Method section (line 981-989) to explain how these values were obtained.

The values of *N*_e_ are in the range for which the LD-based estimate is expected to be reliable (Raynaud et al., 2023), except in *S. salar,* for which lower *N*_e_ values might have led to over-estimate recombination. The *μ*/*r* ratios are relatively low (0.1 to 0.5), which is not optimal for the LD-based approach (Raynaud et al., 2023). Nevertheless, multiple lines of evidence show our LD-based maps provide reliable pictures of recombination landscapes:

- The broad scale LD-based recombination landscapes show a marked increase in recombination toward telomeres (Fig. S10), consistent with pedigree-based genetic maps (and with our DSB map; Fig. S7)
- At fine-scale, LD-based recombination hotspots show a strong enrichment at CGI-associated promoters in the sea bass (in agreement with what has been observed in other fish with a truncated KRAB-less PRDM9ß), but not in salmonids that have a full-length PRDM9 (Fig. 4)
- The fact that LD-based recombination hotspots are not enriched in promoter regions of salmonids (Fig. 4), is confirmed by the distribution of DSB hotspots in rainbow trout (Fig. 3D).
- Rainbow trout LD-based recombination hotspots show a significant overlap with DSB hotspots (Fig. S14)
- The relatively small Ne and *μ*/*r* ratio in *S. salar* are both expected to increase noise in the estimate of recombination rate. Despite this, we found significant correlations between the recombination landscapes of the three Atlantic salmon populations (Fig. 5).

To summarize, we are confident that the patterns of variation we inferred are reasonably robust to the known limitations of the LD-based approach. The new information added to Table 1 should be helpful to present these important parameters (Ne and μ/r ratio) that influence the inference of population recombination rate.

2. If I understood correctly, the salmon samples were only resequenced to an average of 10X coverage (max 13X). This feature should lead to a substantially worse LD-based map, yet to my knowledge is not discussed, let alone analyzed. I would therefore suggest that the authors add all this information to the main text, and evaluate its possible impact on their conclusions, notably for the degree of overlap in Figure 5C.

We agree with the reviewer that limited sequencing coverage may affect the inference of population recombination rates, particularly because it may lead to miss heterozygous positions, thus increasing noise in the LD map. In our original manuscript, to account for the lower coverage in the *S. salar* dataset, we had applied a more stringent missingness filter (lines 821-822 in the original version).

To better control for this effect, we re-analyzed the recombination maps by dividing genome regions into two groups: one with the lowest and one with the highest mean sequencing coverage, as now described in the section ‘*Controlling for a possible effect of limited sequencing coverage*’ of the Supplementary Material.

In all species, the hotspot density and the distribution of recombination rates (genome-wide, or at the vicinity of gene features), was very similar in regions with highest or lowest coverage (S29 Fig). Some weak differences were observed, but in all cases, with negligible effect size (Cohen's D < 2).

Furthermore, the degree of overlap between LD-based recombination hotspots from populations remained broadly similar when comparing hotspots in the lowest and highest coverage regions (S30 Fig). When comparing the BS and NS populations of Atlantic salmon, 25.8% of hotspots from the lowest coverage regions and 28% of those from the highest coverage regions were shared (S30 Fig), whereas in the original analysis we obtained 26.3% of shared hotspots (Fig 5C).

These analyses indicate that low coverage regions do not significantly affect estimated intragenomic variation in recombination rates in our study.

This complementary analysis is now included in the SUPPLEMENTARY MATERIAL (page 13: section ‘*Controlling for a possible effect of limited sequencing coverage*’), and we refer to it in the main text (lines 990-992).

3. On a related note, there is less power to detect hotspots in regions of high recombination, so comparisons of hotspot numbers and heats across the genome need to take that into account. As one example, it is not obvious to me that hotspots are actually hotter in telomeres (lines 344-345); perhaps it is rather that only hotter hotspots are detected?

In the original manuscript, we had written (lines 344-345):

“*Hotspots were evenly distributed along chromosomes, but showed higher recombination rates towards the chromosome ends. This property was observed both for species with a full-length Prdm9 (O. kisutch, O. mykiss and S. salar) and for the species with a truncated Prdm9 (D. labrax)(S16 Fig).*”

But we agree with the referee: we cannot exclude the possibility that we have less power to detect hotspots in highly recombining regions, and hence that the set of hotspots in subtelomeric regions is biased toward hotter hotspots. We therefore removed these two sentences from our manuscript (and we removed the Supplementary Figure S16 that was mentioned only here). This was just a minor point, that does not change the main messages of our paper.

4. A number of the statistical controls could be tweaked. For example, controls for hotspots are generated without regard to the larger recombination rate/genomic context. Similarly, locations are shuffled without regard to genome gaps, minimal diversity levels etc… It may also be better to use actual genome sequences matched for GC content for the motif searching analyses. To be clear, I am not predicting that the qualitative conclusion will change, but these decisions seem somewhat anti-conservative, and should probably be revisited.

This point is related to the 1st comment of reviewer #2.

Following the reviewer’s suggestion, we re-did the hotspot shuffling (to compare the degree of hotspot overlap between hotspot and between ChIP-Seq DMC1 peaks and LD-hotspots and for the motif searching analyses) using a genome mask on the set of control random sequences, to discard regions with the lowest and highest diversity levels, extreme recombination rates, and genome gaps and to exclude hotspots. As anticipated by the referee, this did not affect the results much. We revised Figure 5D, S14 Fig and S20 Fig, and we updated the corresponding values in the result section (line 559):

“*we found a motif that was enriched in 12% of the hotspots of the NS population and 8.9% of the BS population, and in 15.6% of their shared hotspots (Fig 5D).* “

and we modified the Material and Methods section accordingly (line 1053-1061) :

*“We used random permutations to calculate the expected amount of hotspot overlap between the pairs of the three S. salar populations and between the two Oncorhynchus populations. We drew random spots (same number as that of the 2-kb hotspots) 100 times from the genome for each population using Bedtools shuffle, after applying a genome mask to discard the regions with a nucleotide diversity lower and higher than the 2.5 and 97.5th quantile, the 0.1% highest recombination rate values, the 10% larger gap sizes, and genuine hotspots, to control for diversity level, extreme ⍴ values and genome gaps. We compared each of these random sets to those of the compared population to calculate the average overlap expected only by chance.”*

For the motif searching analyses, we did use actual genome sequences matched for GC content, but it appears that we were not clear enough (see also 1st comment of reviewer #2). We therefore modified the Material and Methods section to be more precise (line 1074-1081):

*“In Atlantic salmon, we used STREME from the MEME Suite (141) to find motifs between 10 and 20 bp in length that were enriched at LD-based hotspot positions, compared with control random sequences with a similar GC-content distribution. To obtain these controls, we randomly drew windows (totaling ten times the number of hotspots from the reference genome), and we applied the same genome mask as in the previous section to select random spots, controlling for diversity, high recombination rates and genome gaps and to exclude hotspots. To select a subset of controls matching the GC-content of hotspots, we binned the hotspot GC-content distribution (bin width=0.025) and then drew the same number of random spots for each GC-content bin.”*

We also corrected the section “*Comparison of LD-based landscapes between populations and species*” of the Supplementary Methods (page 13 of the Supplementary Material).

5. This final comment is a matter of opinion, but I thought the framing of the paper was somewhat odd, in making it seem as if we have no idea whether PRDM9 plays the same role outside mammals, when we have evidence that it is active in snakes (cited by the authors) and also evidence that it co-evolves with ZCWPW1 across vertebrates (Cavassim et al. 2022 PNAS; cited elsewhere). In my view, that takes nothing away from the importance of demonstrating that it also directs recombination in fish, especially as this paper presents experimental evidence. In fact, fish are particularly interesting, given the presence of every version of the PRDM9 ortholog within a single taxon.

We agree with the referee (a similar point was raised by reviewer #4): there are already several indirect lines of evidence suggesting that the function of Prdm9 (as described in humans and mice) is likely to be conserved across animals that possess a full-length Prdm9 gene. We made important changes in the third paragraph of the introduction (line 77-106) to mention this point and to better integrate current data in vertebrates including snakes.

Regardless, I think the authors should report whether ZC1PW1 is present and intact across the four fish species studied here (or only the three with PRDM9), and any differences in their PRDM9 SET domain.

We analyzed the presence of the genes co-evolving with Prdm9 (ZCWPW1, ZCWPW2, TEX15 and FBXO47) in our four fish species. The results are mentioned (line 748-750), and detailed in S6 Table:

*“Of note, Zcwpw1, Zcwpw2, and Tex15 are present and intact in the three species that contain a full-length Prdm9 (S. salar, O. mykiss and O. kisutch), but are absent from the genome of D. labrax (S6 Table).”*

We modified a sentence in the result section (line 170-174) to indicate explicitly that the full-length PRDM9 paralogs that we identified in salmonids have conserved catalytic tyrosines in their SET domain:

*“Most of these gene copies only contained a subset of the 10 expected exons and/or showed signatures of pseudogenization (stop codons, frameshifts), but we also identified some complete Prdm9 genes, encoding the four canonical domains, with conserved catalytic tyrosines in the SET domain and without evidence of pseudogenization (Fig. 1). ”*

*D. labrax* only possesses a truncated KRAB-less PRDM9ß copy, and like other PRDM9ß paralogs, its SET domain is mutated at catalytic tyrosine residues. This is now indicated explicitly (line 149-151):

*“The β paralogs contained a complete SET domain (but with mutations at the catalytic tyrosine residues) and a conserved ZF domain, but all lacked the KRAB and SSXRD domains, as previously described (19) (S3 Fig). “*

We also completed Fig.1 and Supplemental Fig. S3 to provide more details on the functionality of the different PRDM9 paralogs identified in each species (presence/absence of exons encoding KRAB, SSXRD, SET and ZF domains, conservation of catalytic tyrosines in the SET domain, presence of stop codon/frameshifting mutations, length of the ZF array). We also provide the corresponding protein sequences and multiple alignments in the Zenodo archive (see section ‘*Data Accessibility’*, line 1097-1103).

### Additional questions/comments:

1. In the phylogenetic analysis, how do the authors convince themselves that the calls are reliable, for example that the copies of alpha 1.2 are actually different?

We agree with the reviewer that phylogenetic relationships are not always very well resolved in all PRDM9 paralog groups. However, as mentioned in the Results sections (lines 130-133 and 143-144 of the original ms, lines 139-143and 154-155in the revised version), we did not only use phylogenetic relationships and node supports to assess the reliability of the paralog groups. We also used the genome position information combined with the orthology/paralogy analysis relationship. Indeed, the information of ohnologous chromosome pairs (i.e., the chromosomes derived from a WGD event) is very well described in Atlantic salmon (Lien et al., 2016), and their orthologous chromosome arms in many other salmonid species were identified in Sutherland et al. (2016).

Concerning the a and b copies of alpha 1.1 and 1.2, we could not precisely establish their phylogenetic relationship, as they appear to have undergone gene conversion, however their consistent positions on the same ohnologous pairs in each salmonid species give us confidence in their calling. We added a new figure in the Supplementary Material (S2 Fig) that shows, for the α1.1 and α1.2 copies, the position of the a and b copies on the scaffolds/chromosomes.

We modified our text (line 155-161) to precise this point:

*“We observed an additional subdivision within the ɑ1 group, with pairs of copies duplicated in tandem present in each pair of ohnologs (i.e. ɑ1.1 a and b and ɑ1.2 a and b, Fig 1A, S7 Table). These duplicated copies are found in almost all species, often having the same orientation (S2 Fig). Although no phylogenetic signal was associated with the a and b copies, probably due to ectopic recombination and gene conversion, these copies are likely to represent a segmental duplication that preceded the Ss4R WGD.”*

2. In the experimental analysis, two salmon (TAC1 and TAC3) that share no PRDM9 alleles have 55 hotspots in common, whereas RT52 and TAC3, which share an allele, have only 42. The authors argue that the 55 are likely not real, but are the 42, e.g., do they show the expected asymmetry of reads?

We thank the reviewer for pointing this question, and we had indeed analyzed the asymmetry of reads. We have modified the figure S7B, and we now show the reads for the three overlapping peak populations: RT-52/TAC-1; TAC-1/TAC-3; TAC-3/RT-52. Whereas the reads from RT-52/TAC-1 and TAC-3/RT-52 overlapping peaks show asymmetry as expected, those from TAC-1/TAC-3 do not.

3. It was unclear to me why the authors chose to use the older version of the motif prediction; do they think it is more reliable? I also wondered if the second motif reported in Figure 3C (which is effectively a series of As) could simply be a nucleosome-excluding sequence.

Sorry for the confusion: although we indeed referred to the in silico prediction in the section of the Supplementary methods, *“Prediction of DNA-binding motif of different Prdm9alpha alleles”* including the references to Persikov 2009 and 2014, we actually did not report any result from these analyses, the reason being that this in silico analysis does not give convincing information. The data we obtained (with the updated version of Persikov et al) is mentioned in our answer to reviewer 4 (comment 10) who also raised this question.

In the revised version, we do not provide information about the in silico prediction and we have thus removed in the “Supplementary methods” the section about “Prediction of DNA-binding motif of different Prdm9alpha alleles”

If the motif identified was simply a nucleosome excluding sequence, it is not easy to understand why it would be an allele-specific hotspot. We note that the current knowledge on DNA sequences influencing nucleosome occupancy is relatively limited. In vivo, it is correct that in S. cerevisiae (but not in S. pombe), polyA tracts tend to be nucleosome depleted, but whether this is conserved is not known. It is not so clear in mammals, maybe due to the fact that for other reasons, promoter regions tend to be GC rich. It remains possible that PolyA have biophysical properties impacting nucleosome occupancy. We thus propose that the most logical interpretation is that this motif is a high affinity motif for Prdm9 zinc fingers.

4. The authors write that "PRDM9 can suppress the recombination activity at chromatin accessible regions (30). Here, we found that this function is conserved in salmonids" (line 562). But unless I missed something, there is no evidence provided for active suppression by PRDM9 in fish-only that rates are not elevated in such regions (e.g., Figure S8 panel C). In that regard, I think it might be worth mentioning in the main text, especially for readers who are not experimentalists, that by looking at DMC1, the data do not strictly reflect double strand frequencies but also potential differences in repair efficiencies.

We agree with the reviewer that our data does not allow us to conclude that there is a suppression of recombination activity at accessible regions. We have rephrased our text (line 612-616):

*“We observe that in Salmonids the presence of PRDM9-dependent hotspots is correlated with a lack of elevated recombination rate at regulatory regions (CGI, TSS or TES) (Fig4 and S6). We suggest that this may reflect an active suppression or competition between the two types of hotspots similarly to what has been observed in mice (30) since TSS and CGIs have elevated recombination rates in D. labrax (Fig 4).”*

We certainly agree that the enrichment of DMC1 depends not only on DSB activity but also on DSB repair (resection and DMC1 lifespan). We thus added this information on line 599-602:

*“One should note that the quantitative level of DMC1 enrichment assayed by DMC1-SSDS can be influenced by the efficiency of DSB repair. If hotspots have variable efficiencies of repair, the quantitative correlation between DSB and LD hotspots could therefore be reduced”.*

5. Why does the species without an intact PRDM9 have much larger hotspots (Figure S15) and less of an increase of recombination rates at the telomeres (Figure S16)? Are these real effects?

\# Hotspot width

The distribution of CO breakpoints at recombination hotspots depends on DSB localization and on end resection. DSBs occurring at PRDM9-dependent hotspots are directed at or very near to the Prdm9 binding sites, at least as reported in mice (Lange 2016). It is in principle possible that at PRDM9-independent hotspots, DSBs are distributed in a larger region, thereby leading to broader hotspots. Notably, the analysis of Spo11 oligos showed that the distribution of DSBs is more narrow in mice (with PRDM9-dependent hotspots) than in budding yeast (that does not have PRDM9) (Lange et al. 2016). However, the width of hotspots detected by the LD-based approach also depends on their intensity and on the SNP density. Hence, with our data, it is difficult to say whether the difference in hotspot width is real, or simply reflects variation in their intensity or detectability by the LD-based approach (the population-scaled recombination rate is about 10 times higher in the sea bass than in salmonids; see Table 1).

\# Recombination rates at the telomeres

Yes, the increase in recombination rate towards telomeres is more pronounced in the three salmonids (3 to 6-fold) than in *D. labrax* (about 2 fold) (see revised S10 Fig). [NB: since we have removed Fig S16 from the revised manuscript, we added a panel to S10 Fig to show chromosomal recombination landscapes across the 4 species].

However, this difference is probably not due to Prdm9. Indeed, in the Threespine Stickleback (a teleost fish, which, like *D. labrax* only has a a truncated KRAB-less PRDM9ß), there is also a strong increase in recombination rate towards telomeres (Shanfelter et al. 2019), similar to what we observe in salmonids (to facilitate the comparison, we included the Stickleback in our S10 Fig, using the data from Shanfelter et al. 2019).

The elevated recombination rate towards telomeres is observed in many animals, and is generally driven by recombination patterns in males. The LD-based maps reflect sex-averaged recombination rates. It is therefore possible that differences in the level of recombination enrichment towards telomeres are primarily driven by variation in the level of heterochiasmy across species.

More data would be necessary to explore these points. At this stage, we do not think it would be useful to speculate on these points in our manuscript. Nevertheless, we added a sentence (line 653-660) to explicitly mention that the telomere-proximal effect is not linked to the presence/absence of a full-length PRDM9:

“*Of note, the increase in recombination rate towards telomeres is more pronounced in the three salmonids (3 to 6-fold) than in D. labrax (about 2 fold), but it is of the same order as in the teleost fish threespine stickleback (61), which only has a truncated KRAB-less PRDM9ß (S10 Fig). […] This telomere-proximal effect appears to be a conserved property, but of variable strength between sexes and among species, independently of the presence/absence of Prdm9 (25, 89, 90).*”

6. In the SOM on p. 16, there is what struck me as a surprising claim: that GC rich CpG islands result from GC-biased gene conversion. What is the evidence for this? How does it explain CpG islands in mammals with PRDM9, which do not recombine much in such genomic locations?

The statement that the GC-richness of CpG islands (CGIs) in mammals and birds results from GC-biased gene conversion (gBGC) is based on analyses of co-variation between substitution patterns and recombination rates across various amniote lineages (Cohen et al. 2011, Auton et al. 2013, Singhal et al. 2015, Kawakami et al. 2017, Hoge et al. 2024, Joseph et al. 2024). We understand the surprise of the reviewer, because most of these papers were primarily focused on describing the properties of recombination hotspots: they reported the fact that hotspots induce gBGC and discussed their overlap with CGIs, but did not explicitly state that the GC-richness of CGIs was a consequence of gBGC. This point is mentioned in the conclusion of our recent paper (Joseph et al. 2024, which was cited as a preprint in the submitted manuscript, but that is now published in PNAS). We modified the text of supplementary material to try to be more explicit (p. 16, first § of the section ‘*Bioinformatic prediction of CpG islands*’) :

*“In mammals and birds, CGIs are also characterized by a relatively high G+C content (Bird 1986). There is now clear evidence that the GC-richness of CGIs in amniotes results from the process of GC-biased gene conversion (gBGC) (Cohen et al. 2011, Joseph et al. 2024). Indeed, in amniotes, substitution patterns in CGIs show an enrichment toward GC specifically in the lineages where they display an elevated meiotic recombination rate, not only in species that lack Prdm9, like dogs or birds (Auton et al. 2013, Singhal et al. 2015, Kawakami et al. 2017), but also in species with an intact Prdm9 (Schield et al. 2020, Hoge et al. 2024). Conversely, in species where recombination is suppressed in CGIs, such as humans or mice, their GC-content is decaying (Joseph et al. 2024).”*

We detail below the reasoning that supports this conclusion.

In principle, different processes may potentially contribute to the high GC-content of CGIs:

-	mutational bias toward GC

-	selection

-	gBGC

It is well established that CGIs are generally hypomethylated in the germline, and hence are less affected by the C->T hypermutability at CpG sites (Cohen et al. 2011). However, the analysis of substitution patterns in primate CGIs showed that their high GC-content is driven by a process that also affects non-CpG sites, and hence that is not simply a consequence of reduced methylation level (Cohen et al. 2011). In fact, several lines of evidence show that gBGC is a major driver of the high GC-content of CGIs.

First, it has been shown that in birds, dogs and snakes, CpG islands correspond to recombination hotspots, and that these recombination hotspots show a GC-biased substitution pattern, as predicted by the gBGC model (Auton et al., 2013; Singhal et al., 2015; Kawakami et al., 2017, Hoge et al. 2024). It should be noted that this GC-biased substitution pattern is observed at non-CpG sites, and hence cannot be attributed to the mutational impact of hypomethylation (Auton et al., 2013; Singhal et al., 2015). Furthermore, the analysis of polymorphism data showed that the GC-biased substitution pattern in these recombination hotspots is driven by a fixation bias, and hence cannot be attributed to mutation (Kawakami et al., 2017).

In principle, this fixation bias might result either from gBGC or from selection. But our recent study clearly rules out the selection hypothesis (Joseph et al. 2024). Indeed, we analyzed substitution patterns in dogs and mice, at loci that correspond to recombination hotspots in Prdm9-KO mutant mice (so-called ‘default hotspots’) (Brick et al. 2012). These ‘default hotspots’ are concentrated in CGIs, and they show a high recombination rate in dogs (that naturally lack Prdm9), but not in wild-type mice (Joseph et al. 2024). Interestingly, we observed that substitution patterns at these loci are biased toward GC in dogs (where they are subject to a high recombination rate) but not in mice (where their recombination rate is low) (see Fig. 3 in Joseph et al. 2024). This pattern fits perfectly with the gBGC model (substitution is biased toward GC specifically in loci/lineages where recombination rate is high). Conversely this pattern invalidates the selective hypothesis: if the high GC-content of CGIs was driven by selection, then one would have expected this selective pressure to affect both dogs and mice. Thus, all these observations are consistent with the gBGC hypothesis, but not with selection or mutation.

*How does it explain CpG islands in mammals with PRDM9, which do not recombine much in such genomic locations?*

The assumption that species with Prdm9 should not recombine in CGIs is based on studies in mice (Brick et al. 2012). But several recent papers showed that the pattern observed in mice is not universal. For instance, snakes, which do have an intact Prdm9, show an enrichment of recombination in CGIs (Schield et al. 2020, Hoge et al. 2024). Earlier work also reported an elevation of recombination around CGIs in great apes (Auton et al. 2012). And in fact, there is evidence that the capacity of Prdm9 to target recombination away from CGIs varies widely across mammals, even between closely related lineages (Joseph et al. 2024). All this indicates that recombination rate at CGIs evolves rapidly. Thus, the high GC-content of mice CGIs might simply reflect that in the past, they had been subject to a high recombination activity. And in fact, the substitution pattern observed currently in mice CGIs indicates that their GC-content is decreasing (Joseph et al. 2024). This non-equilibrium situation probably explains the rapid erosion of CGIs that has been reported in the mouse genome (Jiang et al. 2007).

# Reviewer #2:

I have reviewed the manuscript "PBIOLOGY-D-24-00615_R1" titled "PRDM9 drives the location and rapid evolution of recombination hotspots in salmonids by Marie Raynaud et al."

The authors present a comprehensive examination of the evolution and function of PRDM9 across Salmonids. The findings imply that PRDM9 function and its mode of action has been conserved for at least several hundred million years. Salmonid recombination shows several unifying features to the well-characterized mammalian models of PRDM9-mediated recombination - despite the fascinating genome duplication event in their past. Dependent on the presence of a full-length Prdm9 genotype, there was evidence for rapid evolution of the PRDM9 gene and positive selection on particular amino acids responsible for DNA binding. Furthermore, Meiotic DNA double-strand breaks (DSBs) maps (a proxy for recombination hotspots) concentrated outside promoter regions and displayed abundant H3K4me3 and H3K4me36. Finally, a fast turnover of recombination hotspots brought on by PRDM9 target motif erosion was shown by population-scaled recombination maps. Overall, this is a very nice paper presenting several significant findings. It is the first evidence of PRDM9-mediated recombination features in fishes. It provides an important piece in the puzzle of how conserved the features of PRDM9-mediated recombination are across much longer evolutionary timescales than previously evaluated.

I recommend publication upon minor revision.

### Mayor:

Please clarify:

For the motif-enrichment analyses, you compared real hotspot data, with a control set of random sequences of equal size and GC-content, and I believe I understand how you generated these random sequences. You state (in the methods), "We retrieved a number of random windows equal to the number of hotspots with a similar GC content distribution"… For me to understand the control dataset, could you state the GC content of the real and random sequences and how "similar" it is to the real dataset? Can you perhaps state what the margins are (in % difference)? Also, did you check whether these "random" loci overlap any of the true hotspots? I believe this information to be crucial.

This comment is related to reviewer #1's comment 4.

We modified the Material and Methods section (line 1074-1082) to describe more precisely how we proceeded to obtain a set of control sequences having exactly the same GC-content distribution as hotspots :

*“In Atlantic salmon, we used STREME from the MEME Suite (141) to find motifs between 10 and 20 bp in length that were enriched at LD-based hotspot positions, compared with control random sequences with a similar GC-content distribution. To obtain these controls, we randomly drew windows (totaling ten times the number of hotspots from the reference genome), and we applied the same genome mask as in the previous section to select random spots, controlling for diversity, high recombination rates and genome gaps and to exclude hotspots. To select a subset of controls matching the GC-content of hotspots, we binned the hotspot GC-content distribution (bin width=0.025) and then drew the same number of random spots for each GC-content bin.”*

Following the suggestion of reviewer #1, we applied a filter to exclude control loci overlapping with any of the true hotspots (and also to control for SNP density and genome gaps) (see reply to reviewer #1 comment 4). This did not affect the results.

Graphical representation of the Supplementary material:

While, in general, the quality of the graphical representations was high in the main body of the manuscript, I was disappointed with the quality of some of the Supplementary Figures. Even though they display exciting findings, they miss the level of detail awarded to the main body of the text and seem to be rather poorly put together. There are several minor and not-so-minor problems that I have listed below:

We are sorry for the poor display of some figures. We have identified the problem, it was due to a change in format during the pdf conversion. All figures have been checked and revised when necessary.

In Figures S10 and S11, Legend and Title overlap, making the legend hard to read.

Figures S10 and S11 (now S8 and S9) have been revised.

S15, A, and S16 in A: could the number of hotspots be brought on the same scale? Ie. For S15 ~15000 for both OKIS and OMYC and 4000 for the three Salmo salar, 400 for DLAB and the differences explained somehow? It's curious why the "outgroups" to Salmo salar have more than 3-fold difference in number of hotspots. And for S16, a maximum of 250 hotspots on the y-axis would fit all the data.

Yes, we detected about 2 to 3 times more hotspots in salmonids than in *D. labrax* (Table 1). This reflects differences in genome sizes (0.6 Gb for *D. labrax*; 1.7 Gb to 2.5 Gb in salmonids; see Table S4). This point is now mentioned in the discussion (line 627-629):

*“By LD-based approach, the number of hotspots detected is 2 to 3 times higher in the three salmonid species than in D. labrax (Table 1), but this difference is mainly explained by their larger genome sizes (1.7 to 2.5 Gb, compared to 0.6 Gb for D. labrax).”*

To facilitate comparison, we now plot the proportion of hotspots (instead of the number of hotpots) in the Figure S15 (now S13). Figure S16 has been removed (see reply to Reviewer #1 Comment 3).

S17: "comparaison" should probably read "comparison", and "expectated" should probably read "expected."

Corrected

Figure S18: comparisons between species are on different scales. i.e. O. mykiss, S. salar NS, BS and GP all have the max value of 4000 shown on the heatmap scale, and O kisutch has 3000. So, the heatmap is hard for me to interpret when all species' graphs for each variable (GC content, SNP density, etc.) are not on the same heatmap scale. I would find it helpful if the X-axis were on the same scale, i.e. -6 to 0 for the Recombination rate. In a perfect world, Y-axes would also match, but I find it understandable that the Y-axis has different scaling. Similarly, I have a hard time interpreting whether the TE density is different in O mykiss and O kisutch compared to the S. salar species, or whether the difference of the shape is driven by plotting at a different scale. In the same context, it would be nice if the Spearman rho values were all on the same horizontal line

Thank you for your suggestion, the figure has been modified accordingly.

Figure S19, at least SNP density could be put on the same scale, and all plots could have the same height; the legend for the S. salar populations would still fit if the plots were the same height. S23 lacks the top ruler number (3000).

Thank you for your suggestion, the figure has been modified accordingly.

The same is seen in Figure S25, where the Frequency of PRDM91 and PRDM9 motifs are shown on different scales,

on Figure S25 (now S22), the scales of Y axes have been homogenized.

S24, again, the n_neighbors heatmap is on various scales (0-6500) in one, (0-6000) in the other.

Corrected.

In Figure S26, the probability values are on different scales, from 0.000000 to 0.000100 (six decimals) in one graph and go up to 0.00025 (shown in 5 decimals) in another. The spacing and the numbers on the ruler also differ between graphs. This is again making comparisons extremely difficult to grasp visually. Please unify as much as possible. The p-value also overlaps with the X-axis, making it hard to read.

In Figure S26 (now S23), we have edited as much as possible axis numbering and edited the text to make p-values readable.

### Minor:

Line 37: … in turns… Do you perhaps mean "in turn"? This phrase could also be deleted as such: "It increases genetic diversity by creating novel allele combinations (4, 5) that facilitate adaptation and the removal of deleterious mutations from natural populations (6-8)."

Corrected

Line 41: "Broad-scale patterns of variation within chromosomes (megabase scale) have…"

Perhaps consider rephrasing to "…(at the megabase scale)...

Corrected

Lines: 50 - 55: how about in canids? Also, the sentence starting in line 52, "In vertebrates…" is misleading, as only "default" "recombination hotspots are associated with TSSs that are located within CpG islands" in some vertebrates. But this is not generally true for all vertebrates, as you also pointed out later. Please rewrite this sentence to clarify the fact.

We modified this sentence: “*In dogs and birds, recombination hotspots are particularly associated with TSSs that are located within CpG islands (CGIs).*”

Line 100: "here" may be unnecessary in this context; to me the sentence makes more sense as "To this aim, we investigated…"

Thank you for the suggestion, revised.

Line 574 …". This telomere-proximal effect appears to be a conserved property, but of variable strength between sexes and among species with/without Prdm9"

I think it's important to note that telomere proximal enrichment of recombination hotspots is seen in humans and has been seen in dogs and birds as well (that lack PRDM9), including wild birds.

Thank you for the suggestion. We have added references for those species not mentioned in the review by Zelkowski et al, i.e. dogs (Campbell 2016) and birds (Singhal 2015). We also rephrased this paragraph (see response to reviewer #1, additional comment 5).

Line 609: "… suggesting that PRDM9 may be limiting in some contexts."

Do you mean that "PRDM9 dosage" may be limiting? Please clarify

Yes, we mean PRDM9 protein level. We modified that sentence: “*suggesting that PRDM9 protein level may be limiting in some contexts*”

977: I believe the name is Rajalekshmi Navaryana Sarma, not "Sarna."

Thank you, revised

# Reviewer #3:

The work by Raynaud et al., is a straightforward investigation on the determinants of meiotic recombination in salmonids. They are a diverse family of teleost fish, where, crucially, a full length copy of the protein PRDM9 has been found. PRDM9 controls the location of recombination hotspots in mammals, though it has been recently shown that it can direct recombination in vertebrates. PRDM9 has a fascinating history of losses through evolution therefore it is of interest to characterize the recombination landscape in other non-mammalian taxa. Raynaud et al., analyzed the evolutionary dynamics of PRDM9 across the phylogeny of salmonid, identified the sites of recombination initiation in the rainbow trout and inferred historical recombination rates from patterns of linkage disequilibrium (LD) in five populations from three different species. The authors convincingly show that PRDM9 controls recombination hotspot activity in salmonids and that it is the predominant pathway utilized in these fish. Interestingly they also describe a PRDM9-independent pattern of elevated double-strand break formation and recombination in the telomere-proximal region, similarly to what was described in mammals.

Altogether, this is a very comprehensive and well-executed set of experiments that fully support the major conclusions of the paper. I recommend its publication without reservations as it provides an important piece in the puzzle of how recombination arose and is controlled in and beyond mammals.

A minor comment:

The PRDM9 binding motif derives is not GC-rich as it is in mammals. The authors also show that the GC content is not elevated at recombination hotspots (in a fine-scale). Any thoughts on how these things can be related, and whether GC-biased gene conversion is not as active because of the genomic context and not due to some intrinsic mechanistic differences?

There are few species for which the binding motifs of PRDM9 have been determined experimentally (i.e. identified in allele-specific DSB hotspots and not simply predicted based on the sequence of the zinc finger). In humans, the two major classes of alleles (A-type and C-type) have very GC-rich targets (Fig. 5A in Alleva et al. 2021). In mice, the target motifs of the six studied alleles show an intermediate GC-content (~50%, see Fig. 1E in Smagulova et al. 2016). The motifs we identified in rainbow trout are more AT-rich. As mentioned in our manuscript, salmonid genomes do not appear to be affected by GC-biased gene conversion (gBGC), contrarily to mammals. However, with only three species, it is difficult to draw any conclusion regarding the correlation between the intensity of gBGC and the GC-content of PRDM9 binding motif.

A priori, we see no direct connection between these two features, but one could imagine an indirect link. Indeed, in genomes that are subject to gBGC, regions of high recombination tend to be GC-rich. Hence, during the turnover of PRDM9 alleles induced by the Red Queen, alleles that target GC-rich motifs might confer a higher fitness, and hence be preferentially selected. But data from more species would be necessary to test this hypothesis, and at this stage, it seems to us that it is premature to speculate on this point.

We have no clue of the reason why gBGC is so weak in salmonids. More generally, it is not known why the intensity of gBGC is very strong in some taxa but weak in others. It is known that the intrinsic intensity of gBGC (measured as the proportion of conversion events at G:C/A:T heterozygous site that lead to the transmission of the G:C allele) varies among species: from 50.6% [95% CI 50.3,51.0] in budding yeast (Mancera et al. 2008), to 59.0% [52.3,65.4] in flycatchers (Smeds et al. 2016), 59.9% [56.6,63.2] in mice (Li et al. 2019), and 67.9% in humans [66.3,69.6] (Halldorsson et al. 2016). This variation probably results from differences in the DNA repair machineries that are at play during meiotic recombination. In addition, variations in the length of heteroduplex, in recombination rate and in effective population sizes are also expected to contribute to differences in the intensity of gBGC.

# Reviewer #4:

In this manuscript entitled "PRDM9 drives the location and rapid evolution of recombination hotspots in salmonids", Raynaud et al. analyzed the meiotic recombination in different species of salmonids with a focus on the impact of Prdm9 allelic background. The authors used fine-scale recombination maps based on DMC1 ChIP sequencing and estimation of population-scaled recombination rates to present descriptive data of several salmonids. This is combined with an in silico analysis of the genomic features associated with hotspots. Authors conclude that PRDM9 determines location of recombination hotspots, leading to evolutionary signatures on those sites such as rapid motif erosion and turnover of recombination hotspots, resembling previous described patterns in model mammalian species.

Authors provide a nice introduction with relevant background material and the results are clearly written with well-organized sections, making it easy to interpret. Overall, results presented are compelling, being of broad interest to the meiosis community, especially for those working in non-model species. However, conclusions are not entirely novel, beyond the description of a conservation of patterns already described in mammals. Perhaps it will be desirable to make more emphasis on those patterns that are not common among mammals (such as mice or humans). In relation to that, authors might extend their interpretations on the data of truncated Prdm9 paralogs, providing additional detail on the possible implications.

We thank the reviewer for his/her comments. We would like to emphasize that one question raised by studies in mice and humans was whether the function of PRDM9 in hotspot localization would be conserved outside of mammals. We have revised the third paragraph of the introduction (also following reviewer 1 suggestion) to better introduce the context and the question about the conservation of Prdm9 function outside of mammals. In our study, the novelty is that we show by molecular analysis that the molecular activity (i.e. sequence-specific DNA binding and histone modification) of PRDM9 is conserved in Salmonids, and this is the first demonstration for this conservation outside of mammals.

The properties of recombination hotspots in salmonids are similar to those of PRDM9-dependent hotspots in mammals, with the exception of the GC biased gene conversion (see also our answer to the comment by Reviewer 3 above). What is maybe more interesting is that thanks to our data, it is now possible to investigate the impact of the presence of PRDM9 on fine-scale recombination landscapes across a broad set of vertebrate species (see below).

In relation to that, authors might extend their interpretations on the data of truncated Prdm9 paralogs, providing additional detail on the possible implications.

We agree with the reviewer that the presence of truncated Prdm9 paralogs raises interesting questions regarding their functionality. We added a section in the discussion to develop this point (line 686-715):

## ***“Functional divergence of PRDM9 paralogs***

*Another intriguing pattern revealed by our study is the complex duplication history of the Prdm9 gene in salmonids, shaped by WGD events and by segmental duplications. Some of these duplications led to functional innovations. Notably, the two major PRDM9 clades (α and ß) resulted from the Ts3R WGD in the ancestor of teleost fish (19). PRDM9-ß lacks the KRAB and SSXRD domains, and is mutated at the catalytic residues of its SET domain (19). The function of PRDM9-ß has not been characterized, but the fact that this protein is strongly conserved across teleost fish, including salmonids that have a full-length PRDM9-α (S1 Fig), implies i) that it is functional, and ii) that its function is not redundant with that of the canonical full-length PRDM9. Interestingly, the salmonid-specific WGD generated two PRDM9-ß paralogs (ß1 and ß2) that are well conserved across all salmonids (S1 Fig), which indicates that they both are under purifying selection.*

*In contrast to the conservation of PRDM9-ß paralogs, the duplications of PRDM9-α genes led to many copies that are truncated or show evidence of pseudogenization (Fig 1). The first segmental duplication generated the α1 and α2 clades. All salmonids (12/12) have one full-length copy in the PRDM9-α1 clade (in the subclade α1.1 in some species, α1.2 in others, except C. clupeaformis that has retained both α1.1 and α1.2). Conversely, only two species have retained a full-length PRDM9-α2 paralog (α2.2 in O. mykiss and S. namaycush). In all other species, the KRAB domain of α2.2 is missing or pseudogenized (Fig 1). The analysis of published RNAseq datasets showed that PRDM9-α2.2 is expressed at high level in testis, both in O. mykiss (where it is full-length) and in O. kisutch and S. salar (where it is truncated). The SET domain of α2.2 contains the three conserved tyrosine residues important for methyltransferase catalytic activity (95) (Fig 1, S4 Fig). But PRDM9-α2.2 genes show little diversity at their ZF domain, which suggests that unlike full-length PRDM9-α1, it is probably not involved in directing recombination. It is possible that those paralogs contribute together with the full length PRDM9 to hotspot activity. For instance, they may have retained putative protein interaction properties through the SSXRD domain or some zinc fingers, and may also be able to oligomerize with PRDM9 as proposed for mouse PRDM9 (96). It is also possible that they have no function in hotspot activity, but play a role in the regulation of gene expression as some members of the PRDM protein family do (97).“*

We also completed Fig.1 and Supplemental Fig. S1 to provide more details on the functionality of the different PRDM9 paralogs identified in each species (presence/absence of exons encoding KRAB, SSXRD, SET and ZF domains, conservation of catalytic tyrosines in the SET domain, presence of stop codon/frameshifting mutations, length of the ZF array).

As we were working on the preparation of this new Fig. 1, we noticed an error: in the submitted manuscript, we had considered that the PRDM9-ɑ2.2 paralog was truncated in *O. mykiss*. But in fact, this gene encodes a full-length PRDM9, with the 4 canonical domains. Our error is due to the fact that we had initially used the genome assembly Omyk_1.0, where Prdm9 alpha 2.2 appears to be incomplete (possibly due to an error in the genome assembly). But a full copy of the gene (XP_036838640.1) is present in the assembly OmykA_1.1 (GCF_013265735.2). Given this error, we decided to double-check our annotations, and found two additional full-length copies (an alpha 2.2 in *S. namaycush,* and an ɑ1.2 in *C. clupeaformis*). We therefore corrected the first section of the results (line 170-1798:

*“Most of these gene copies only contained a subset of the 10 expected exons and/or showed signatures of pseudogenization (stop codons, frameshifts), but we also identified some complete Prdm9 genes, encoding the four canonical domains, with conserved catalytic tyrosines in the SET domain and without evidence of pseudogenization (Fig. 1). In the ɑ1 clade, we detected on average 3.8 paralogs per genome, but each species retained only one full-length copy (corresponding to the ɑ1.a.1 paralog in Thymallus, Oncorhynchus and Salvelinus, and to the ɑ1.a.2 paralog in the two Salmo species), except in C. clupeaformis, where both ɑ1.1 and ɑ1.2 are full-length. Conversely, in the ɑ2 clade, we detected a full-length copy in only two species (ɑ2.2 in O. mykiss and in Salvelinus namaycush).”*

And we also corrected the 2nd section of the results (line 243-262):

*“In addition to the full-length α1 copy, we observed that the α2.2 paralog is also strongly expressed in testes, both in Oncorhynchus and in Salmo genera. This paralog is full-length in O. mykiss and S. namaycush, but in all other salmonids the KRAB domain of α2.2 is missing, or pseudogenized (Fig. 1). This phylogenetic pattern implies that α2.2 lost its KRAB domain several times independently, in different lineages. In S. salar, the allelic diversity of the ZF array in the truncated Prdm9 α2.2 was very low: in the 20 individuals analyzed, we observed one single allele where the array had 5 ZF units (S3 and S5A Figs). This is consistent with the hypothesis that KRAB-less PRDM9 homologs lost the capacity to trigger recombination hotspots, and therefore are no longer subject to the Red Queen dynamics (19). The proportion of amino acid diversity at DNA-binding residues is relatively high among the 5 ZFs of this unique PRDM9 α2.2 allele (r=0.471). The persistence of this signature of positive selection suggests that the functional shift associated with the loss of the KRAB domain is relatively recent. In O. mykiss, where PRDM9 α2.2 is full-length, we identified five α2.2 alleles in 20 individuals, with 6 to 12 ZFs (S5A-B Fig). Some ZFs lost one amino acid, with unknown consequence on their DNA binding capacity (S3 Fig). The proportion of amino acid diversity at DNA-binding residues was lower in the truncated PRDM9 α2.2 (r=0.367, S5C Fig) than in the full-length PRDM9 α1.a.1 (r=0.552, Fig 2C). This observation, together with the relatively limited allelic diversity, suggests that O. mykiss PRDM9 α2.2 is not subject to the Red Queen dynamics, and hence that it has lost its function of directing recombination, like the KRAB-less α2.2 paralogs in other salmonids.”*

### Major comments

1. All throughout the work (see lines 255-265 or 340), authors compare their results on salmonids species with mice/humans recombination hotspots. However, the number of peaks in SSDS are considerably low compared to those detected in mice or humans. This needs clarification. Would it be related to a limitation of the technique when used in salmonids, including the use of different antibodies for different animals/species? Also, the number of detected hotspots based on LD are much higher. How can the authors reconcile these contrasting results?

The number of hotspots that we detected by the LD-based approach in salmonids (~20,000; Table 1) is of a similar order of magnitude to what has been reported in humans (~33,000; Frazer et al., 2007) or mice (~39,000; Booker et al., 2017). However, the number of DMC1-SSDS peaks that we detected in rainbow trout (~200 to 1,900 per individual) is much smaller than what has been reported in humans (~20,000 to 30,000 peaks per individual, Pratto et al. 2014) or mice (~10,000 to 20,000 peaks per individual, Smagulova et al. 2016)

As the reviewer pointed out, there are several potential technical parameters that can influence DSB hotspot detection.

In terms of antibody, we do not have a quantitative comparison between the antibody used here and the one used in mice, however, qualitatively (western blot assays not shown), our anti-DMC1 antibody gave satisfactory results.

One difference with humans and mice, is that in salmonids, spermatogenesis is developmentally regulated. We selected males undergoing a wave of spermatogenesis as determined by the histological stages (III or IV; as shown in the histology data now provided as S26 Fig, also requested by the reviewer in comment 11). However, even at these stages, the percentage of spermatocytes undergoing DSB repair in salmonids may be lower than on the samples used in mice or humans, which could explain the lower number of detected peaks.

2. Related to the previous point, pertinent comparisons with patterns found in non-mammalian species (such as snakes or birds) are missing. Also, there is not any mention of studies in meiotic recombination from other fishes.

We agree: we combined our recombination maps with previously published maps, to obtain a dataset of 18 species (4 birds, 7 mammals, 1 snake and 6 teleost fish). Based on this dataset, we compared fine-scale recombination landscapes in species with or without PRDM9. We added a section in the discussion to develop this point (line 629-647):

*“To get a broader view of the impact of PRDM9 on vertebrate fine-scale recombination landscapes, we combined our data with previously published LD-based maps, thus resulting in a dataset of 18 species (4 birds, 7 mammals, 1 snake and 6 teleost fish; 10 species with a full-length PRDM9, and 8 without; S5 Table). On average, the hotspot density is about 2 times higher in genomes with a full-length PRDM9 (8.6 hotspots/Mb) than without (4.4 hotspots/Mb; t.test p-value=0.056). However, it is difficult to directly compare these numbers, because different studies used different criteria to define hotspots. To get a more comparable estimator of the heterogeneity of recombination landscapes, we measured the fraction of recombination events occurring in the 20% of the genome with the highest recombination rate. On average, in genomes with a full-length PRDM9, 84% of recombination is concentrated in 20% of the genome, compared to 70% in genomes without (t.test p-value=0.011). Data from more species would be necessary to control for phylogenetic inertia. However, this preliminary observation suggests that recombination is more concentrated into hotspots in species having a full-length PRDM9. Of note, the LD-based approach measures the population-scaled recombination rate, integrated over many generations, and hence is expected to reflect the historical diversity of PRDM9 alleles. It is therefore likely that in species with PRDM9, the recombination landscapes of individuals are even more heterogeneous than what can be measured by the LD-based approach.*”

3. Limited reference is made to the differences in intensity of hotspots, particularly on those shared or specific per alleles (they compare intensities when comparing hotspots from SSDS and LD at line 354, or in the discussion-Line 575-, but not between the studied species/populations)

We are not sure to understand the point that the referee wants to raise. The aim of the comparison presented in line 354 was to test whether the overlap between LD-based hotspots and SSDS peak was really meaningful, or if it might have occurred simply by chance (in which case, the overlapping hotspots would have had no reason to be more intense).

The comparison of the intensity of SSDS peaks that are shared between individuals vs specific to an individual would show that shared-peaks are on average stronger. But this simply reflects the fact that weak peaks are more likely to be missed (false negatives) and hence to be individual-specific. We do not see what information this would add to our manuscript.

4. Discussion on the genomic features characterizing independent-prdm9 hotspots is particularly absent.

We did not discuss much about the features of Prdm9-independent hotspots because i) this is not the central topic of our paper and ii) our data do not add novel insight on this specific set of hotspots (data from *D. labrax* confirm previous studies showing that in fish species with a truncated KRAB-less PRDM9, recombination is enriched at TSS and CGIs). We mention in the discussion that “*TSSs and CGIs have elevated recombination rates in D. labrax”* (line 615). We also summarized our current knowledge by saying that (line 620): "*In the absence of PRDM9, hotspots occur in accessible regions of the chromatin such as promoters, enhancers or other regulatory regions and CGIs*".

5. It would be interesting to pointing out genomic features of independent-prdm9 hotspots. It was especially absent in the discussion.

See answer above

6. Main figures could benefit from a clearer focus to strength the manuscript. For example, figures can be more homogeneous when considering aesthetics; figure 4 seems differently produced in terms of font size and style than the rest.

We agree with the reviewer. We have reformatted and homogenized the figures.

### Additional comments:

7. Only GC-biased gene conversion is not a common pattern between salmonids and mammals-birds (Lines 405-409), is that right?

Yes, indeed we detected very weak gBGC in salmonids (see also our reply to the comment of Reviewer 3).

8. Regarding the recombination of tetraploid genomic regions is commented on the LD data. Have they compared the number of recombination hotspots in SSDS derived data? (Lines ~422-435).

Following the suggestion of the reviewer, we compared the density of DMC1-SSDS peaks in chromosomes with or without tetraploid regions, for each of the three studied individuals :

**Figure R4.1: DMC1-SSDS peak density tends in chromosomes containing tetraploid regions (yellow) and in chromosomes without tetraploid regions (grey).**

This figure shows that in TAC-1 and in RT-52, the DMC1-SSDS peak density is 10%-30% lower in chromosomes containing tetraploid regions (4N chromosomes, yellow) than in chromosomes without tetraploid regions (2N chromosomes, grey). We observed no difference in TAC-3 (possibly due to the limited number of peaks; N=209 peaks).

This difference might reflect differences in DSB hotspot intensity or differences in the kinetics of DSB repair (resulting in differential SSDS signal accumulation per DSB event) between diploid and tetraploid regions. However, this difference might also be driven by an artefact: sequence reads that map on tetraploid regions are more likely to be filtered out (because they map at multiple sites), and hence we may have less sensitivity to detect DSB hotspots in these regions.

It should be noted that in the LD-based map of *O. mykiss,* the population-scaled recombination rate (rho=4Ne.r) appears to be significantly higher in 4N chromosomes compared to 2N chromosomes (S18 Fig). There are several possible explanations for this discrepancy between observations made on LD-based recombination maps vs DSB maps. First, in the LD map, the higher rho in 4N vs 2N chromosomes might simply be explained by a higher Ne on tetraploid regions rather than a higher recombination rate (r). Second, it is possible that DSBs on 4N chromosomes are more prone to be repaired as COs than those on 2N chromosomes.

We think that at this stage, it is not possible to draw any clear conclusion based on our observations. We therefore did not include this analysis in the revised manuscript.

9. Line 467: Authors attribute the high percentage of shared hotspots to common Prdm9 alleles, but they should also indicate the similar genetic background.

Yes we agree with the reviewer. We rephrased at line 510-513:

*“Only closely related populations shared a substantial fraction of their hotspots. This overlap probably reflects their similar genetic background (low F_ST_), and in particular the fact that they may share similar sets of Prdm9 alleles recognizing common binding DNA motifs.”*

10. Since they have the prdm9 sequences of the alleles, looking for the predicted motif to compare will be informative.

Although motifs can be predicted, the accuracy is low, this can be seen from the Prdm9 mouse data (for instance Smagulova et al 2016 figS4; it also applies to CTCF, see Persikhov and Singh 2014, Fig5). It appears that some zinc fingers are more important than others for DNA binding, and this is not detected by the in silico algorithm, even if an overlap could be detected between in silico and in vivo data.

We did the in silico prediction but did not include them as to our point of view they are not informative. We show them below for allele PRDM9 allele 1 and 2 for the reviewer. Of note, they do not match the motifs identified (Fig3C).

Reported motifs show strange pattern given previous literature, especially the second has high percentage of T. Aren't motifs enriched in GC?

It is true that the motifs we identified in rainbow trout are more AT-rich than the ones that have been reported in humans or mice. However, given the limited number of species for which the binding motifs of PRDM9 have been determined experimentally (only 3), it is difficult to draw any general conclusion regarding the propensity of PRDM9 to target GC-rich motifs (see also see our response to the comment of reviewer #3). Of note, the binding sites of KRAB zinc finger proteins show a wide range of GC content (Imbeault et al. 2017).

11. Authors do not show examples of histological sections from testis, this will be useful to interpret meiosis progression in the samples used.

We agree with the reviewer, and having histological information was indeed important for this project. We now provide typical examples of histological sections (S26 Fig). The histology shows how we can identify the different spermatogenic cells (spermatogonia, spermatocytes, spermatids and spermatozoa), and how we can detect prophase spermatocytes by immunostaining with either anti-SYCP3, anti-SMC3 or anti-DMC1 antibody.

We completed the SUPPLEMENTARY METHODS to mention this point, page 4: “*To validate the presence of meiotic cells in stage III testes, immunostaining against SYCP3, SMC3 and DMC1 was carried out on tissue sections (S26 Fig).*”

and we added a section *‘Immunostaining’* (page 5) to detail this experiment.

12. As for the genome assemblies used, it will be informative to provide stats on genome continuity; how many scaffolds and how large; are all scaffolds assigned to chromosomes?

We added a table in the supplementary material (S12 Table), providing assembly statistics of the reference genome of *O. kisutch*, *O. mykiss* and *S. salar* that were used to map the population resequencing data for the LD-based recombination landscapes and the ChIP-Seq DMC1 peaks. This Table is mentioned in the main text (in the *Variant calling s*ection of the Methods, line 916).

Minor points:

*	Line 72: Please, specify which Mus subspecies; there are several works describing differences between them.

We agree and the study we refer to (Smagulova et al) is based on analysis in *M. m. musculus*, *domesticus* and *castaneus*. We thus keep our text and refer to *Mus musculus* in Line 72

*	Line 178: Ts3R event (Ss4R)?

This was a mistake. We modified the legend of Fig. 1, and this sentence has been removed.

*	Line 205: Please, include the reference to 'other species'.

We included a reference to a review on Prdm9: Grey et al 2013

*	Lines 285-295: This section is hard to follow.

We reformulated this paragraph (line R4_L285-295).

*	Line 354: four out of five salmonids… which ones?

We rephrased (line 450 "*Conversely, in all the five salmonid populations analyzed, except the GP population, GC content tended to decrease close to hotspots (S16B Fig).*”

*	Data support statement of Lines 423-425 needs to be included.

We now cite Lien et al. 2016 (line 469 of the revised ms).

*	Explain better the percentages of Lines 410 and 411.

We rephrased this sentence (line 455):

“*The salmonid genomes contain a high density of TEs (covering ~50% of the genome), among which Tc1/mariner is the most abundant superfamily (>10% of TEs).*”

*	Population abbreviations are not indicated until methods.

We have included the full species name at the first occurrence. Population abbreviations were indicated at their first occurrence at line 347

*	Line 458-459: Please, include number of hotspots, not just percentages.

We added the number of shared hotspots line 497

“*We estimated that only 6.2% of hotspots (n=1298) were shared by O. kisutch and O. mykiss, which diverged from their common ancestor about 16 Myr ago (119).*”

and line 502:

“*Similarly, the two genetically differentiated lineages of S. salar only shared 10.3% (GP vs. BS, FST = 0.26, n=1793) and 11.2% (GP vs. NS, FST = 0.28, n=1671) of their hotspots, with a weak recombination rate increase at the alternate lineage hotspots (Fig 5B, S23B-C Fig). Conversely, the two closely related BS and NS S. salar populations (FST = 0.02) shared 26.3% of their hotspots (n=4421), which was much more than expected by chance (Fig 5C, S23D Fig).*”

*	Line 488: Missing reference.

We added a reference to Myers 2009.

*	Line 560: Perhaps will be more informative to include relevant references of recombination analysis in wild mice having prdm9 allelic variation.

Although the two studies cited on line 560 (now line 609) do not directly analyze the variations due to Prdm9, they do discuss the potential contribution of Prdm9 on these variations. These references can therefore be interesting to the reader. To our knowledge there is no other study directly assessing the role of Prdm9 variation on the pattern of recombination genome wide in wild mice populations. Direct data is however available in humans and chimps, and we have therefore added the relevant references.

*	Line 623: it is stated that …"may be even older", but in the intro (Line X) they already citate papers that indicate that prdm9 was ancestral to all animals.

We agree with the reviewer, we have rephrased the sentence to clarify its content (line 733-735):

“*The main conclusion is that the function of PRDM9 in specifying recombination sites most likely existed in the common ancestor to vertebrates, and might be even older*.”

(also note that the third paragraph of the introduction has been extensively revised)

*	Line 641: "a recent study in mammals…", please indicate the reference.

This was the reference 131 mentioned at the end of the sentence. We have now moved it immediately after the word ‘mammals’ (line 756).

*	Line 699: Is paralog alfa2.2 located in different chromosomes for S Salar and O mykiss? Chr 7 and 17?

Yes it is, and is located on chromosome 5 for *O. kisutch*. These three chromosomes are orthologous chromosomes (Sutherland et al., 2016). The chromosome location of each paralogous copy is now indicated in Table S1.

# References cited in our reply to the reviewers

Auton A, Fledel-Alon A, Pfeifer S, Venn O, Segurel L, Street T, Leffler EM, Bowden R, Aneas I, Broxholme J, Humburg P, Iqbal Z, Lunter G, Maller J, Hernandez RD, Melton C, Venkat A, Nobrega MA, Bontrop R, Myers S, Donnelly P, Przeworski M and McVean G 2012 A Fine-Scale Chimpanzee Genetic Map from Population Sequencing. Science 336: 193–198. <https://doi.org/10.1126/science.1216872>

Auton A, Rui Li Y, Kidd J, Oliveira K, Nadel J, Holloway JK, Hayward JJ, Cohen PE, Greally JM, Wang J, Bustamante CD and Boyko AR 2013 Genetic Recombination Is Targeted towards Gene Promoter Regions in Dogs. PLoS Genetics 9: e1003984. <https://doi.org/10.1371/journal.pgen.1003984>

Booker TR, Ness RW and Keightley PD 2017 The Recombination Landscape in Wild House Mice Inferred Using Population Genomic Data. Genetics 207: 297–309. <https://doi.org/10.1534/genetics.117.300063>

Brick K, Smagulova F, Khil P, Camerini-Otero RD and Petukhova GV 2012 Genetic recombination is directed away from functional genomic elements in mice. Nature 485: 642–645. <https://doi.org/10.1038/nature11089>

Cohen NM, Kenigsberg E and Tanay A 2011 Primate CpG Islands Are Maintained by Heterogeneous Evolutionary Regimes Involving Minimal Selection. Cell 145: 773–786. <https://doi.org/10.1016/j.cell.2011.04.024>

Frazer et al. 2007 A second generation human haplotype map of over 3.1 million SNPs. Nature 449: 851–861. <https://doi.org/10.1038/nature06258>

Imbeault M, Helleboid P-Y and Trono D 2017 KRAB zinc-finger proteins contribute to the evolution of gene regulatory networks. Nature 543: 550–554. <https://doi.org/10.1038/nature21683>

Jiang C, Han L, Su B, Li W-H and Zhao Z 2007 Features and Trend of Loss of Promoter-Associated CpG Islands in the Human and Mouse Genomes. Molecular Biology and Evolution 24: 1991–2000. <https://doi.org/10.1093/molbev/msm128>

Joseph J, Prentout D, Laverré A, Tricou T and Duret L 2024 High prevalence of PRDM9-independent recombination hotspots in placental mammals. Proceedings of the National Academy of Sciences 121: e2401973121. <https://doi.org/10.1073/pnas.2401973121>

Hoge C, de Manuel M, Mahgoub M, Okami N, Fuller Z, Banerjee S, Baker Z, McNulty M, Andolfatto P, Macfarlan TS, Schumer M, Tzika AC and Przeworski M 2024 Patterns of recombination in snakes reveal a tug-of-war between PRDM9 and promoter-like features. Science 383: eadj7026. <https://doi.org/10.1126/science.adj7026>

Kawakami T, Mugal CF, Suh A, Nater A, Burri R, Smeds L and Ellegren H 2017 Whole-genome patterns of linkage disequilibrium across flycatcher populations clarify the causes and consequences of fine-scale recombination rate variation in birds. Molecular Ecology 26: 4158–4172. <https://doi.org/10.1111/mec.14197>

Schield DR, Pasquesi GIM, Perry BW, Adams RH, Nikolakis ZL, Westfall AK, Orton RW, Meik JM, Mackessy SP and Castoe TA 2020 Snake Recombination Landscapes Are Concentrated in Functional Regions despite PRDM9. Molecular Biology and Evolution 37: 1272–1294. <https://doi.org/10.1093/molbev/msaa003>

# New Supplementary Figure and Table numbers

| Figure number in original manuscript | Figure number in reviewed manuscript | Table number in original manuscript | Table number in reviewed manuscript |
| --- | --- | --- | --- |
| S1 | S27 | S1 | S7 |
| S2 | S28 | S2 | S8 |
| S3 | S1 | S3 | S9 |
| S4 | Removed | S4 | S10 |
| S5 | S3 | S5 | S11 |
| S6 | S4 | S6 | S4 |
| S7 | S5 | S7 | S1 |
| S8 | S6 | S8 | S2 |
| S9 | S7 | S9 | S3 |
| S10 | S8 | S10 | S13 |
| S11 | S9 | New Table 1 | S5 |
| S12 | S10 | New Table 2 | S6 |
| S13 | S11 | New Table 3 | S12 |
| S14 | S12 |  |  |
| S15 | S13 |  |  |
| S16 | Removed |  |  |
| S17 | S14 |  |  |
| S18 | S15 |  |  |
| S19 | S16 |  |  |
| S20 | S17 |  |  |
| S21 | S18 |  |  |
| S22 | S19 |  |  |
| S23 | S20 |  |  |
| S24 | S21 |  |  |
| S25 | S22 |  |  |
| S26 | S23 |  |  |
| S27 | S24 |  |  |
| S28 | S25 |  |  |
| New Figure 1 | S2 |  |  |
| New Figure 2 | S26 |  |  |
| New Figure 3 | S29 |  |  |
| New Figure 4 | S30 |  |  |
