# Supplementary material for: PRDM9 drives the location and rapid evolution of recombination hotspots in salmonid fish
Source: PLoS Biol. 2025 Jan 6;23(1):e3002950. doi: 10.1371/journal.pbio.3002950 (PMC11703093; doi:10.1371/journal.pbio.3002950)
Supplement: Attachment — Submitted filename: Answerto reviewers_secondroundv3.docx [file pbio.3002950.s047.docx]

Montpellier, November 18^th^, 2024

We thank the reviewers for their time and providing a constructive evaluation of our revised version of the manuscript. We are pleased by the positive comments and are looking forward for the potential publication of our manuscript in PLoS Biology.

The original comments are written in blue, our reply in black, with the in-text modifications between commas and italicized.

Reviewer #1:

The authors have responded to all my concerns and suggestions.

We thank the reviewer for his/her evaluation

Reviewer #2:

[identifies herself as Dr. Linda Odenthal-Hesse]

I have reviewed the revised manuscript "PBIOLOGY-D-24-00615_R2" titled "PRDM9 drives the location and rapid evolution of recombination hotspots in salmonids by Marie Raynaud et al."

The authors have addressed my previous concerns and recommendations with great care, and I am fully satisfied with the revisions. I also feel that the comments made by other reviewers were carefully addressed. This is a well constructed paper and I recommend publication.

We thank the reviewer for her evaluation

Reviewer #4:

The authors have satisfactorily addressed my initial comments. The manuscript is now much clearer, with more coherent argumentation and a stronger effort to discern the differences between species. I believe this paper makes a significant contribution to the field, and with a few minor revisions, it will be ready for publication. Minor comments.

*	Line 257, the percentage doesn't should be notified in % rather than decimals?

Yes, thank you, this has been revised.

*	The sequence of the identified prdm9 alleles will be reported in fasta format? I can see in Zenodo some processed datasets such as VCF files, LD-based recombination maps and hotspots, PRDM9 protein sequences and multiple alignments, but not allelic variants

All fasta are now included in Zenodo (PRDM9_Alleles.fa)

*	Line 426, "On average, 90% of the total recombination appeared to be concentrated in 20% of the genome, a higher rate than what was observed in human and chimpanzee (29, 72) and slightly higher than what we observed in sea bass (Table 1, S12 Fig, S2 Table). This heterogeneity was largely driven by the presence of recombination hotspot" - From the text, I understand that the differences between sea bass and other salmonids are related to the presence of recombination hotspots and PRDM9. However, how do they explain why the recombination rate is higher in sea bass than in humans and chimpanzees?

As mentioned in the discussion (section 'Comparison of recombination landscapes between vertebrates with or without a full-length PRDM9’), our observations suggest that recombination is generally more concentrated into hotspots in species having a full-length PRDM9 than in species that lack a full-length PRDM9, but we do not claim that this is always the case.

In fact, this question is addressed in detail in the discussion (lines 637-655 of the revised manuscript), based on the comparison of the heterogeneity of recombination landscapes across 18 species (10 species with a full-length PRDM9, and 8 without). In each genome, we measured the fraction of recombination in the 20% of the genome with the highest recombination rate (we note this index F20). Among species that lack a full-length PRDM9, F20 ranges from 57% in *Ficedula hypoleuca* (Pied flycatcher) to 85% in the seabass (average=70%, see S5 Table). Among species with a full-length PRDM9, F20 ranges from 64% in the Rhesus Macaque to 99% in Vervet Monkeys (average=84%). The difference between the two set of species is significant (t test p-value=0.011), but the two distributions largely overlap. This implies that PRDM9 is not the sole factor determining the heterogeneity of recombination landscapes. The reasons for the variation in the usage of PRDM9-independents hotspots are not known.

*	Line 418-419, there is an inconsistency between the recombination rates in the text and in the table 1 (either 0.012 or 0.0012)

Sorry, the correct value is 0.012. Table 1 has been revised.

*	Figures are difficult to see, likely caused by the PDF format. Specifically, Figure 1A, which contains a lot of information and species names, is hard to read. Additionally, Figure 4, panels B and C, seem confusing. The blue and purple colors of the TSS in and out CGI bars are uniquely colored, but their legend is missing in the panels. Overall, the information and main point of Figure 4 is hard to interpret.

We understand and are aware that Fig1 is complex and takes time to read. With the format and the colour code, we think that one main difference between the paralogs, which is that some have lost the KRAB domain (or more), stands out clearly. We also think it is important to keep a large number of paralogs as shown such as to highlight the complexity and diversity which is one intrinsic and important property of *Prdm9*. Presenting a simplified version for instance would lead to the loss of important information.

We think it is important to highlight TSS in and out CGI since those are the genomic features most relevant for comparing PRDM9-dependent and -independent recombination, and this is the central point presented in this figure. We have now included in the legend the colour code used. We are sorry for the low resolution, our original figure is of high resolution, and thus should be easy to read in the final version.
